# A one-pot synthesis of 3-trifluoromethyl-2-isoxazolines from trifluoromethyl aldoxime

**DOI:** 10.3762/bjoc.9.275

**Published:** 2013-11-07

**Authors:** Raoni S B Gonçalves, Michael Dos Santos, Guillaume Bernadat, Danièle Bonnet-Delpon, Benoit Crousse

**Affiliations:** 1 BioCIS CNRS, Faculté de Pharmacie, Labex-LERMIT, Univ Paris Sud, 5 rue J. B. Clément, 92290 Châtenay-Malabry, France

**Keywords:** aldoxime, amino alcohol, fluorine, isoxazole, isoxazoline, organo-fluorine

## Abstract

Functionalized 3-trifluoromethyl-2-isoxazolines and 3-trifluoromethylisoxazoles were easily prepared from trifluoromethyl aldoxime **2** under mild conditions by using DIB as oxidant. Theoretical studies of the reactivity of trifluoroacetonitrile oxide **4** toward olefins and alkynes were carried out. The 3-trifluoromethyl-2-isoxazolines were ring-opened with NaBH_4_ and NiCl_2_ to yield the corresponding trifluoromethylated γ-amino alcohols.

## Introduction

**Figure 1:**
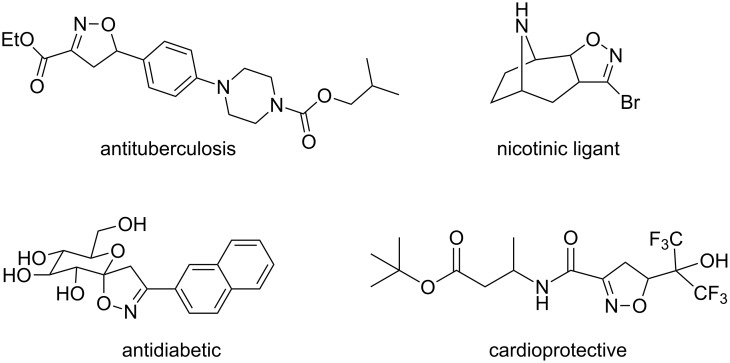
Example of bioactive molecules bearing the 2-isoxazoline nucleus.

2-Isoxazolines are five-membered heterocyclic compounds that have been widely applied in medicinal and organic chemistry. This nucleus is frequently found in natural products [1–4], bioactive molecules [5–6] (Figure 1) and can be used as bioisosteric transformations of amide bonds in order to provide metabolically stable and more active derivatives [7–11]. Moreover, 2-isoxazolines can be cleaved under various conditions to supply a variety of organic functionalities including γ-amino alcohols [12], β-amino acids [13], β-hydroxy ketones [14–15] and β-hydroxy nitriles [14–15].

Fluorinated compounds play a central role in different branches of chemistry [16]. The incorporation of a fluorine atom into bioactive molecules causes remarkable changes of their physicochemical properties, which allows the development of substances with improved pharmacological characteristics. Some examples are the synthesis of modified amino acids and peptides, carbohydrates, natural products and the development of more selective enzyme inhibitors [17–21]. Another powerful area, yet a somewhat less utilised role for fluorine is as a tag for ^19^F NMR that offers several analytical advantages including speed, sensitivity and selectivity [22–23]. Fluorinated molecules have served as valuable ^19^F NMR probes in high-throughput screening, drug metabolism and protein binding experiments as well as in assessing gene expression [24].

**Scheme 1:**
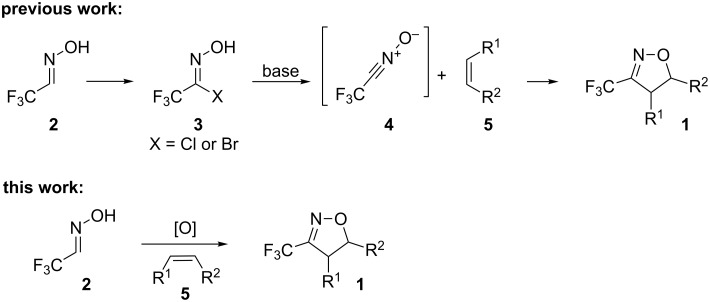
Synthesis of 3-trifluoromethyl-2-isoxazolines.

Nevertheless, the preparation of 3-trifluoromethyl-2-isoxazolines **1** has not been extensively studied so far. In the literature only a few examples of the preparation of these derivatives through a multistep procedure are described (Scheme 1) [25]. Initially, trifluoromethyl aldoxime **2** is halogenated to give a volatile trifluoroacetohydroxymoyl chloride or bromide **3**, which is usually isolated in low yields. Reaction of intermediate **3** with a base provides trifluoroacetonitrile oxide **4**, which can be reacted with olefins (such as styrene, allyl derivatives, etc.) through a 1,3-dipolar cycloaddition to give the desired product. Therefore, the development of a straightforward and mild general procedure to access these valuable derivatives remains of great importance. In the present work, we describe a simple and efficient metal-free protocol for the oxidation of trifluoromethyl aldoxime **2** into trifluoroacetonitrile oxide **4** and a one-pot synthesis of **1** through in situ cyclization of **4** with different dipolarophiles (Scheme 1).

## Results and Discussion

**Scheme 2:**
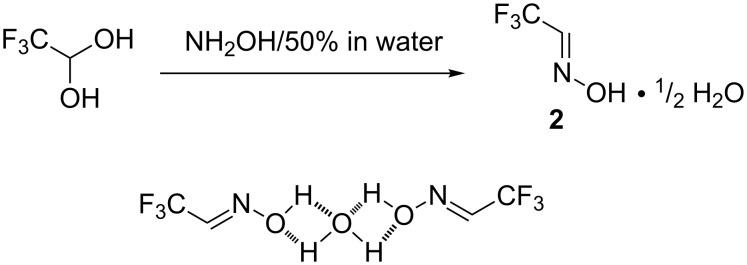
Synthesis of aldoxime **2**.

Initially, another procedure for the preparation of the trifluoroacetaldehyde oxime **2** was developed. In a previous work [25], **2** was obtained as an etherate complex from the reaction between 2,2,2-trifluoroethane-1,1-diol (TFAL) and hydroxylamine hydrochloride. In our work, reaction of TFAL and an aqueous solution of hydroxylamine (50 wt %) yielded the desired product, which was isolated as a complex of two molecules of aldoxime with one molecule of water after distillation in 70–80% yield (Scheme 2).

In the recent literature, different conditions have been developed for the direct oxidation of aldoximes [26–31]. Recently, commercially available reagents have been employed under metal-free conditions. A group [27] reported that the hypervalent iodine reagents (diacetoxyiodo)benzene (DIB) and phenyliodine bis(trifluoroacetate) (PIFA) could successfully promote the oxidation of aldoximes to the corresponding nitrile oxide. Those reagents exhibit potent oxidizing properties, comparable to heavy-metal reagents, but with several advantages such as low toxicity, high availability and the possibility to be utilized under mild conditions [32]. Then, we decided to verify their applicability in the oxidation of **2** despite the presence of water. We first screened different oxidative reagents and conditions for the oxidation step and allylbenzene (**5a**) was chosen as dipolarophile. Our studies for this process are summarized in Table 1.

**Table 1:** Effect of different conditions on the reaction between trifluoromethyl aldoxime **2** and allylbenzene (**5a**).

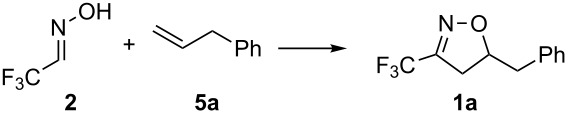			

entry	reaction conditions	time	isolated yield (%)

1	DIB, TEA, MeOH	2 h	complex mixture
2	DIB, HFIP	2 h	complex mixture
3	DIB, CH_2_Cl_2_	overnight	55
4	PIFA, CH_2_Cl_2_	overnight	16

When DIB was used with triethylamine (TEA) and methanol as solvent, the formation of a complex mixture was observed (Table 1, entry 1). This is probably due to the nucleophilic addition of methanol to the highly electrophilic trifluoroacetonitrile oxide. The utilization of the less nucleophilic alcohol hexafluoroisopropanol (HFIP) [33–34] led to the formation of a complex mixture (Table 1, entry 2). The oxidation of **2** with PIFA in CH_2_Cl_2_ afforded the product in only 16% yield (Table 1, entry 4). Better results were obtained by employing DIB in CH_2_Cl_2_ as solvent, after which the product could be isolated in an acceptable yield (55%, Table 1, entry 3). [Bis(acetoxy)iodo]benzene (DIB) is a weaker oxidant than PIFA. When the oxidation is carried out with DIB, weak acetic acid instead of strong trifluoroacetic acid is liberated, and the decomposition of the oxazolines is avoided.

**Figure 2:**
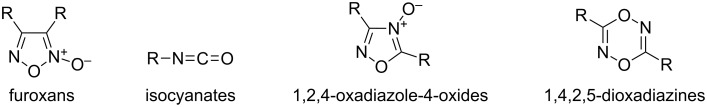
Dimerization and isomerization products from nitrile oxides.

Faced with the moderate yield of **1a**, we followed the reaction by using ^19^F NMR. The measurement of the crude mixture with ^19^F NMR revealed the presence of a side product and despite the total consumption of the aldoxime, a small amount of allylbenzene remained. It is known that nitrile oxides can dimerize or isomerize to yield different products, such as furoxans, isocyanates, 1,2,4-oxadiazoles and 1,4,2,5-dioxadiazines (Figure 2).

**Figure 3:**
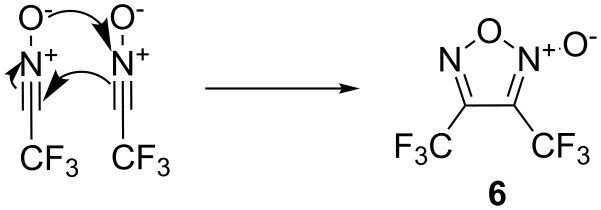
Dimerization of **4** yielding bis(trifluoromethyl)furoxan **6**.

We thus postulated that a competition between the cycloaddition reaction and the dimerization or isomerization pathways could occur. Aiming to confirm our hypothesis we carried out the reaction without the presence of allylbenzene. After 12 h, **2** was completely consumed with the exclusive formation of the previously observed side product. However, attempts to isolate this product failed due to its high volatility. It was therefore co-distilled with CH_2_Cl_2_ and the resulted solution was analyzed by ^19^F NMR coupled and decoupled with proton, ^19^F,^19^F-COSY and ^19^F,^19^F-NOESY (see Supporting Information File 1). Data confirmed the formation of bis(trifluoromethyl)furoxan **6** (Figure 3) already synthesized by Middleton [35].

Considering that the best results were reached utilizing DIB and CH_2_Cl_2_ as solvent, these conditions were selected for further optimizations. After several investigations, we could verify that employing two equivalents of aldoxime **2** and two equivalents of DIB led to complete conversion of the starting olefin. The product could be isolated in good yield (76%, see below in Table 2, entry 1). We can note here that water complexed with **2** did not alter the reaction rate.

**Table 2:** Synthesis of 3-trifluoromethyl-2-isoxazolines and isoxazoles by reaction between aldoxime **2** and olefins or alkynes in the presence of DIB.

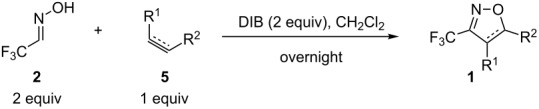			

entry	substrate	product	isolated yield (%)

1	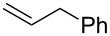 **5a**	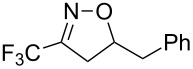 **1a**	76
2	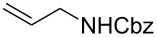 **5b**	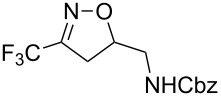 **1b**	90
3	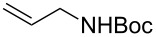 **5c**	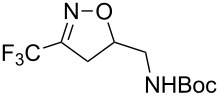 **1c**	91
4	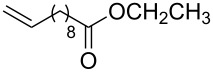 **5d**	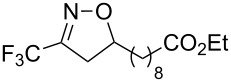 **1d**	64
5	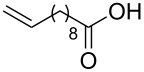 **5e**	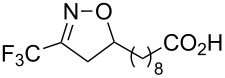 **1e**	51
6	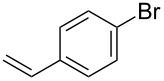 **5f**	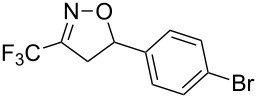 **1f**	56
7	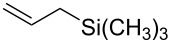 **5g**	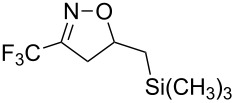 **1g**	82
8	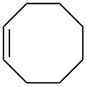 **5h**	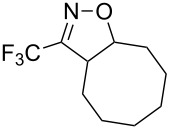 **1h**	74
9	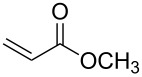 **5i**	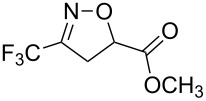 **1i**	24
10	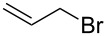 **5j**	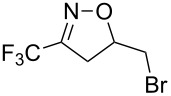 **1j**	20
11	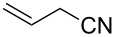 **5k**	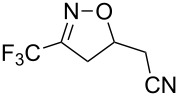 **1k**	traces
12	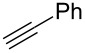 **5l**	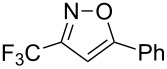 **1l**	53
13	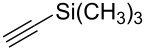 **5m**	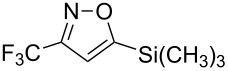 **1m**	50
14	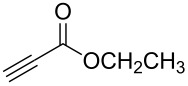 **5n**	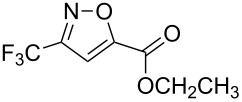 **1n**	traces

Having optimized reagents and conditions, the scope of the reaction was explored with regard to the substrates (Table 2). As observed in the earliest works [11], the cycloaddition of **4** with terminal olefins led to the corresponding 3-trifluoromethyl-5-substituted-2-isoxazoline **1** with complete regioselectivity. No trace of the regioisomer 3-trifluoromethyl-4-substituted-2-isoxazoline could be detected even when yields of cycloadducts were low. From functionalized olefins such as NH–Cbz and NH–Boc allylamines the desired product could be isolated in excellent yields (90% and 91%, respectively, Table 2, entries 2 and 3). Interestingly, the protecting groups were not cleaved, which indicated that reaction conditions are very mild. With allyltrimethylsilane oxazoline **1g** was obtained in good yield (82%). From ester and acid derivatives of undecen, isoxazolines were obtained in good yields too (64% and 51%, respectively, Table 2, entries 4 and 5). With *para*-bromostyrene isoxazoline **1f** was obtained in 56% yield. On the other hand, a good yield was reached for disubstituted olefin **5h** (Table 2, entry 8). However a complete lack of reactivity was observed for the reaction with the electron-poor olefins **5i**, **5j** and **5k** (Table 2, entries 9–11). The reaction was also carried out with alkynes, providing 3-trifluoromethyl-5-substituted-isoxazoles. Moderate yields were obtained for the reaction with phenylacetylene (**5l**) and trimethylsilylacetylene (**5m**, Table 2, entries 12 and 13). However, the electron poor alkyne **5n** was unreactive towards trifluoroacetonitrile oxide **4**.

**Figure 4:**
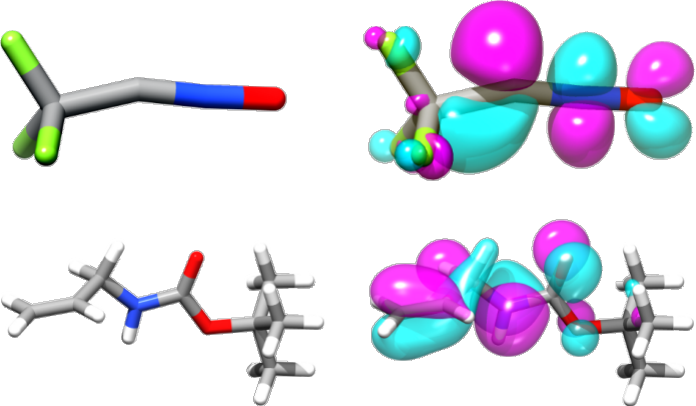
Depiction of the geometry (left column) and isodensity surface of the reacting frontier molecular orbitals (FMO) at 50% probability (right column) of trifluoroacetonitrile oxide **4** (top row) and protected aminoalkene **5c** (bottom row) calculated at the B3LYP/6-31G* level.

1,3-Dipolar cycloaddition reactions have been studied from the theoretical standpoint since the 1970’s onwards [36–37] with an ever-increasing accuracy as computational methods evolved [38]. Assuming that the above-mentioned transformations occur via a concerted mechanism, we decided to perform electronic structure calculations at the B3LYP/6-31G* level in order to further understand the reactivity of the different unsaturated compounds studied in this work against trifluoroacetonitrile oxide **4** (See Supporting Information File 1) [39–43]. Upon energy minimization, a structure with a geometry close to linearity was found for trifluoroacetonitrile oxide (Figure 4), which is consistent with earlier findings [44].

**Figure 5:**
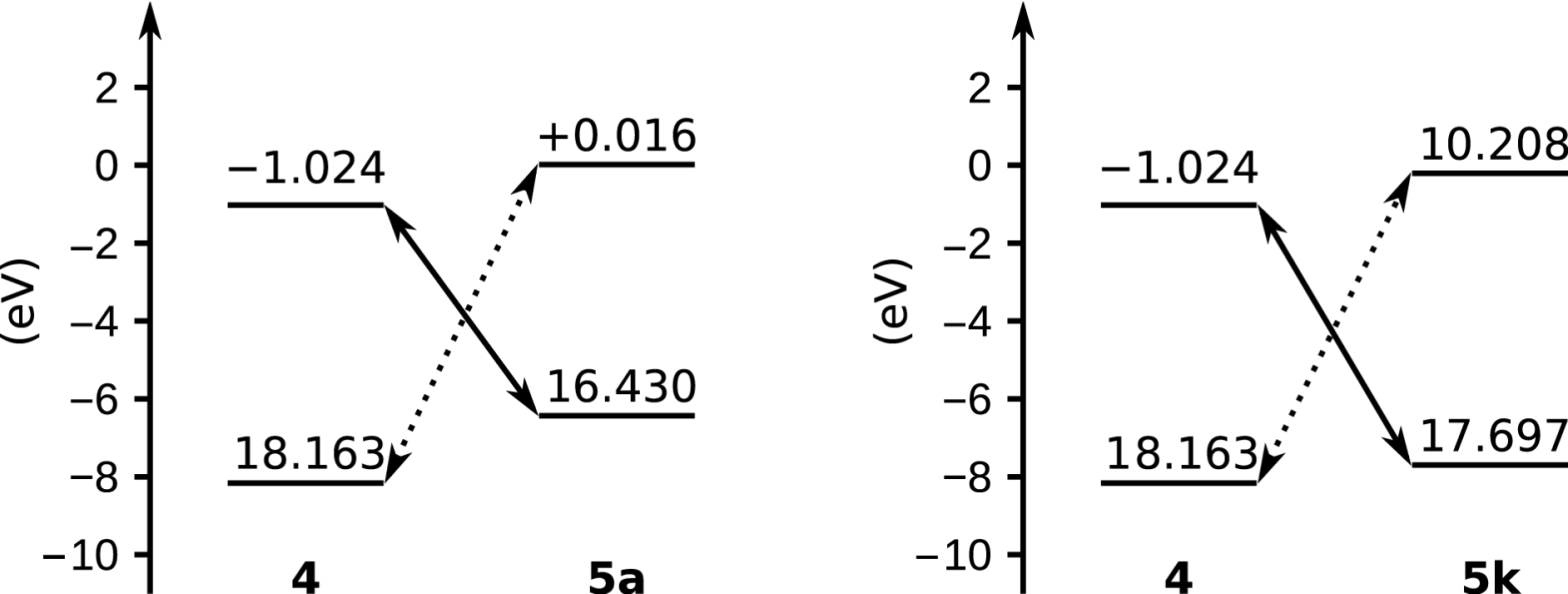
FMO energy levels of dipole **4** and dipolarophiles **5a** and **5k** calculated at the B3LYP/6-31G* level. Continuous and dotted lines indicate the favored (Δ*E* = 5.407 eV for **5a** and 6.673 eV for **5k**) and the disfavored (Δ*E* = 8.178 eV for **5a** and 7.955 eV for **5k**) molecular orbital interactions, respectively.

Comparison of the energy gaps between the frontier molecular orbital levels of the nitrile oxide and those of the alkene partners (Figure 5) suggest that type-III cycloaddition reactions (where the dipole reacts via its LUMO and the dipolarophile via its HOMO) take place for every combination of reactants reported herein [45].

**Figure 6:**
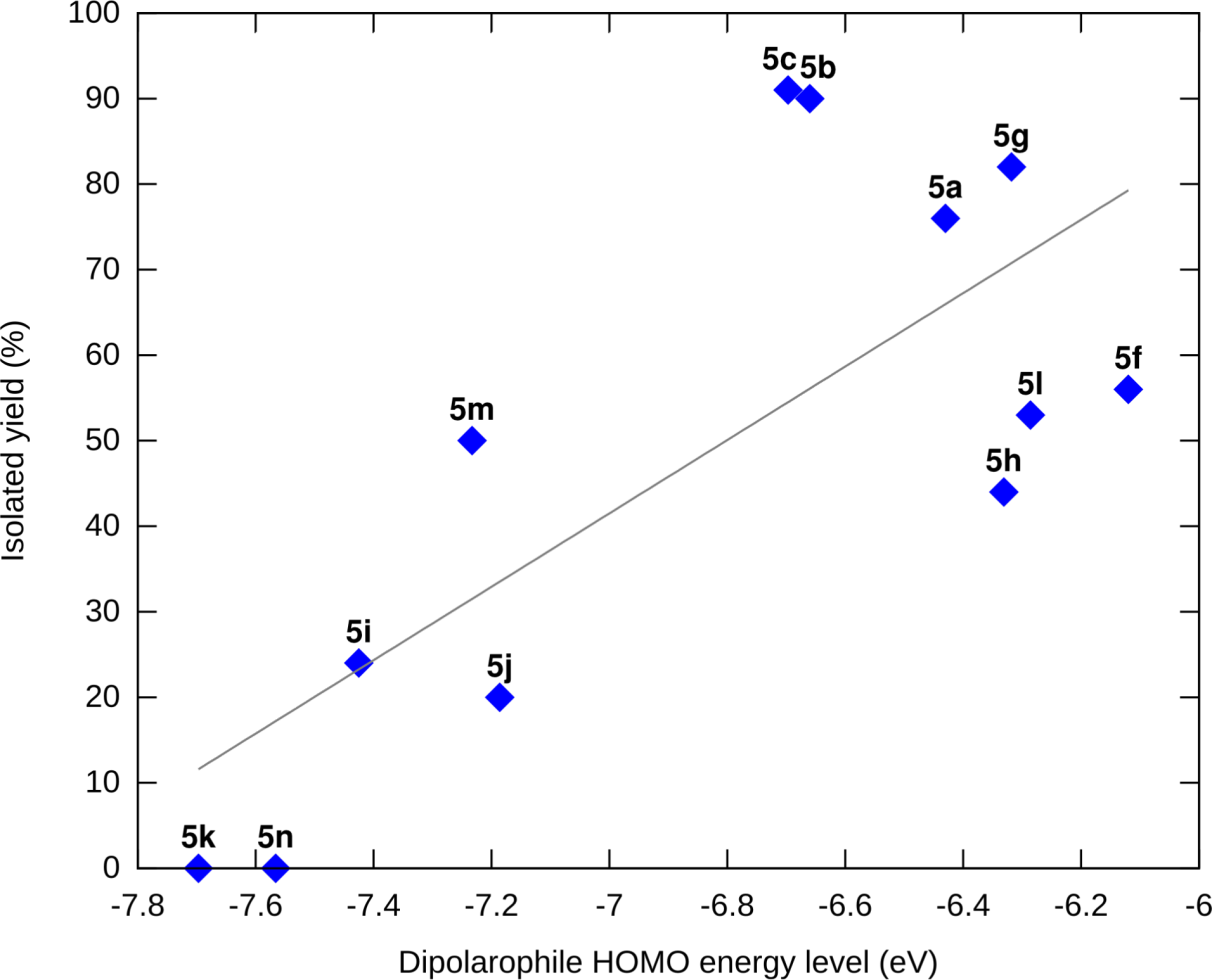
Yields of the cycloaddition reaction plotted against the HOMO energy levels of the dipolarophile partner among **5a**–**c**,**f**–**n**.

In this scenario, with the same dipole, the reactivity is expected to increase with the HOMO energy level of the dipolarophile. A simple scatter plot of yield versus the latter variable confirms and illustrates this trend (Figure 6), which can be interpreted the following way: when the gap between the LUMO energy level of the nitrile oxide and the HOMO energy level of the alkene becomes too high (relatively to 7.139 eV, which is the energy difference for the self-cycloaddition process), the dimerization pathway is favored and the yield of isoxazole drops.

With only a single regioisomer being isolated, we also considered the coefficients (Table 3) and shapes (Figure 4) of frontier molecular orbitals. This data is compatible with the observed regioselectivity [37]. Steric factors can also exert notable influence, but they would guide regioselectivity in the same direction.

**Table 3:** FMO coefficients of the 1,3-dipole **4** and representative dipolarophiles (atomic orbital is indicated in parentheses).

	coefficients	
	
reactant	C_a_	C_b_

**5c**	0.180 (2p*_y_*)	0.133 (2p*_x_*)
**5g**	0.321 (2p*_z_*)	0.246 (2p*_z_*)
**4**^a^	0.434 (2p*_y_*)	0.299 (2p*_y_*)

^a^Columns C_a_ and C_b_ contain coefficients for C and O.

In a subsequent step, we decided to preliminarily study the ring opening reaction of the 3-trifluoromethyl-2-isoxazolines in order to prepare the corresponding trifluoromethylated γ-amino alcohols. The major and almost the only route to synthesize these amino alcohols is the reduction of β-aminocarbonyl compounds prepared from Mannich-type reactions [46–48]. The ring opening of 2-methyl-3-trifluoromethylisoxazolines by utilizing H_2_ and Raney-Ni as catalysts was described by Tanaka and co-workers [49]. However, this methodology is restricted to the synthesis of *N*-methylated amino alcohols. Instead we investigated the reduction and the ring opening of 3-trifluoromethyl-2-isoxazolines **1a** and **1b** in the presence of NaBH_4_ and NiCl_2_ as additives [50]. Under these conditions, a total conversion of the starting material was observed by ^19^F NMR, and products **7a** and **7b** were obtained in moderate yields. However the diastereoisomeric excess was very poor (10% de). The results are reported in Table 4.

**Table 4:** Ring opening reaction of 3-trifluoromethyl-2-isoxazolines **1a** and **1b**.

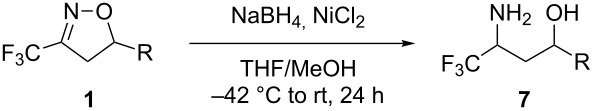				

entry	substrate	product	isolated yield (%)	dr

1	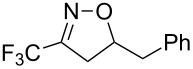 **1a**	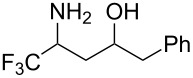 **7a**	50	40:60
2	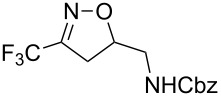 **1b**	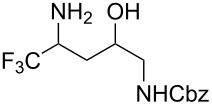 **7b**	52	40:60

## Conclusion

In conclusion, we have developed a simple, mild and efficient one-step procedure for the synthesis of functionalized 3-trifluoromethyl-2-isoxazolines and 3-trifluoromethyl-2-isoxazoles from trifluoromethyl aldoxime **2** by utilizing DIB as oxidant. The applicability of the 3-trifluoromethyl-2-isoxazolines to supply different fluorinated building blocks was demonstrated by the easy ring opening of these intermediates with NaBH_4_ and NiCl_2_, yielding the corresponding trifluoromethylated γ-amino alcohol.

## Supporting Information

File 1 General methods, synthetic procedure, spectroscopic data, ^1^H NMR, ^13^C NMR and ^19^F NMR of compounds of **2, 1a-1j, 1l-1n, 7a, 7b, 6** and computational results.
