# Supplementary material for: A one-pot synthesis of 3-trifluoromethyl-2-isoxazolines from trifluoromethyl aldoxime
Source: Beilstein J Org Chem. 2013 Nov 7;9:2387–94. doi: 10.3762/bjoc.9.275 (PMC3869255; doi:10.3762/bjoc.9.275)
Supplement: File 1 — General methods, synthetic procedure, spectroscopic data, 1H NMR, 13C NMR and 19F NMR of compounds of 2, 1a-1j, 1l-1n, 7a, 7b, 6 and computational results. [file Beilstein_J_Org_Chem-09-2387-s001.pdf]

Supporting Information

for

A one-pot synthesis of 3-trifluoromethyl-2-isoxazolines from trifluoromethyl aldoxime

Raoni S. B. Gonçalves, Michael Dos Santos, Guillaume Bernadat, Danièle Bonnet-Delpon and Benoit Crousse*

Address: BioCIS CNRS, Faculté de Pharmacie, Labex-LERMIT, Univ Paris Sud, 5 rue J.B. Clément, 92290 Châtenay-Malabry, France

Email: Benoit Crousse* - Benoit.crousse@u-psud.fr

* Corresponding author

General methods, synthetic procedure, spectroscopic data, ^{1}H NMR, ^{13}C NMR and ^{19}F NMR of compounds of 2, 1a–j, 1l–n, 7a, 7b, 6 and computational results

Content

1. Characteristics for compounds **2**, **1a-1j**, **1l-1n**, **7a**, **7b**, **6**
2. ^{1}H, ^{13}C and ^{19}F NMR spectra of **2**, **1a-1j**, **1l-1n**, **7a**, **7b** and ^{19}F NMR of **6**
3. Computational results

General information

Commercial reagents were purchased from suppliers and used as received. CH_2Cl_2 was distilled under nitrogen from CaH_2 prior to use. Flash chromatography was performed on commercial 40–63 μm silica gel. Thin layer chromatography was performed on precoated aluminium sheets (0.2 mm). The compounds were visualized under a 254 nm UV light. ^{1}H NMR spectra, ^{19}F NMR spectra and ^{13}C NMR spectra were recorded on apparatus at respectively 200 MHz ^{1}H, 235.6 MHz ^{19}F, 75 MHz ^{13}C or 400 MHz ^{1}H, 376.2 MHz ^{19}F, 100.5 MHz ^{13}C. Chemical shift values (δ) are reported in ppm downfield from Me_4Si (δ 0.0 ppm), $CFCl_3$ or $CDCl_3$ as internal standard (δ 77.0 ppm). Data are reported as follows: chemical shift (δ ppm), multiplicity (s = singlet, d = doublet, t = triplet, q = quartet, m = multiplet), integration, coupling constant (Hz). NMR peak assignments have been made on the basis of HMQC, HMBC, and 1H,1H COSY spectra. Infrared (FT-IR) spectra were performed with neat films.

1. Characteristics for compounds **2**, **1a–j**, **1l–n**, **7a**, **7b**, **6**

General Procedure for the Synthesis 2,2,2-trifluoroacetaldehyde oxime 2 :

2,2,2-trifluoroethane-1,1-diol (22.5 g, 0.194 mol, 1 eq.) was add to an aqueous solution of hydroxylamine (30%) (7.68 g, 0.232 mol, 1.2 eq.). The reaction stirred for 24 h under argon atmosphere. The crude product was purified by a fractional distillation. ^{1}H NMR (300 MHz, $CDCl_3$) 8.7 (s, 1H), 7.5 (q, J = 4.1 Hz, 1H), 2.2 (s, ½ H_2O). ^{13}C NMR (75 MHz, $CDCl_3$) δ 139.7 (q, J = 37.5 Hz), 119.7 (q, J = 270.0 Hz). ^{19}F NMR (188 MHz, $CDCl_3$) δ -65.50 (d, J = 4.2 Hz).

General Procedure for the Synthesis Isoxazoles 1:

At a solution of DIB (2.0 mmol) and olefin (1.0 mmol) in dichloromethane (2 mL) the 2,2,2-trifluoroacetaldehyde oxime **2** (1.0 mmol) in dichloromethane (1 mL) was added slowly at 0°C. The media was warmed at room temperature and upon consumption of starting olefin (TLC, and ^{19}F NMR of the crude). The mixture was concentrated in vacuo and the residue was purified by flash column chromatography (5% ethyl acetate/cyclohexane).

Synthesis of 5-benzyl-3-(trifluoromethyl)-4,5-dihydroisoxazole 1a: colorless oil, 174 mg, 76%; ^{1}H NMR (300 MHz, $CDCl_3$) δ 7.54-7.12 (m, 5H), 5.13 (ddd, J = 14.4, 10.8, 6.6 Hz, 1H), 3.28-3.07 (m, 2H), 2.91 (ddd, J = 11.2, 9.2, 4.0 Hz, 2H). ^{13}C NMR (75 MHz, $CDCl_3$) δ 148.64 (q, J = 37.3 Hz), 135.4, 129.3, 128.7, 127.1, 119.6 (q, J = 271.2 Hz), 84.0, 40.5, 36.3. ^{19}F (188 MHz, $CDCl_3$) δ -67.01 (s). IR (ν,cm^{-1}): 1625. Anal. Calcd for $C_{11}H_{10}F_3NO$: C, 57.64; H, 4.40; N, 6.11. Found : C, 57.65; H, 4.51; N, 5.99.

Synthesis of benzyl (3-(trifluoromethyl)-4,5-dihydroisoxazol-5-yl)methylcarbamate 1b: oil, 272 mg, 90%; ^{1}H NMR (300 MHz, $CDCl_3$) δ 7.39-7.29 (m, 5H), 5.11 (s, 2H), 5.01-4.92 (m, 1H), 3.49-3.41 (m, 2H) 3.22 (dd, J = 11.4, 17.4 Hz, 1H), 2.99 (dd, J = 7.8, 17.7 Hz, 1H). ^{13}C NMR (75 MHz, $CDCl_3$) δ 156.8, 149.2 (q, J = 38.0 Hz), 136.1, 128.5, 128.3, 128.0, 119.7 (q, J = 282.0 Hz), 82.3, 67.2, 43.6, 34.5. ^{19}F (188

MHz, $CDCl_3$) δ -66.92 (s). Anal. Calcd for $C_{13}H_{13}F_3N_2O_3$: C,51.66; H, 4.43 ; N, 9.27 . Found : C, 52.01 ; H, 4.56 ; N, 9.34.

Synthesis of tert-butyl (3-(trifluoromethyl)-4,5-dihydroisoxazol-5-yl)methylcarbamate 1c: white solid, 243 mg, 91%, mp 67-70°C; ^{1}H NMR (300 MHz, $CDCl_3$) δ 4.98 (ddd, *J* = 11.5, 8.0, 4.1 Hz, 1H), 4.87 (s, 1H), 3.44 (m, 2H), 3.24 (dd, *J* = 17.6, 11.1 Hz, 1H), 3.05 (dd, *J* = 17.6, 7.8 Hz, 1H), 1.46 (s, 9H). ^{13}C NMR (75 MHz, $CDCl_3$) δ 156.3, 149.2 (q, *J* = 36.8 Hz), 119.5 (q, *J* = 271.2 Hz), 82.5, 79.9, 43.1, 34.3, 28.1. ^{19}F (188 MHz, $CDCl_3$) δ -67.02 (s). IR (ν,cm^{-1})**:** 1725; 1643. Anal. Calcd for $C_{10}H_{15}F_3N_2O_3$: C, 44.78 ; H, 5.64 ; N, 10.44 . Found : C, 44.70 ; H, 5.62 ; N, 10.57.

Synthesis of ethyl 9-(3-(trifluoromethyl)-4,5-dihydroisoxazol-5-yl)nonanoate 1d: white solid, 206 mg, 64%, mp 30-32°C; ^{1}H NMR (300 MHz, $CDCl_3$) δ 4.82 (m, 1H), 4.11 (q, *J* = 7.1 Hz, 2H), 3.20 (dd, *J* = 17.1, 10.8 Hz, 1H), 2.77 (dd, *J* = 17.1, 8.5 Hz, 1H), 2.28 (t, *J* = 7.5 Hz, 2H), 1.79-1.56 (m, 4H), 1.30 (s, 10H), 1.24 (t, *J* = 7.1 Hz, 3H). ^{13}C NMR (75 MHz, $CDCl_3$) δ 173.8, 148.5 (q, *J* = 37.1 Hz), 119.8 (q, *J* = 270.8 Hz), 83.9, 60.1, 36.9, 34.8, 34.3, 29.2, 29.1, 29.05, 29.0, 25.0, 24.9, 14.2. ^{19}F (188 MHz, $CDCl_3$) δ -67.18 (s). IR (ν,cm^{-1}): 2914, 1725, 1661. Anal. Calcd for $C_{15}H_{24}F_3NO_3$: C, 55.72; H, 7.48; N, 4.33. Found : C, 55.69; H, 7.53; N, 4.41.

Synthesis of 9-(3-(trifluoromethyl)-4,5-dihydroisoxazol-5-yl)-nonanoic acid 1e: white solid, 150 mg, 51%, mp 57-60°C; ^{1}H NMR (200 MHz, $CDCl_3$) δ 9.8 (large s, 1H), 4.8 (m, 1H), 3.2 (dd, *J* = 6.4, 1.3 Hz, 1H), 2.78 (dd, *J* = 16.5, 7.8 Hz, 1H), 2.3 (t, *J* = 7.4 Hz, 2H), 1.82-1.5 (m, 4H), 1.47-1.16 (m, 10H). ^{13}C NMR (75 MHz, $CDCl_3$) δ 180.0, 148.8 (q, *J* = 37.3 Hz), 119.8 (q, *J* = 274.8 Hz), 83.9, 36.9, 34.8, 33.9, 29.1, 29.0, 28.9, 25.0, 24.6. ^{19}F (188 MHz, $CDCl_3$) δ -66.97 (s). IR (ν,cm^{-1}): 2851, 1711, 1623. Anal. Calcd for $C_{13}H_{20}F_3NO_3$: C, 52.88 ; H, 6.83 ; N, 4.74 . Found : C, 52.75 ; H, 6.94 ; N, 4.85.

Synthesis of 5-(4-bromophenyl)-3-(trifluoromethyl)-4,5-dihydroisoxazole 1f: colorless oil, 164 mg, 56%; ^{1}H NMR (300 MHz, $CDCl_3$) δ 7.56 (d, *J* = 8.3 Hz, 2H), 7.22 (d, *J* = 8.3 Hz, 2H), 5.8 (dd, *J* = 11.4, 8.8 Hz, 1H), 3.63 (ddq, *J* = 17.5, 11.5, 1.3 Hz, 1H), 3.14 (ddq, *J* = 17.5, 8.8, 1.3 Hz, 1H). ^{13}C NMR (75 MHz, $CDCl_3$) δ 148.4 (q, *J* = 37.4 Hz), 137.8, 132.2, 127.4, 123.0, 119.5 (q, *J* = 271.3 Hz), 84.0, 39.9. ^{19}F (188 MHz, $CDCl_3$) δ -66.69 (s). IR (ν,cm^{-1})**:** 1629. Anal. Calcd for $C_{10}H_7F_3NOBr$: C, 40.84 ; H, 2.40 ; N, 4.76 . Found : C, 40.86 ; H, 2.49 ; N, 4.82.

Synthesis of 3-(trifluoromethyl)-5-((trimethylsilyl)methyl)-4,5-dihydroisoxazole 1g: yellowish oil, 184 mg, 82%; ^{1}H NMR (300 MHz, $CDCl_3$) δ 4.87 (m, 1H), 3.2 (ddq, *J* = 17.0, 10.3, 1.3 Hz, 1H), 2.7 (ddq, *J* = 17.0, 9.5, 1.3 Hz, 1H), 1.28 (dd, *J* = 14.2, 6.3 Hz, 1H), 1.05 (dd, *J* = 14.2, 8.5 Hz, 1H), 0.09 (s, 9H). ^{13}C NMR (75 MHz, $CDCl_3$) δ 149.7 (q, *J* = 37.2 Hz), 121.0 (q, *J* = 275.1 Hz), 83.1, 39.3, 24.3, 0.2. ^{19}F (188 MHz, $CDCl_3$) δ -67.06 (s). IR (cm-1): 1664. Anal. Calcd for $C_8H_{14}F_3NOSi$: C, 42.65 ; H, 6.26 ; N, 6.22 . Found : C, 42.71 ; H, 6.18 ; N, 6.25.

Synthesis of 3-(trifluoromethyl)-3a, 4, 5, 6, 7, 8, 9,9a-octahydrocycloocta[d]isoxazole 1h: colorless oil, 163 mg, 74%; ^{1}H NMR (300 MHz, $CDCl_3$) δ 4.7 (m, 1H), 3.2 (m, 1H), 2.1-1.9 (m, 2H), 1.7-1.1 (m, 10H). ^{13}C NMR (75 MHz, $CDCl_3$) δ 153.2 (q, *J* = 34.3 Hz), 122.2 (q, *J* = 272.3 Hz), 88.0, 48.4, 29.4,

25.7, 25.5, 25.2, 25.0, 23.3. ^{19}F (188 MHz, $CDCl_3$) δ -63.71 (s). IR (ν,cm^{-1}): 2936,1629. Anal. Calcd for $C_{10}H_{14}F_3NO$: C, 54.29 ; H, 6.38 ; N, 6.33 . Found : C, 54.33 ; H, 6.45 ; N, 6.42.

Synthesis of methyl-3-(trifluoromethyl)-4,5-dihydroisoxazole-5-carboxylate 1i: colourless oil, 47 mg, 24%; ^{1}H NMR (300 MHz, $CDCl_3$) δ 5.3 (dd, *J* = 11.4, 7.9 Hz, 1H), 3.85 (s, 3H), 3.5 (m, 2H). ^{13}C NMR (75 MHz, $CDCl_3$) δ 168.7, 148.6 (q, *J* = 37.5 Hz), 119.2 (q, *J* = 270.2 Hz), 79.6, 53.2, 35.9. ^{19}F (188 MHz, $CDCl_3$) δ -66.51 (s). IR (ν,cm^{-1}): 1730, 1664. Anal. Calcd for $C_6H_6F_3NO_3$: C, 36.56 ; H, 3.07 ; N, 7.11 . Found : C, 36.52 ; H, 3.12 ; N, 7.20.

Synthesis of 5-(bromomethyl)-3-(trifluoromethyl)-4,5-dihydroisoxazole 1j: Yellowish oil, 46 mg, 20%; ^{1}H NMR (200 MHz, $CDCl_3$) δ 5.1 (m, 1H), 3.56 (dd, *J* = 10.8, 4.1 Hz, 1H), 3.45 (dd, *J* = 10.8, 7.2 Hz, 1H), 3.31 (ddq, *J* = 17.7, 10.8, 1.4 Hz, 1H), 3.20 (ddq, *J* = 17.7, 7.2, 1.4 Hz, 1H). ^{13}C NMR (75 MHz, $CDCl_3$) δ 148.4 q, *J* = 37.3 Hz), 119.3 (q, *J* = 269.7 Hz), 81.5, 36.5, 32.0. ^{19}F (188 MHz, $CDCl_3$) δ -66.82 (s). IR (ν,cm^{-1}): 1660. Anal. Calcd for $C_5H_5F_3NOBr$: C, 25.89 ; H, 2.17 ; N, 6.04 . Found : C, 25.95 ; H, 2.22 ; N, 6.21.

Synthesis of 5-phenyl-3-(trifluoromethyl)isoxazole 1l [1]: Yellow solid, 113mg, 53%, mp 40-43°C; ^{1}H NMR (300 MHz, $CDCl_3$) δ 7.82 (m, 2H), 7.5 (m, 3H), 6.74 (s, 1H). ^{13}C NMR (75 MHz, $CDCl_3$) δ 172.4, 156.0 (q, *J* = 38.5 Hz), 131.2, 129.2, 128.6, 126.0, 119.7 (q, *J* = 271.7 Hz), 96.7. ^{19}F (188 MHz, $CDCl_3$) δ -63.65 (s). IR (ν,cm^{-1}): 1661. Anal. Calcd for $C_{10}H_6F_3NO$: C, 56.35 ; H, 2.84 ; N, 6.57 . Found : C, 56.44 ; H, 2.91 ; N, 6.60.

Synthesis of 3-(trifluoromethyl)-5-(trimethylsilyl)isoxazole 1m : colorless oil, 90mg, 50%; ^{1}H NMR (300 MHz, $CDCl_3$) δ 6.65 (s, 1H), 0.4 (s, 9H). ^{13}C NMR (75 MHz, $CDCl_3$) δ 181.7, 154.0 (q, *J* = 37.8 Hz), 120.3 (q, *J* = 270.8 Hz), 109.6, -2.2. IR (ν,cm^{-1}): 1668. ^{19}F (188 MHz, $CDCl_3$) δ -62.66 (s). Anal. Calcd for $C_7H_{10}F_3NO$: C, 40.18 ; H, 4.82 ; N, 6.69 . Found : C, 40.22 ; H, 4.91 ; N, 6.73.

General Procedure for the synthesis of amino alcohols:

Synthesis of 4-amino-5,5,5-trifluoro-1-phenylpentan-2-ol 7a: $NiCl_2.6H_2O$ (0.620 g, 2.62 mmol, 3 eq.) and 5-benzyl-3-(trifluoromethyl)-4,5-dihydroisoxazole (300 mg, 1.31 mmol, 1 eq.) were dissolved in 3:1 mixture of MeOH/ THF at -42ºC. After 10 min of stirring, $NaBH_4$ (0.495g, 2.618 mmol, 10 eq.) was upon with the reaction, the mixture immediately turned black. After 3 days The mixture was concentrated in vacuo and the residue was purified by flash column chromatography (DCM/MeOH); white solid, 154 mg, 50%, mp 88-90°C; ^{1}H NMR (400 MHz, $CDCl_3$) δ *Syn*: 7.34-7.21 (m, 5H), 4.1 (m, 1H), 3.36 (m, 1H), 2.87 (dd, *J* = 4.0, 12.0 Hz, 1H), 2.74 (dd, *J* = 8.0, 12.0 Hz, 1H), 1.88 (m, 1H), 1.46 (m, 1H). *Anti*: 7.3-7.2 (m, 5H), 4.184.15 (m, 1H), 3.55 (m, 1H), 2.81 (d, *J* = 8.0 Hz, 2H), 1.80 (1H, m), 1.62 (1H, m), ^{13}C NMR (100 MHz, $CDCl_3$) δ 129.6, (129.5), 128.8 (128.6), 126.8 (126.6), 72.9, 68.8, 54.5 (q, *J* = 30.0 Hz) (50.6 (q, *J* = 29.4 Hz), 44.3 (44.1), 35.1 (34.2), CF_3 n.o. . ^{19}F (188 MHz, $CDCl_3$) δ -77.2 (d, *J* = 7.4 Hz), -77.9 (d, *J* = 7.1 Hz). Anal. Calcd for $C_{11}H_{14}F_3NO$: C, 56.65 ; H, 6.05 ; N, 6.01 . Found : C, 56.71 ; H, 6.12 ; N, 5.95.

Synthesis of benzyl 4-amino-5,5,5-trifluoro-2-hydroxypentylcarbamate 7b: 207 mg, 52%,; ^{1}H NMR (400 MHz, MeOD) δ 7.35-7.29 (m, 12H), 5.08 (s, 4.5H), 3.92-3. 86 (m, 1.6H), 3.45-3.42 (m, 1.6H), 3.23-

3.05 (m, 4.4H), 1.85 (dt, J = 4.0, 8.0 Hz, 1H), 1.69-1.63 (m, 1H), 1.56-1. 44 (m, 2.7H), ^{13}C NMR (100 MHz, MeOD) δ 159.1, 138.4, 129.4, 129.0, 128.8, 128.4 (q, *J* = 280.7 Hz), 127.8 (q, *J* = 280.7 Hz), 70.6, 67.5, 67.4, 53.5 (q, *J* = 29.2 Hz), 51.4 (q, *J* = 29.1 Hz), 48.1, 47.7, 35.4, 34.6. ^{19}F (188 MHz, $CDCl_3$) δ -80.52 (d, *J* = 7.5 Hz) 59%, -80.36 (d, *J* = 7.9 Hz) 41%. Anal. Calcd for $C_{13}H_{17}F_3N_2O_3$: C, 50.98 ; H, 5.59 ; N, 9.15 . Found : C, 51.05 ; H, 5.63 ; N, 9.22.

2. ^{1}H, ^{13}C and ^{19}F NMR spectra of **2**, **1a–1j**, **1l–1n**, **7a**, **7b** and ^{19}F NMR of **6**

2,2,2-trifluoroacetaldehyde oxime (**2**)

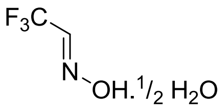

2

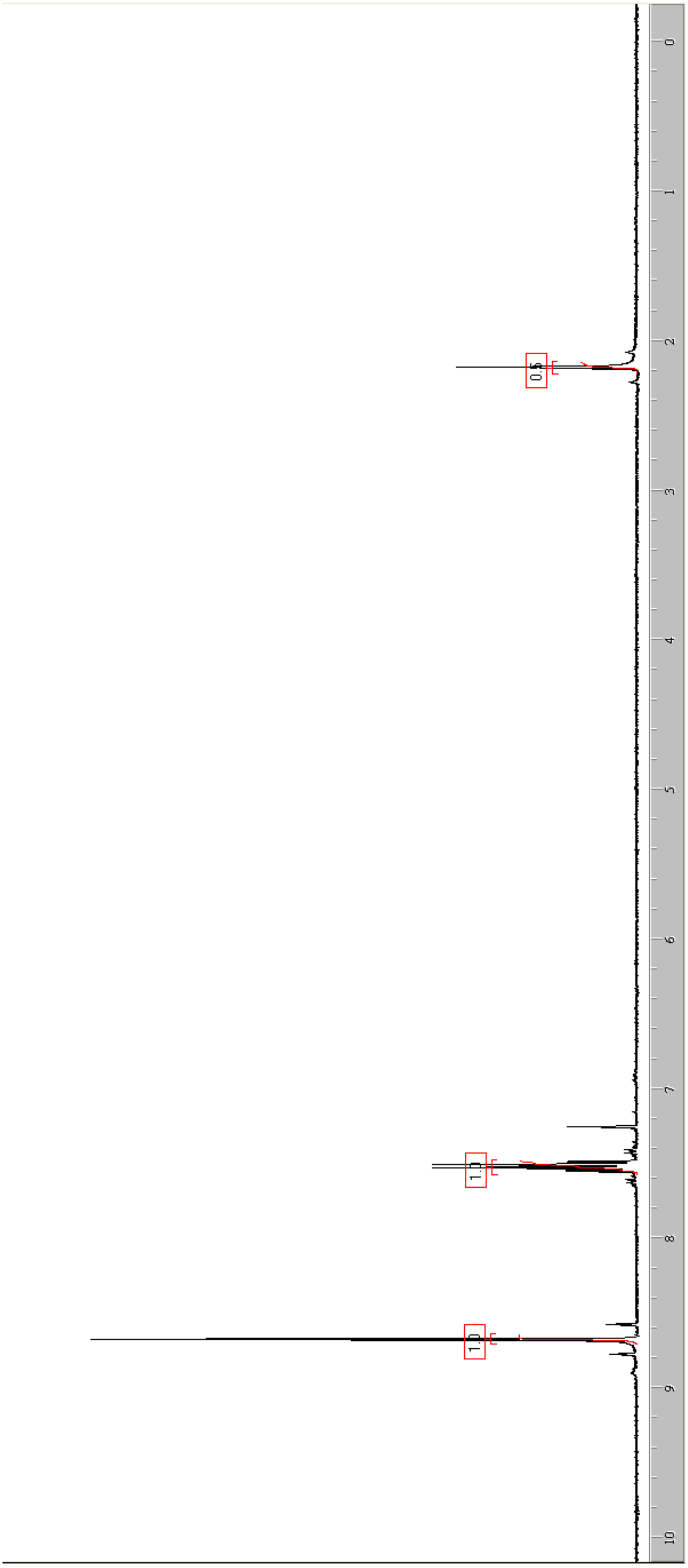

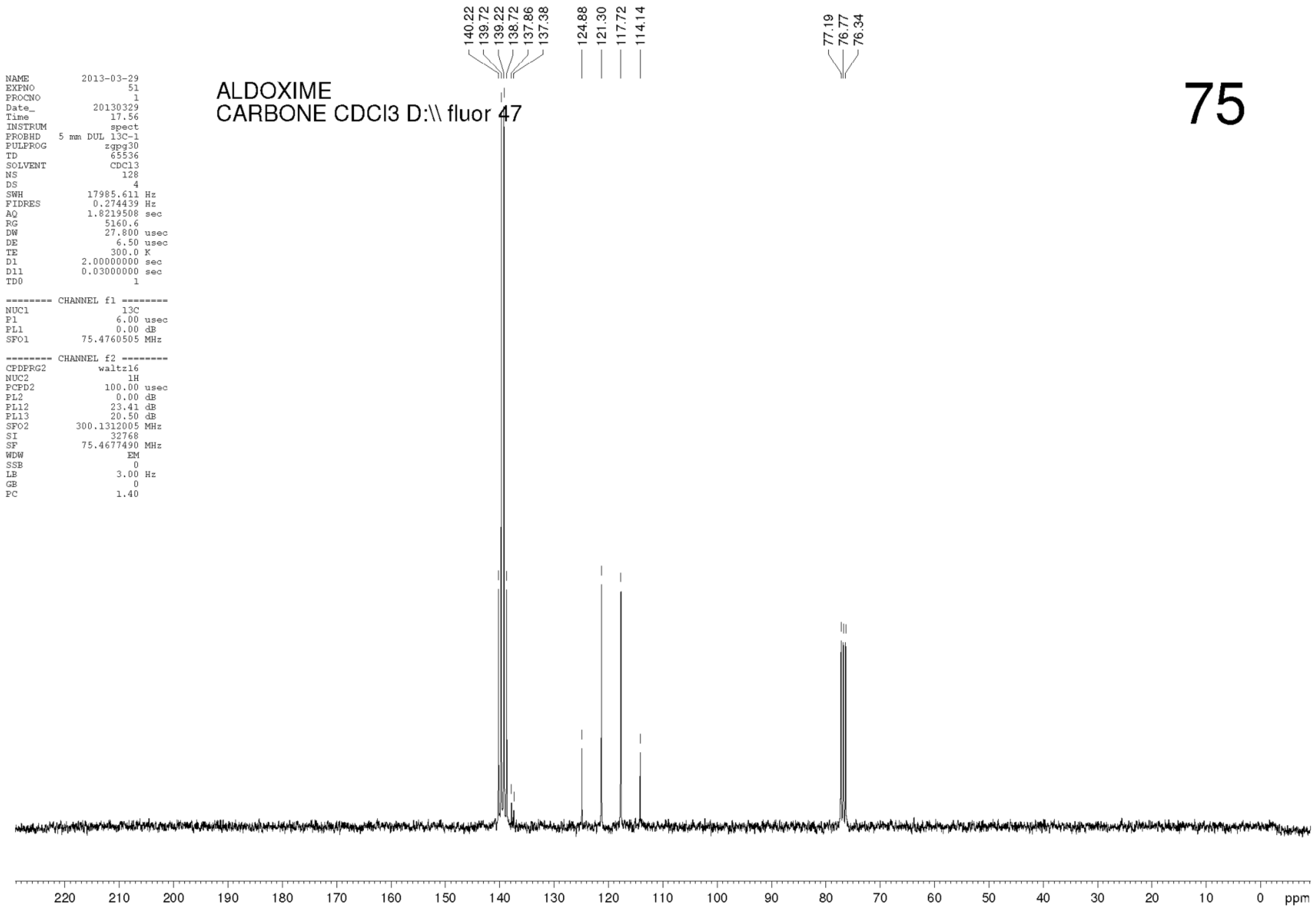
ALDOXIME
CARBONE CDCl3 D:\\ fluor 47
75
140.22
139.72
139.22
138.72
137.86
137.38
124.88
121.30
117.72
114.14
77.19
76.77
76.34
NAME 2013-03-29
EXPNO 51
PROCNO 1
Date_ 20130329
Time 17.56
INSTRUM spect
PROBHD 5 mm DUL 13C-1
PULPROG zgpg30
TD 65536
SOLVENT CDCl3
NS 128
DS 4
SWH 17985.611 Hz
FIDRES 0.274439 Hz
AQ 1.8219508 sec
RG 5160.6
DW 27.800 usec
DE 6.50 usec
TE 300.0 K
D1 2.00000000 sec
D11 0.03000000 sec
TD0 1
======== CHANNEL f1 ========
NUC1 13C
P1 6.00 usec
PL1 0.00 dB
SFO1 75.4760505 MHz
======== CHANNEL f2 ========
CPDPRG2 waltz16
NUC2 1H
PCPD2 100.00 usec
PL2 0.00 dB
PL12 23.41 dB
PL13 20.50 dB
SFO2 300.1312005 MHz
SI 32768
SF 75.4677490 MHz
WDW EM
SSB 0
LB 3.00 Hz
GB 0
PC 1.40
220 210 200 190 180 170 160 150 140 130 120 110 100 90 80 70 60 50 40 30 20 10 0 ppm

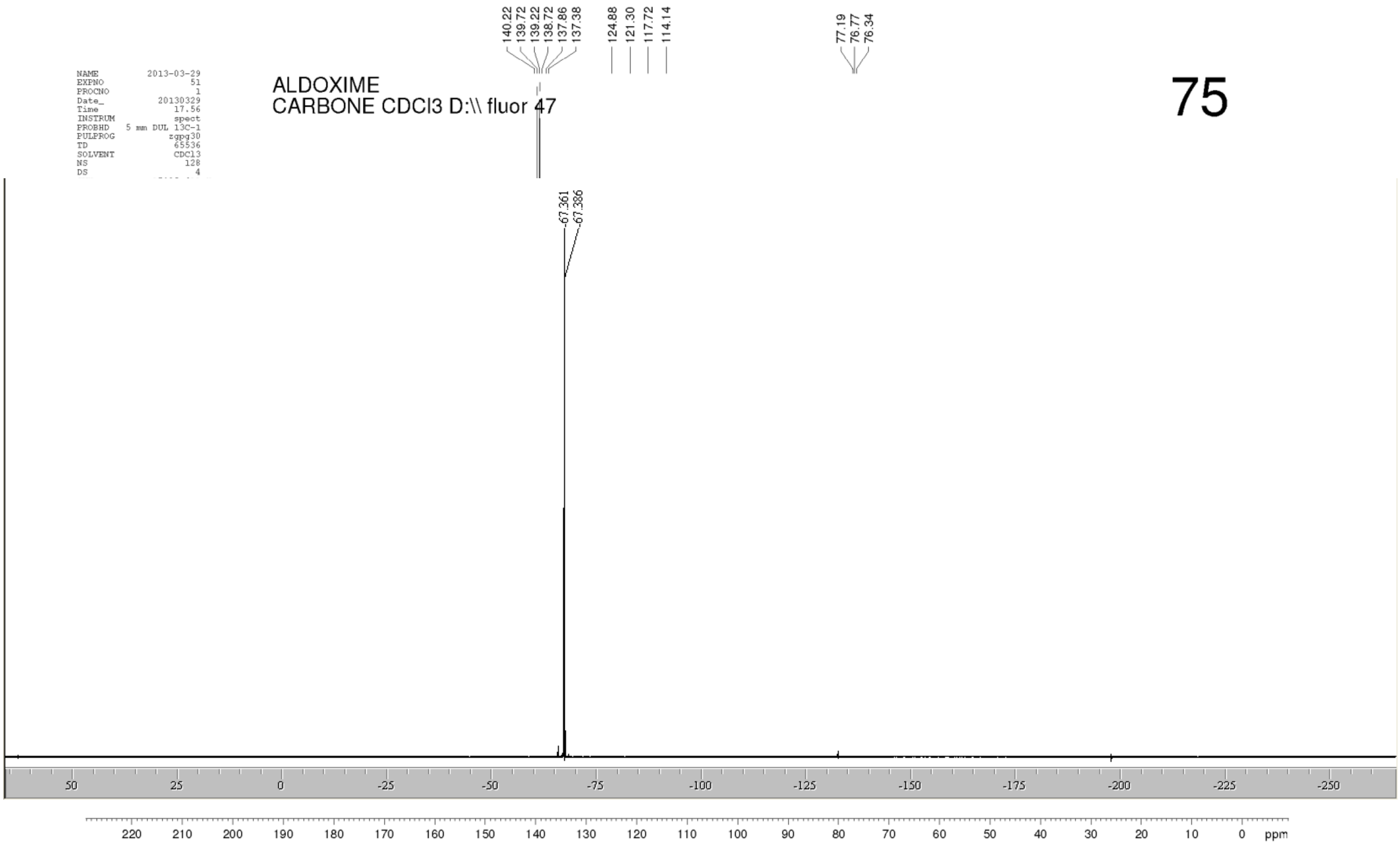
NAME 2013-03-29
EXPNO 51
PROCNO 1
Date_ 20130329
Time 17.56
INSTRUM spect
PROBHD 5 mm DUL 13C-1
PULPROG zgpg30
TD 65536
SOLVENT CDCl3
NS 128
DS 4
ALDOXIME
CARBONE CDCl3 D:\\ fluor 47
75
140.22
139.72
139.22
138.72
137.86
137.38
124.88
121.30
117.72
114.14
77.19
76.77
76.34
-67.361
-67.386
50 25 0 -25 -50 -75 -100 -125 -150 -175 -200 -225 -250
220 210 200 190 180 170 160 150 140 130 120 110 100 90 80 70 60 50 40 30 20 10 0 ppm

Furoxan **6**

^{19}F-^{19}F NOESY

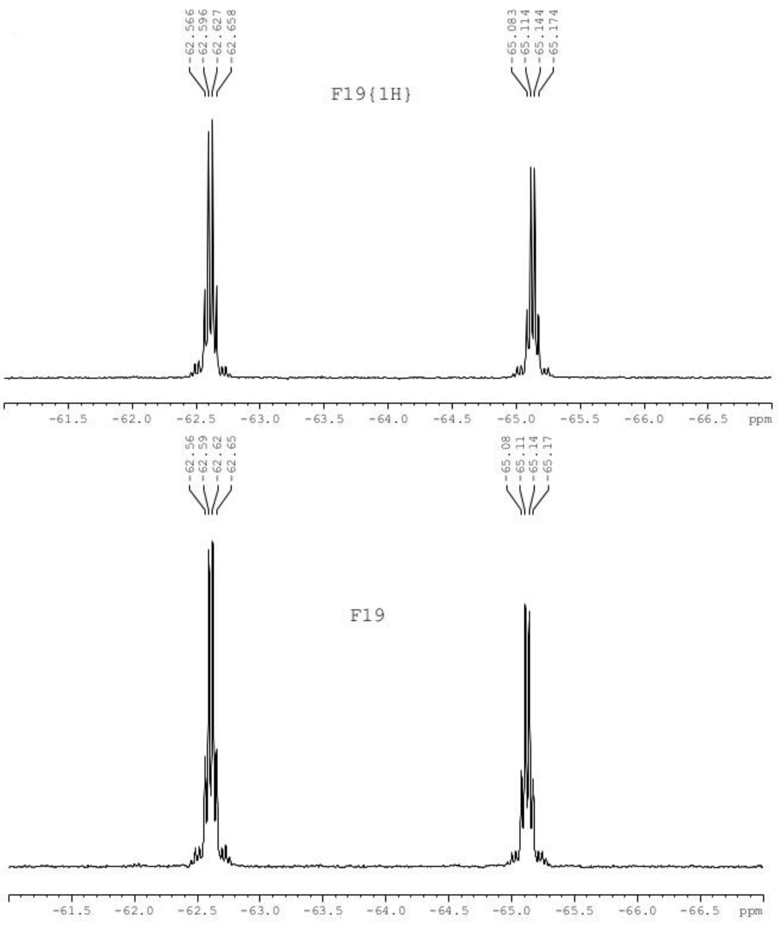

^{19}F-^{19}F COSY

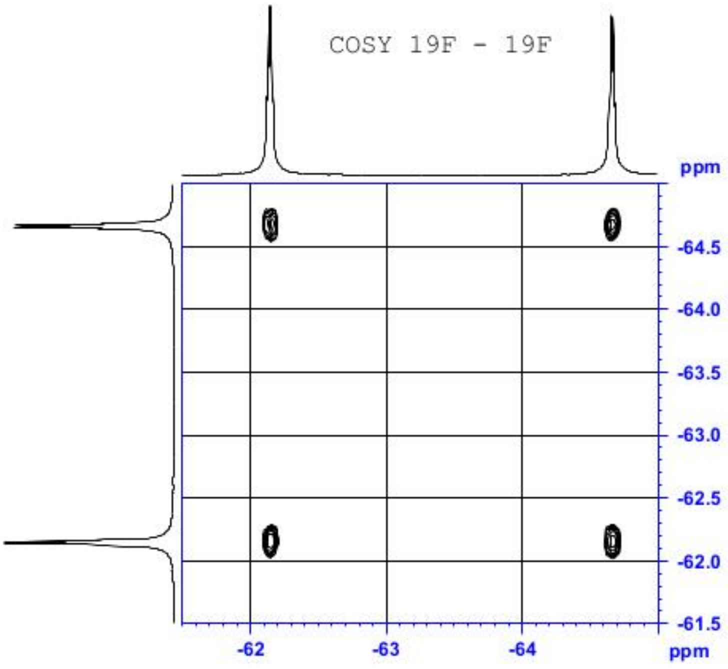

5-Benzyl-3-(trifluoromethyl)-4,5-dihydroisoxazole (**1a**)

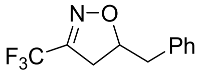

1a

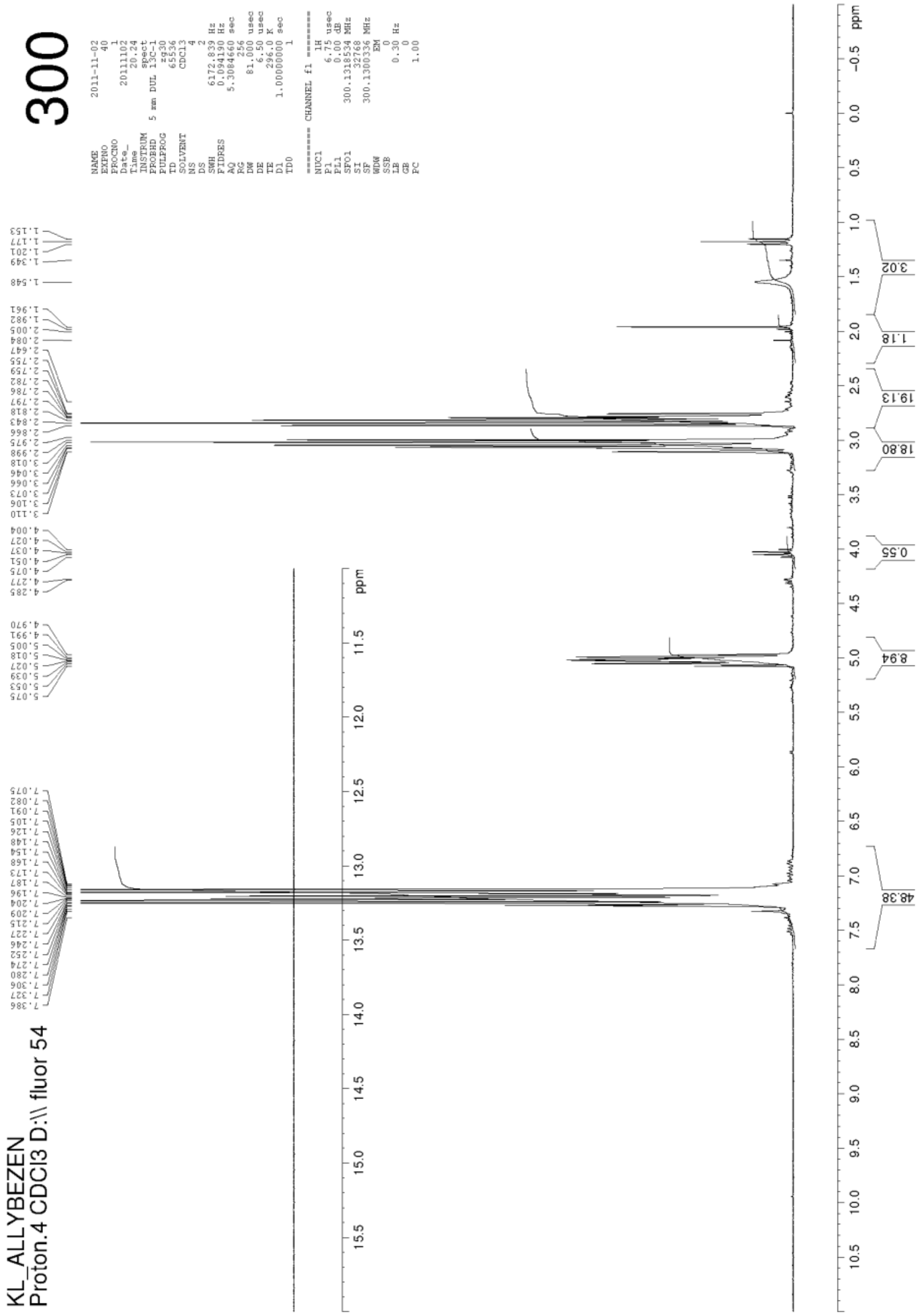

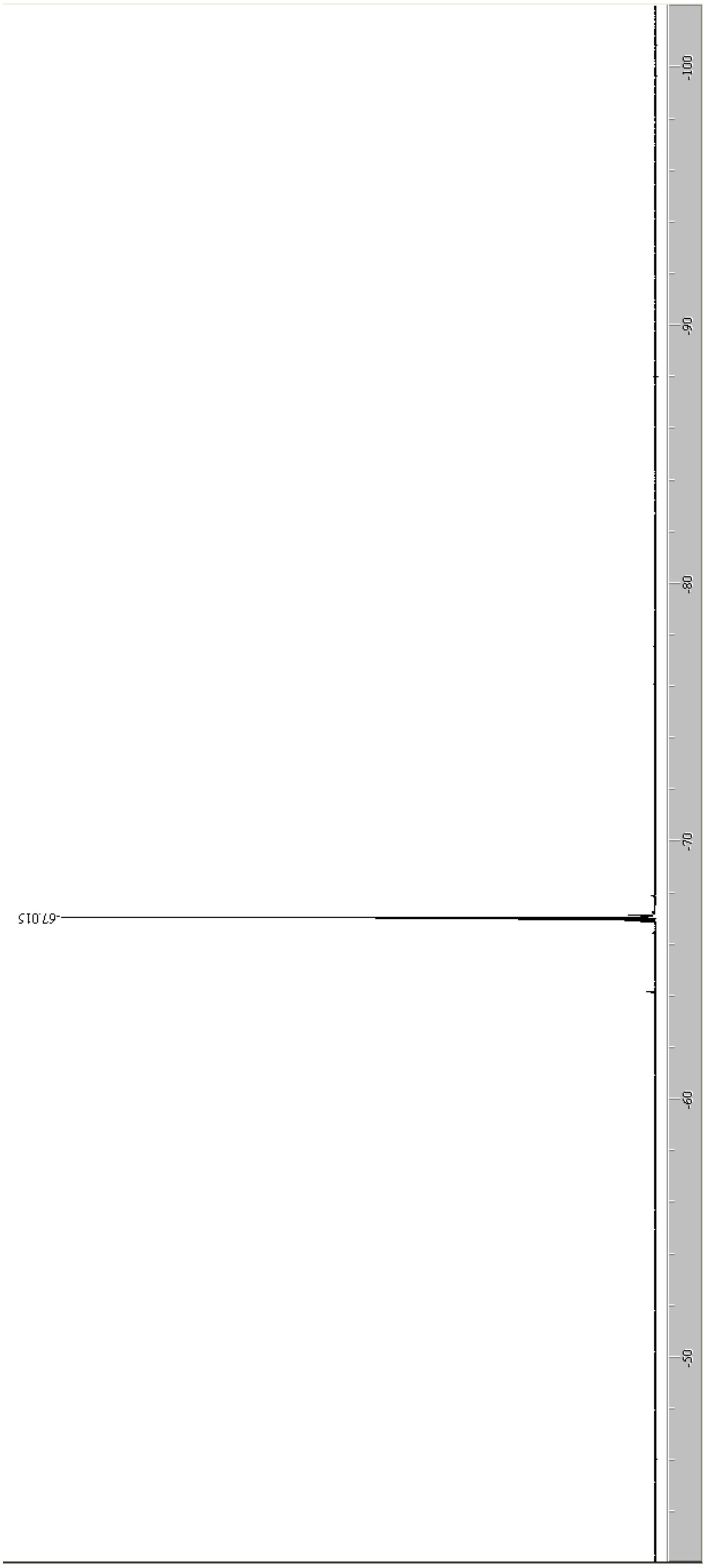
-67.015
-100
-90
-80
-70
-60
-50

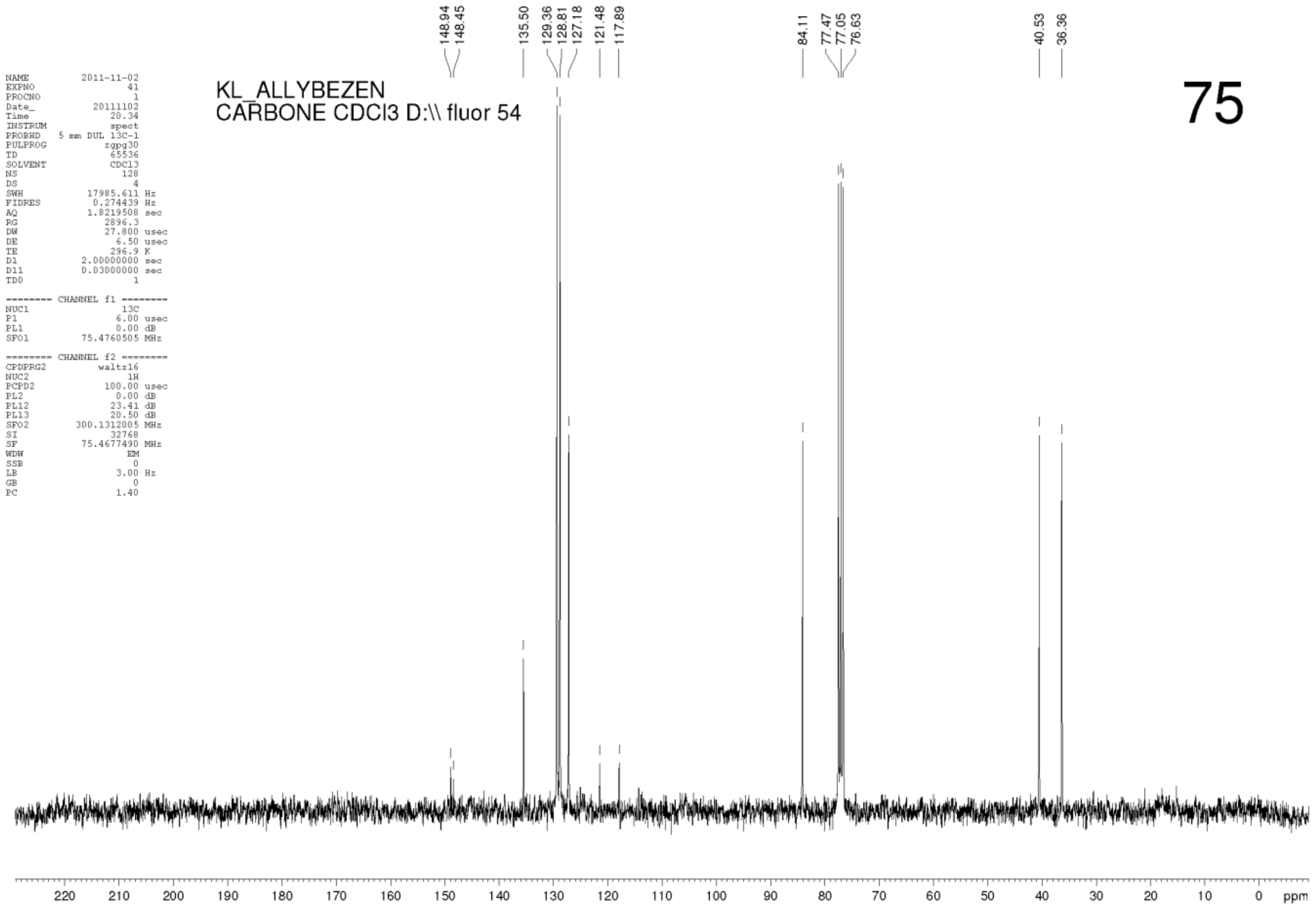
75
KL_ALLYBEZEN
CARBONE CDCl3 D:\\ fluor 54
148.94
148.45
135.50
129.36
128.81
127.18
121.48
117.89
84.11
77.47
77.05
76.63
40.53
36.36
220 210 200 190 180 170 160 150 140 130 120 110 100 90 80 70 60 50 40 30 20 10 0 ppm
NAME 2011-11-02
EXPNO 41
PROCNO 1
Date_ 20111102
Time 20.34
INSTRUM spect
PROBHD 5 mm DUL 13C-1
PULPROG zgpg30
TD 65536
SOLVENT CDCl3
NS 128
DS 4
SWH 17985.611 Hz
FIDRES 0.274439 Hz
AQ 1.8219508 sec
RG 2896.3
DW 27.800 usec
DE 6.50 usec
TE 296.9 K
D1 2.00000000 sec
D11 0.03000000 sec
TD0 1
======== CHANNEL f1 ========
NUC1 13C
P1 6.00 usec
PL1 0.00 dB
SFO1 75.4760505 MHz
======== CHANNEL f2 ========
CPDPRG2 waltz16
NUC2 1H
PCPD2 100.00 usec
PL2 0.00 dB
PL12 23.41 dB
PL13 20.50 dB
SFO2 300.1312005 MHz
SI 32768
SF 75.4677490 MHz
WDW EM
SSB 0
LB 3.00 Hz
GB 0
PC 1.40

Benzyl (3-(trifluoromethyl)-4,5-dihydroisoxazol-5-yl)methylcarbamate (**1b**)

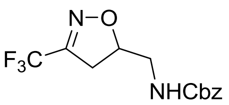

1b

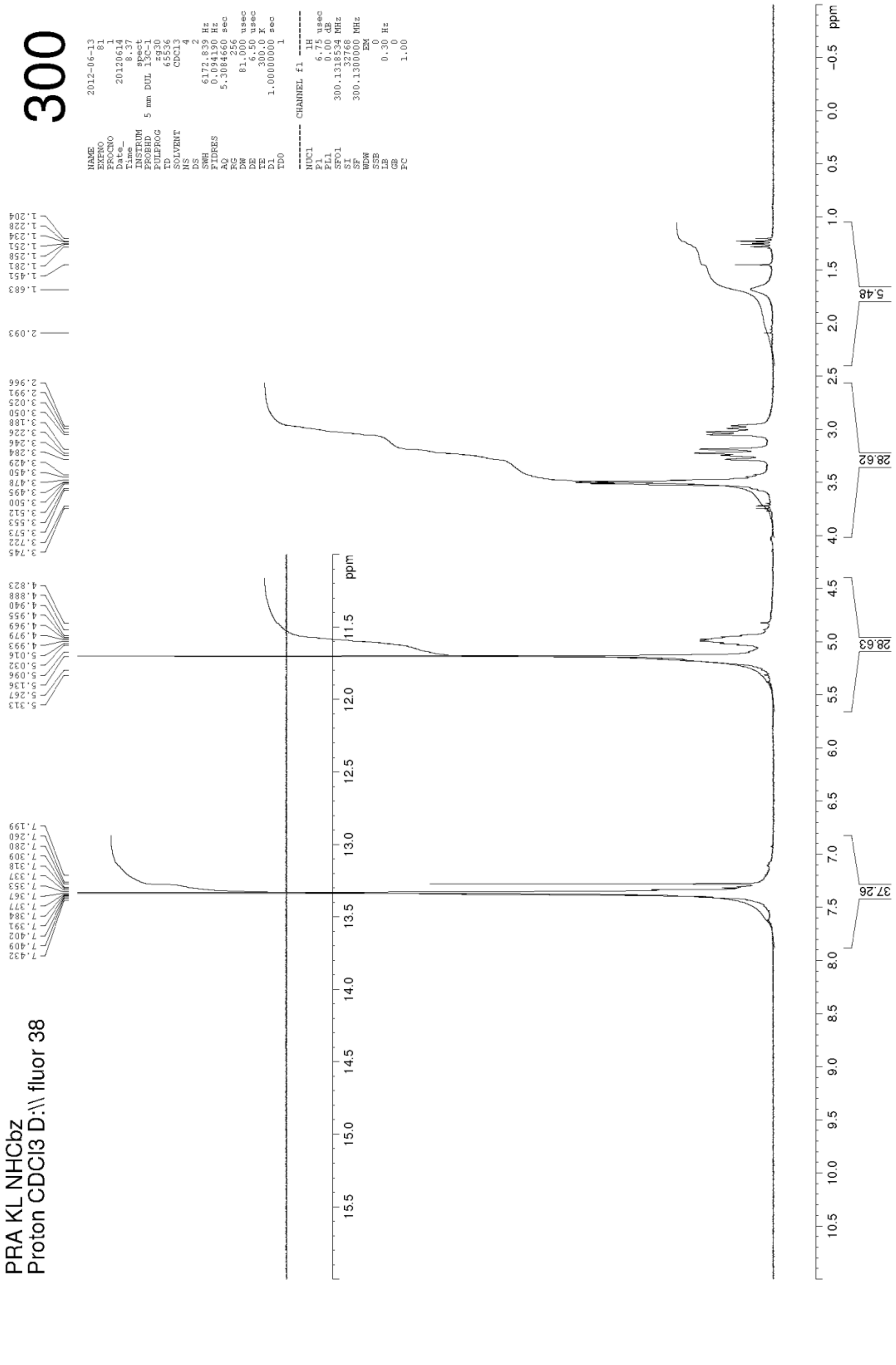

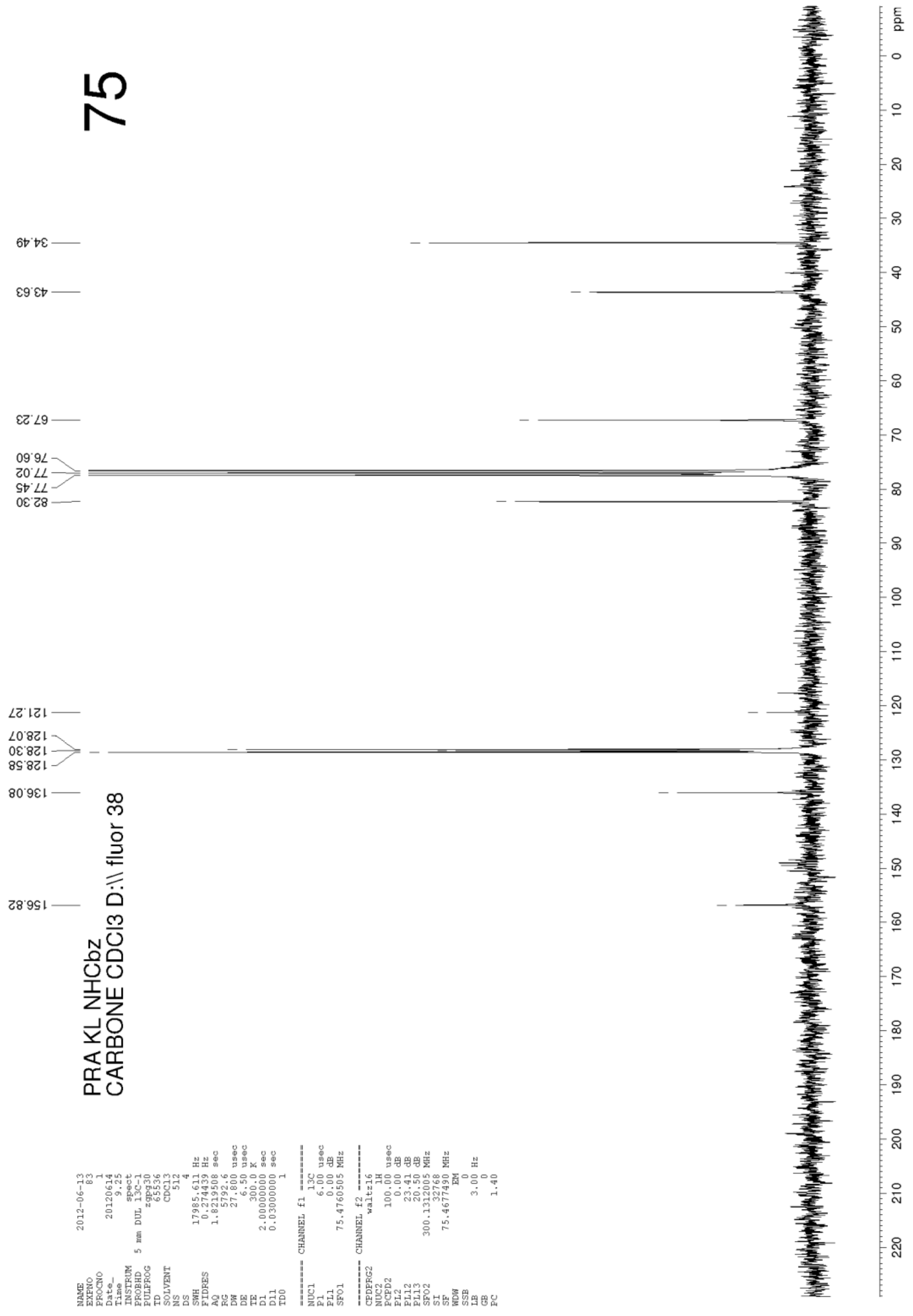
75
PRA KL NHCbz
CARBONE CDCl3 D:\\ fluor 38
156.82
136.08
128.58
128.30
128.07
121.27
82.30
77.45
77.02
76.60
67.23
43.63
34.49
ppm
NAME 2012-06-13
EXPNO 83
PROCNO 1
Date_ 20120614
Time 9.25
INSTRUM spect
PROBHD 5 mm DUL 13C-1
PULPROG zgpg30
TD 65536
SOLVENT CDCl3
NS 512
DS 4
SWH 17985.611 Hz
FIDRES 0.274439 Hz
AQ 1.8219508 sec
RG 5792.6
DW 27.800 usec
DE 6.50 usec
TE 300.0 K
D1 2.00000000 sec
D11 0.03000000 sec
TD0 1
======== CHANNEL f1 ========
NUC1 13C
P1 6.00 usec
PL1 0.00 dB
SFO1 75.4760505 MHz
======== CHANNEL f2 ========
CPDPRG2 waltz16
NUC2 1H
PCPD2 100.00 usec
PL2 0.00 dB
PL12 23.41 dB
PL13 20.50 dB
SFO2 300.1312005 MHz
SI 32768
SF 75.4677490 MHz
WDW EM
SSB 0
LB 3.00 Hz
GB 0
PC 1.40

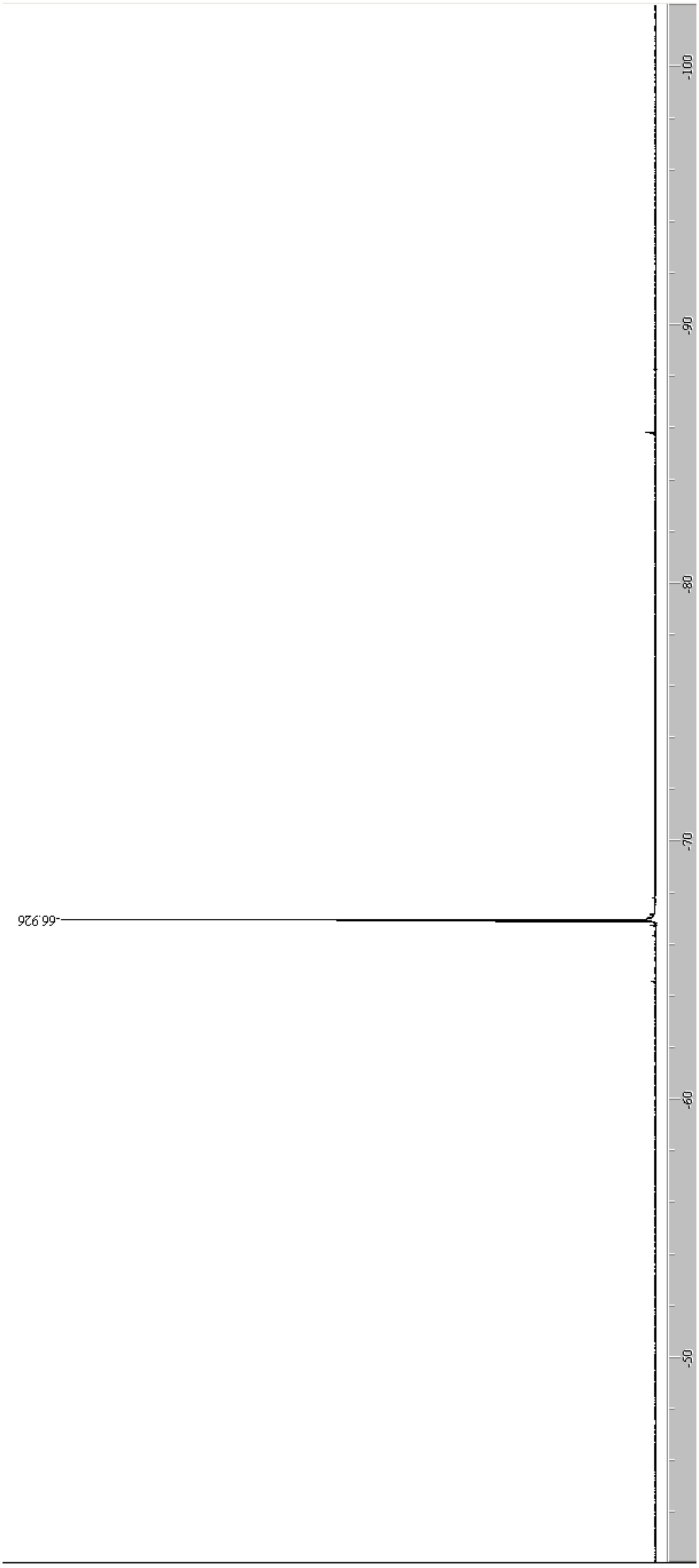
-66.926
-100
-90
-80
-70
-60
-50

tert-Butyl (3-(trifluoromethyl)-4,5-dihydroisoxazol-5-yl)methylcarbamate (**1c**)

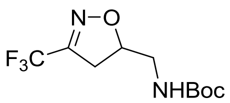

1c

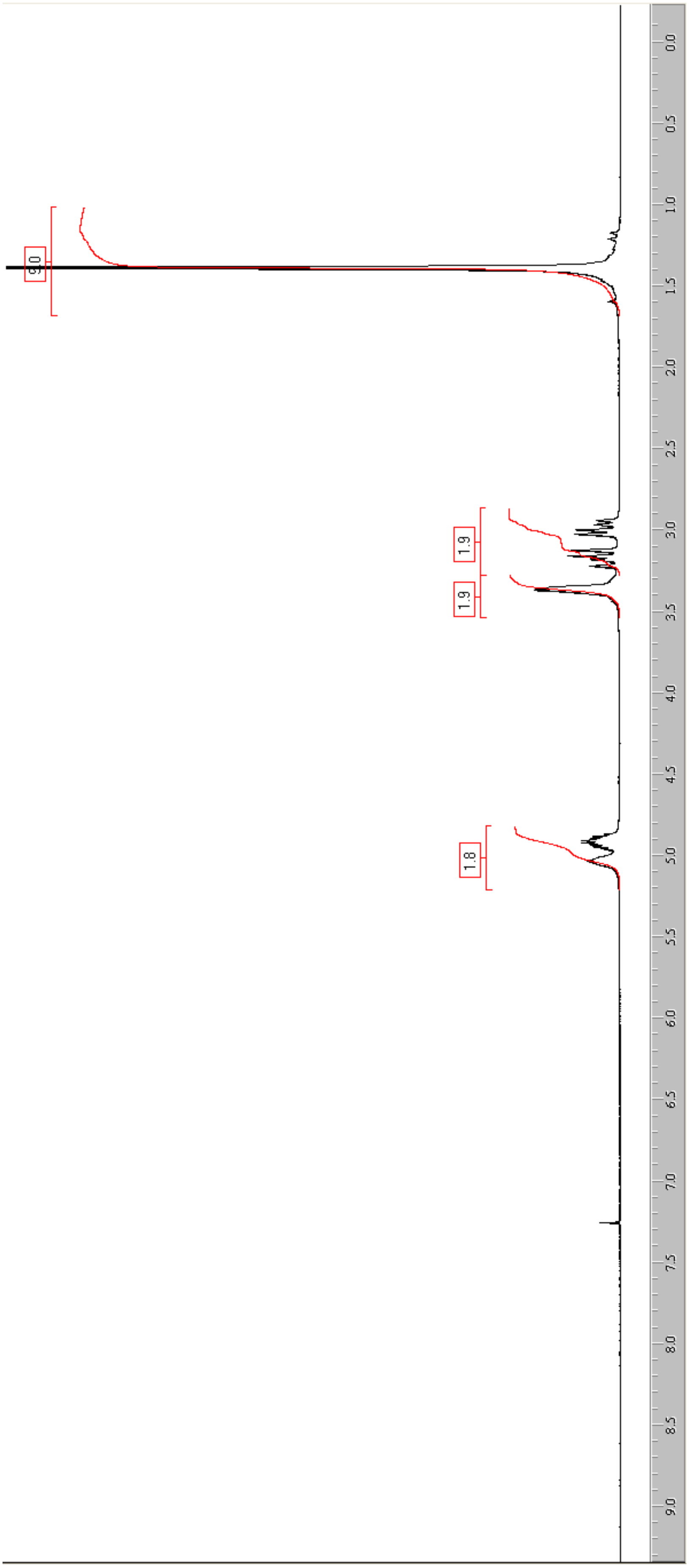

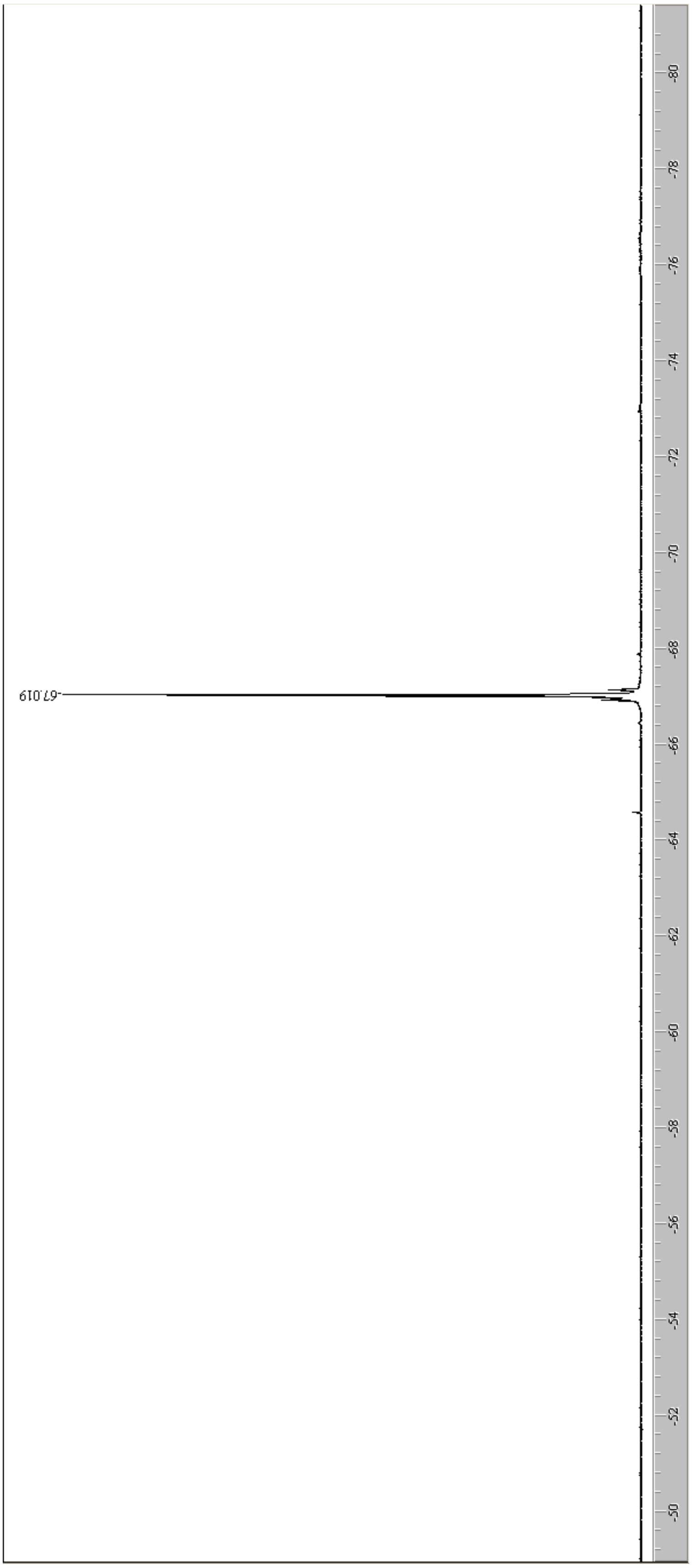
-67.019
-80
-78
-76
-74
-72
-70
-68
-66
-64
-62
-60
-58
-56
-54
-52
-50

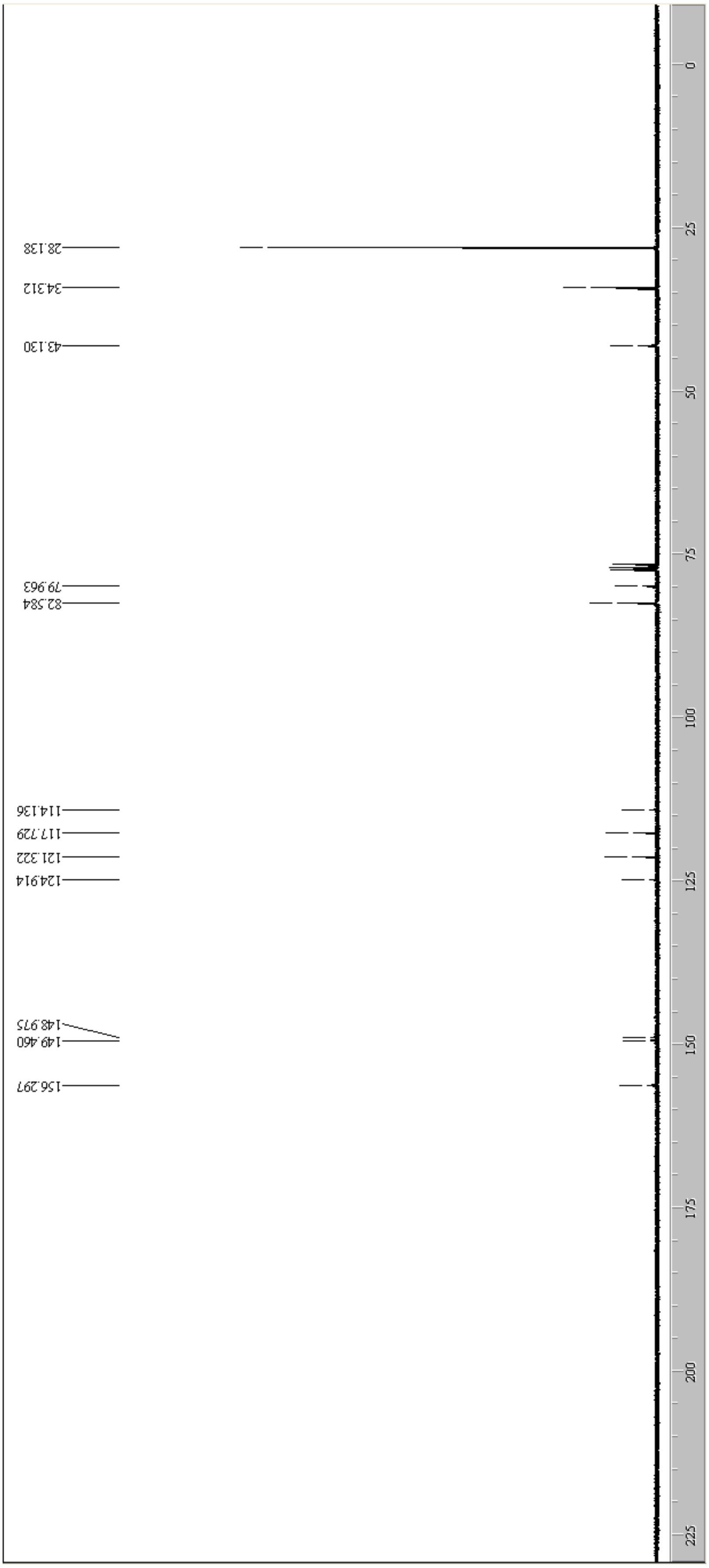
28.138
34.312
43.130
79.963
82.584
114.136
117.729
121.322
124.914
148.975
149.460
156.297
0
25
50
75
100
125
150
175
200
225

Ethyl 9-(3-(trifluoromethyl)-4,5-dihydroisoxazol-5-yl)nonanoate (**1d**)

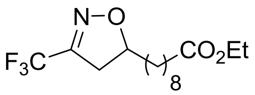

1d

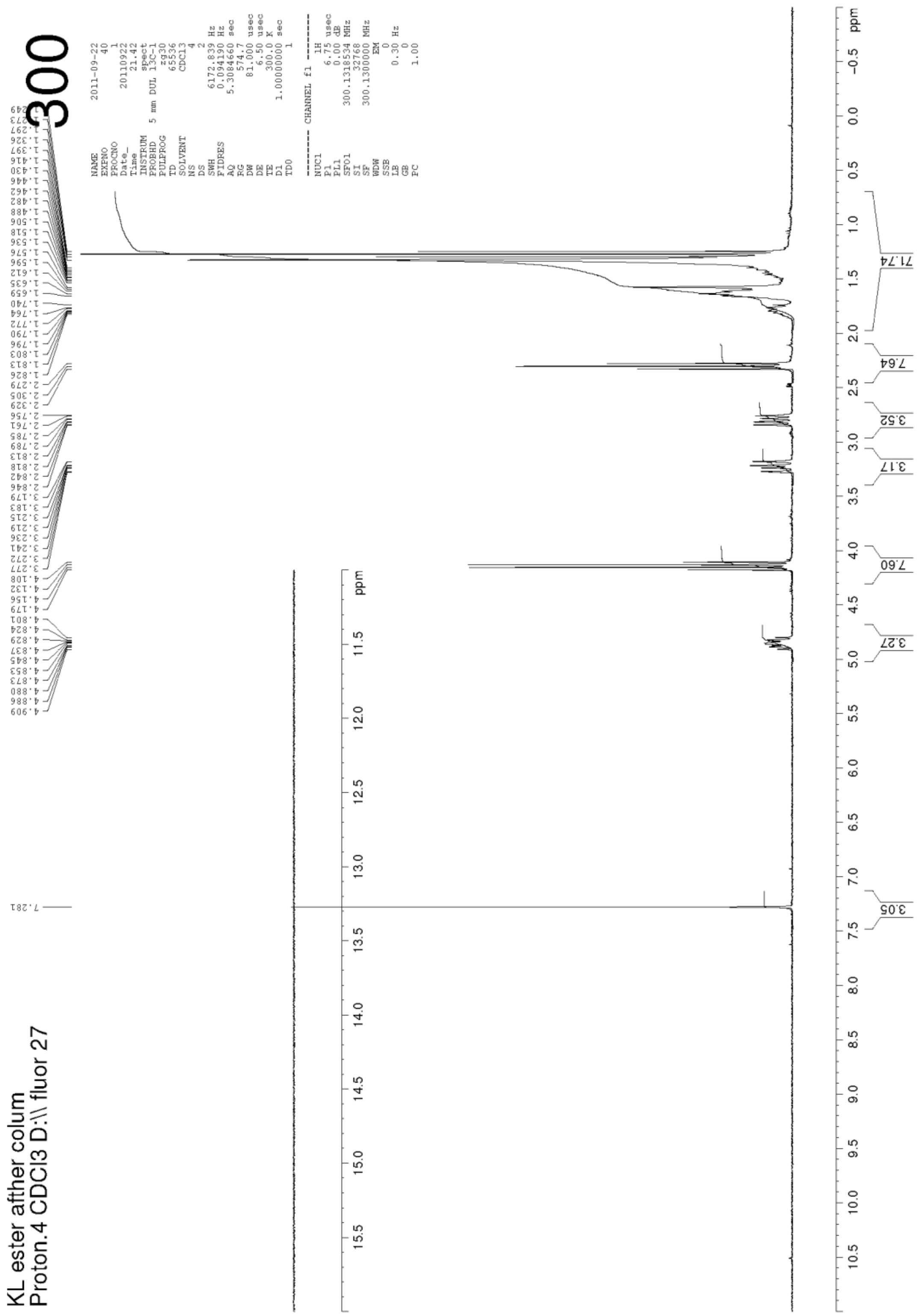

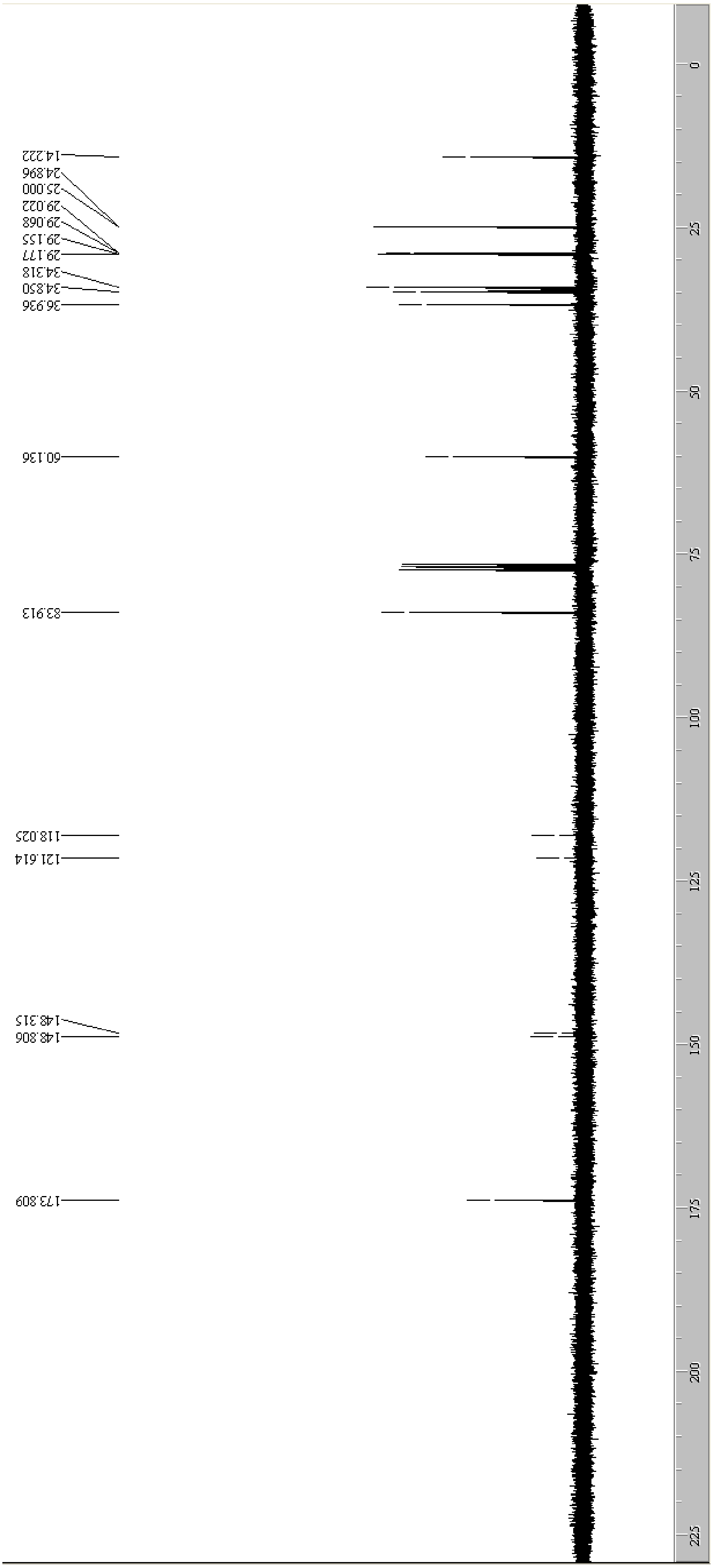
14.222
24.896
25.000
29.022
29.068
29.155
29.177
34.318
34.850
36.936
60.136
83.913
118.025
121.614
148.315
148.806
173.809
0
25
50
75
100
125
150
175
200
225

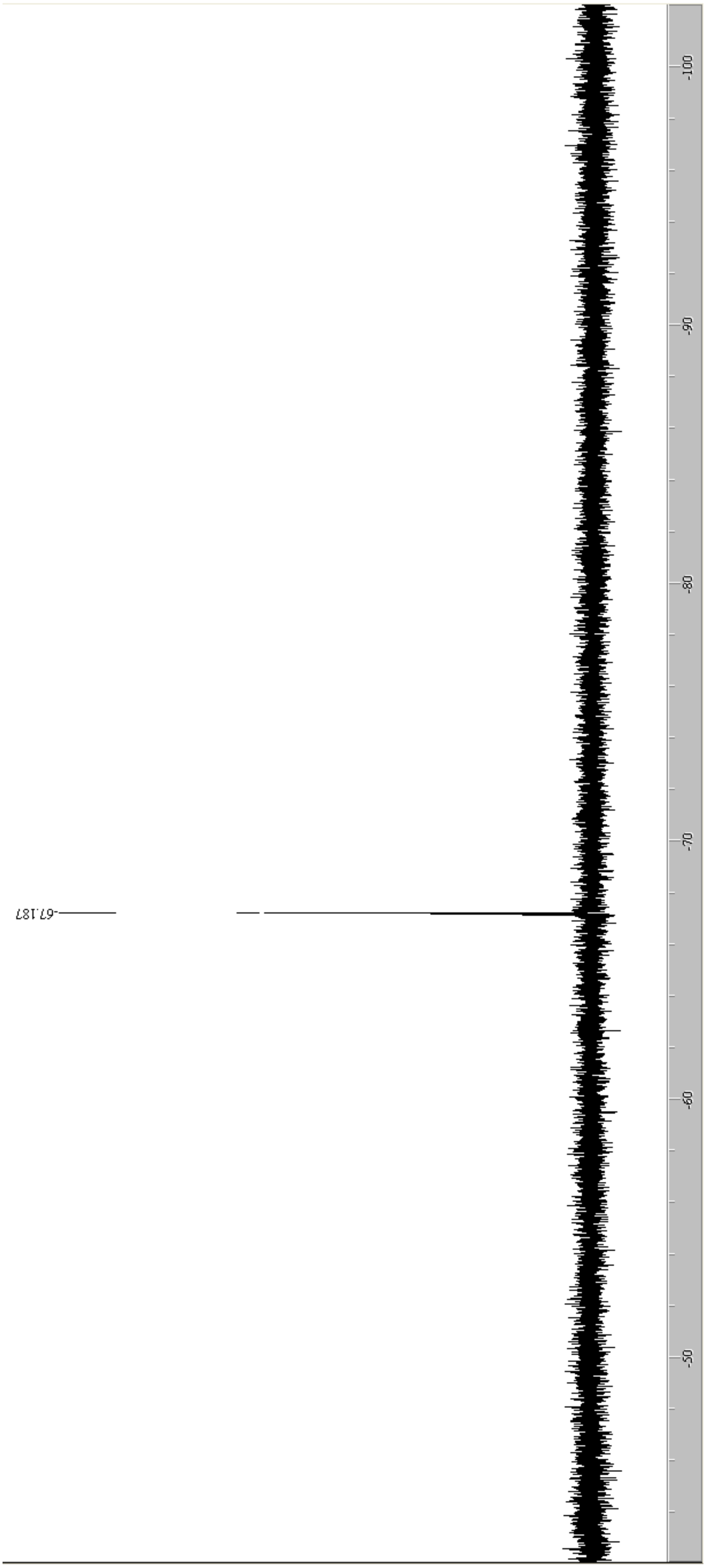
-67.187
-100
-90
-80
-70
-60
-50

9-(3-(Trifluoromethyl)-4,5-dihydroisoxazol-5-yl)nonanoic acid (**1e**)

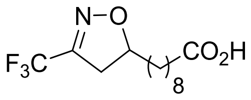

1e

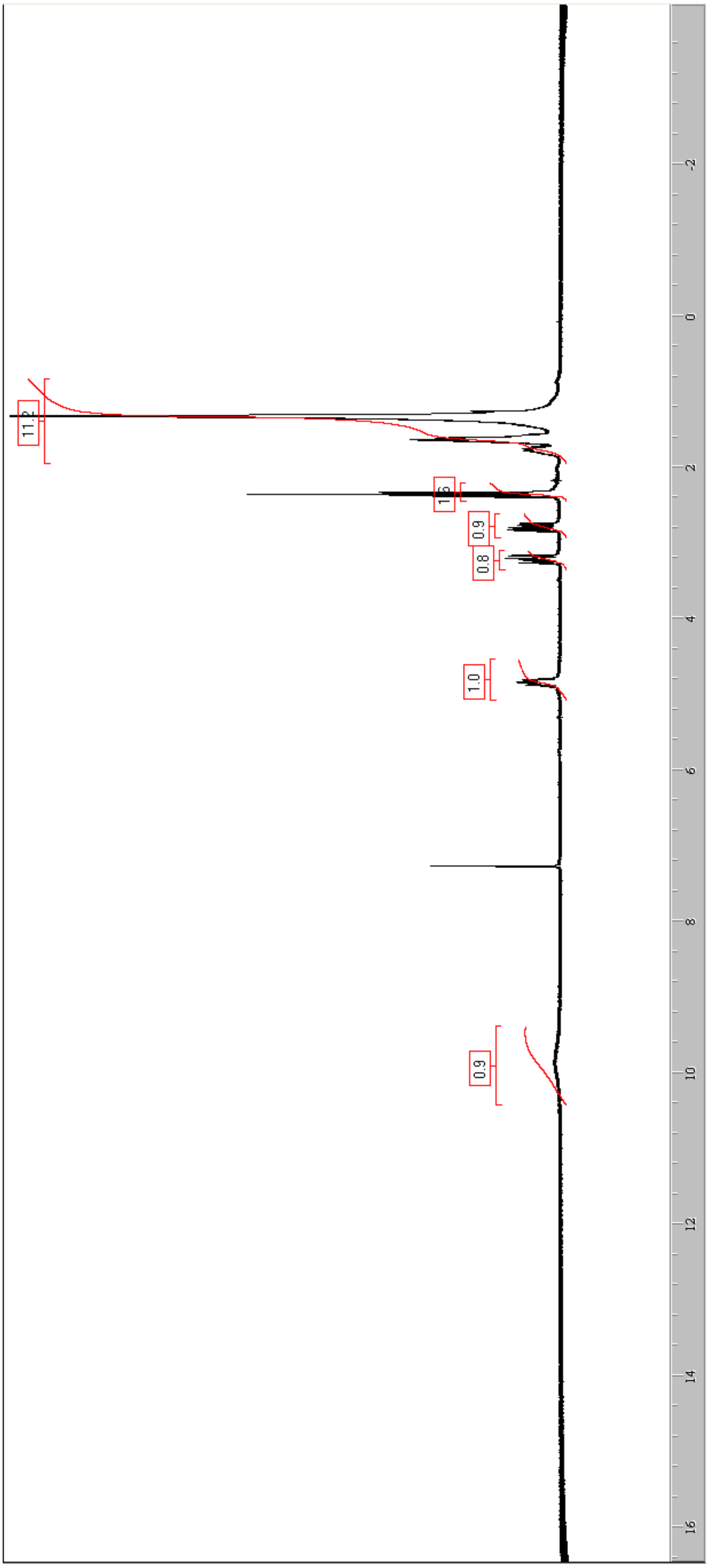

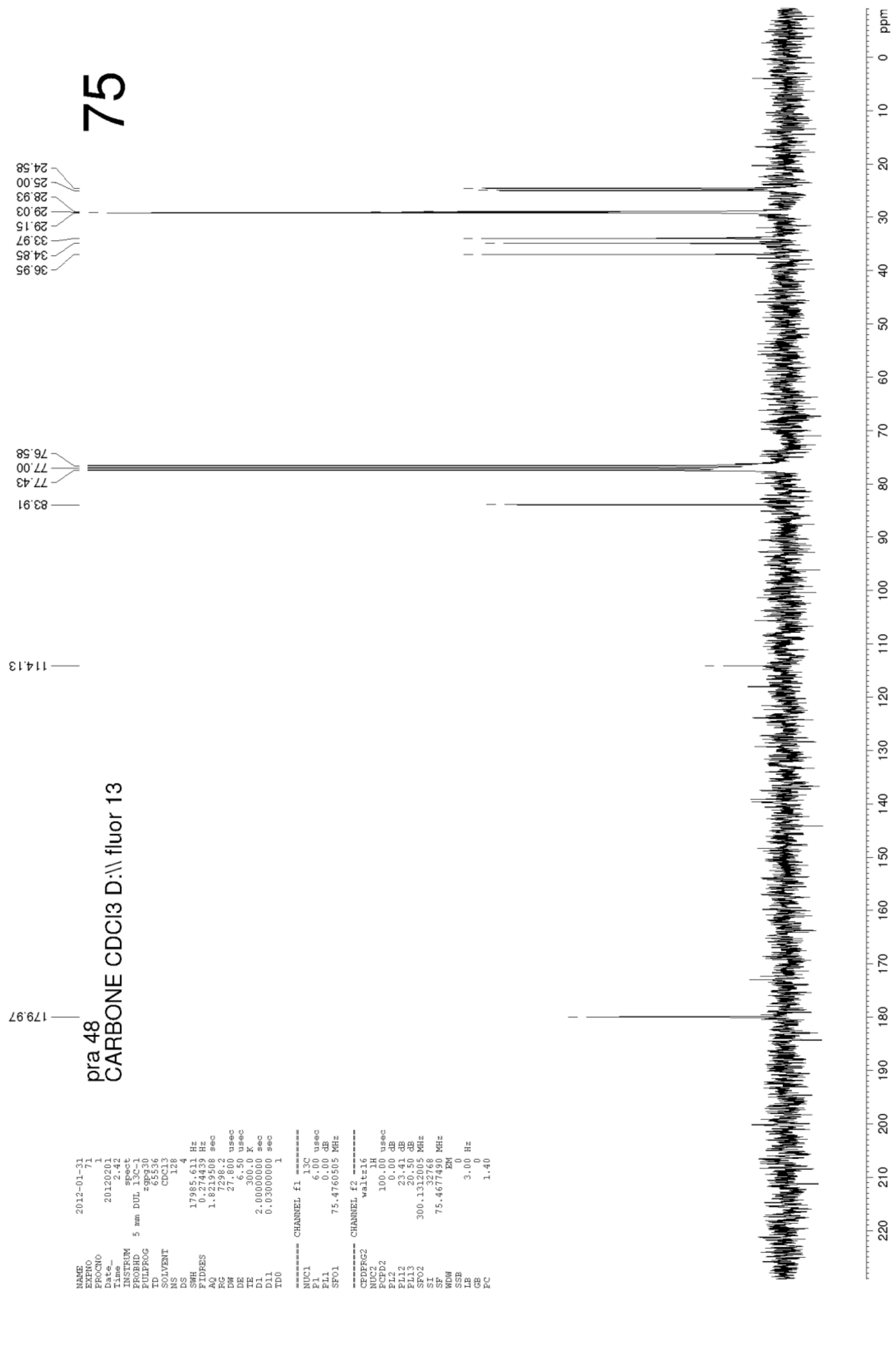
75
pra 48
CARBONE CDCl3 D:\\ fluor 13
179.97
114.13
83.91
77.43
77.00
76.58
36.95
34.85
33.97
29.15
29.03
28.93
25.00
24.58
ppm
NAME 2012-01-31
EXPNO 71
PROCNO 1
Date_ 20120201
Time 2.42
INSTRUM spect
PROBHD 5 mm DUL 13C-1
PULPROG zgpg30
TD 65536
SOLVENT CDCl3
NS 128
DS 4
SWH 17985.611 Hz
FIDRES 0.274439 Hz
AQ 1.8219508 sec
RG 7298.2
DW 27.800 usec
DE 6.50 usec
TE 300.0 K
D1 2.00000000 sec
D11 0.03000000 sec
TD0 1
======== CHANNEL f1 ========
NUC1 13C
P1 6.00 usec
PL1 0.00 dB
SFO1 75.4760505 MHz
======== CHANNEL f2 ========
CPDPRG2 waltz16
NUC2 1H
PCPD2 100.00 usec
PL2 0.00 dB
PL12 23.41 dB
PL13 20.50 dB
SFO2 300.1312005 MHz
SI 32768
SF 75.4677490 MHz
WDW EM
SSB 0
LB 3.00 Hz
GB 0
PC 1.40

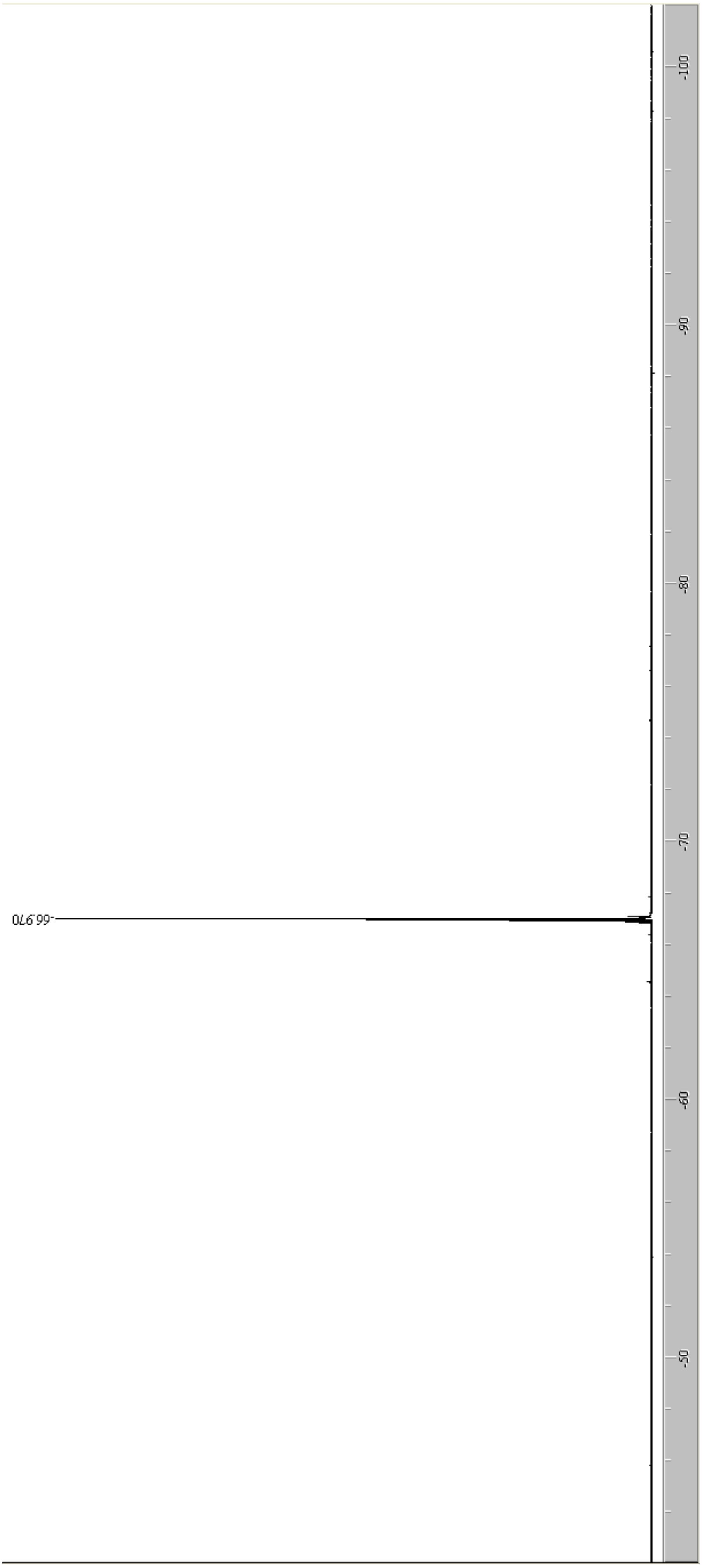
-66.970
-100
-90
-80
-70
-60
-50

5-(4-Bromophenyl)-3-(trifluoromethyl)-4,5-dihydroisoxazole (**1f**)

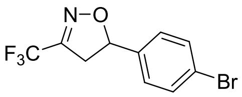

1f

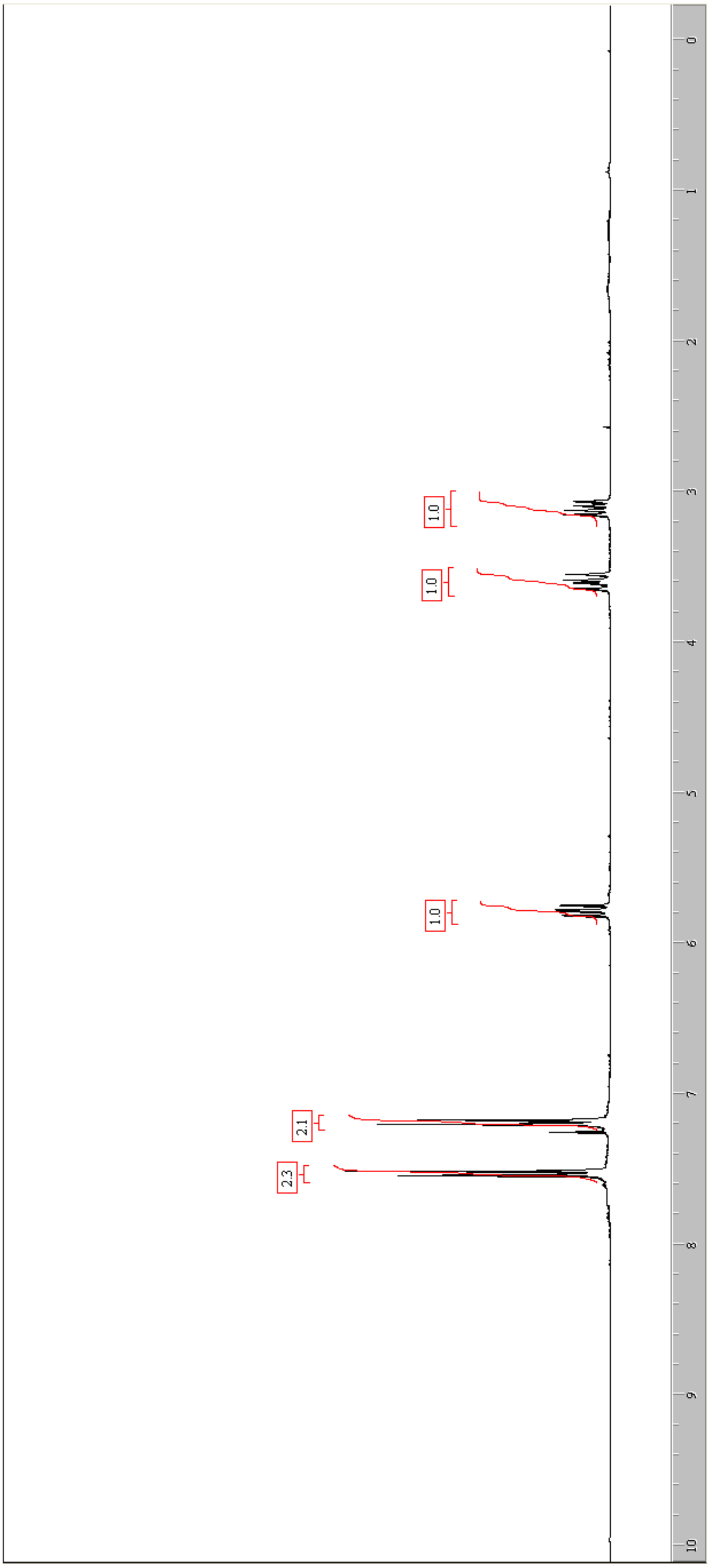

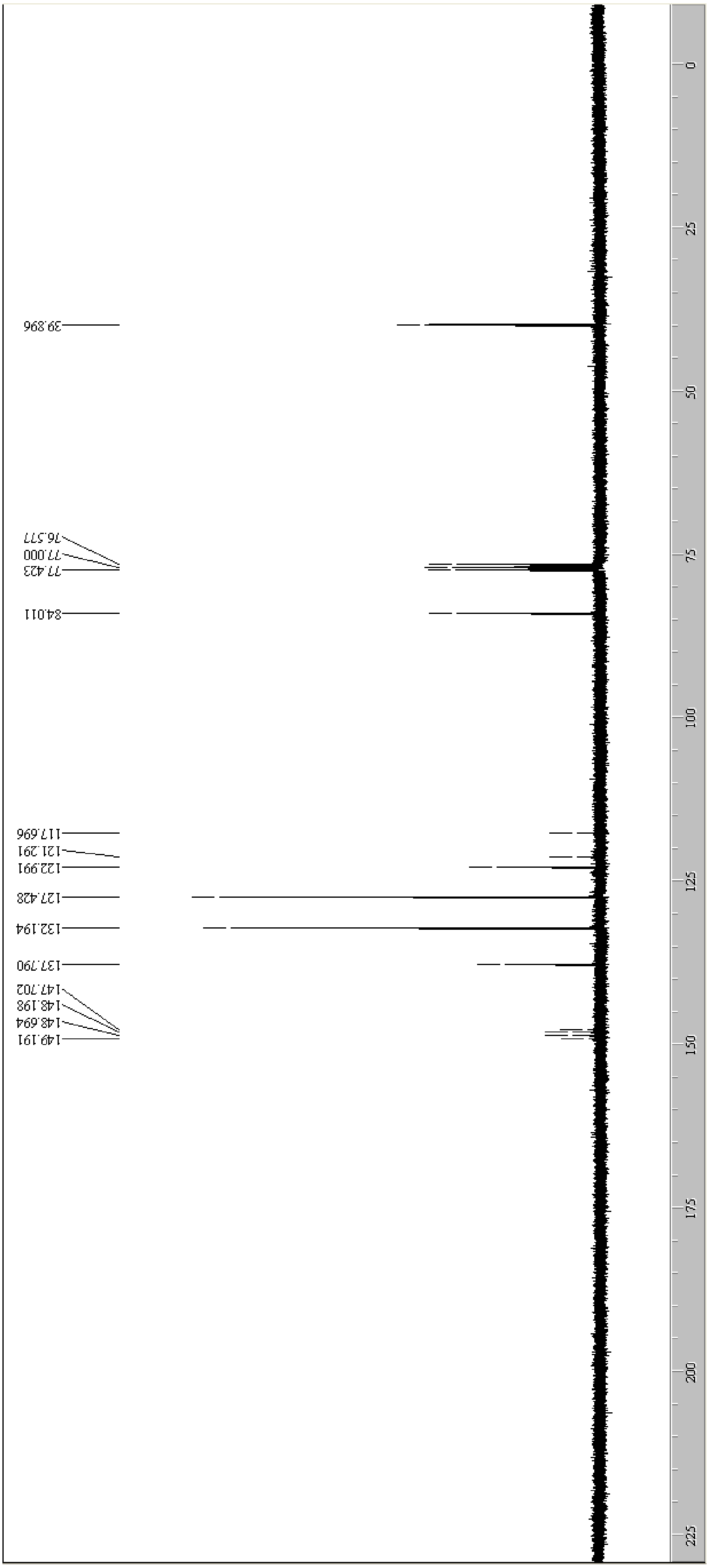
39.896
76.577
77.000
77.423
84.011
117.696
121.291
122.991
127.428
132.194
137.790
147.702
148.198
148.694
149.191
0
25
50
75
100
125
150
175
200
225

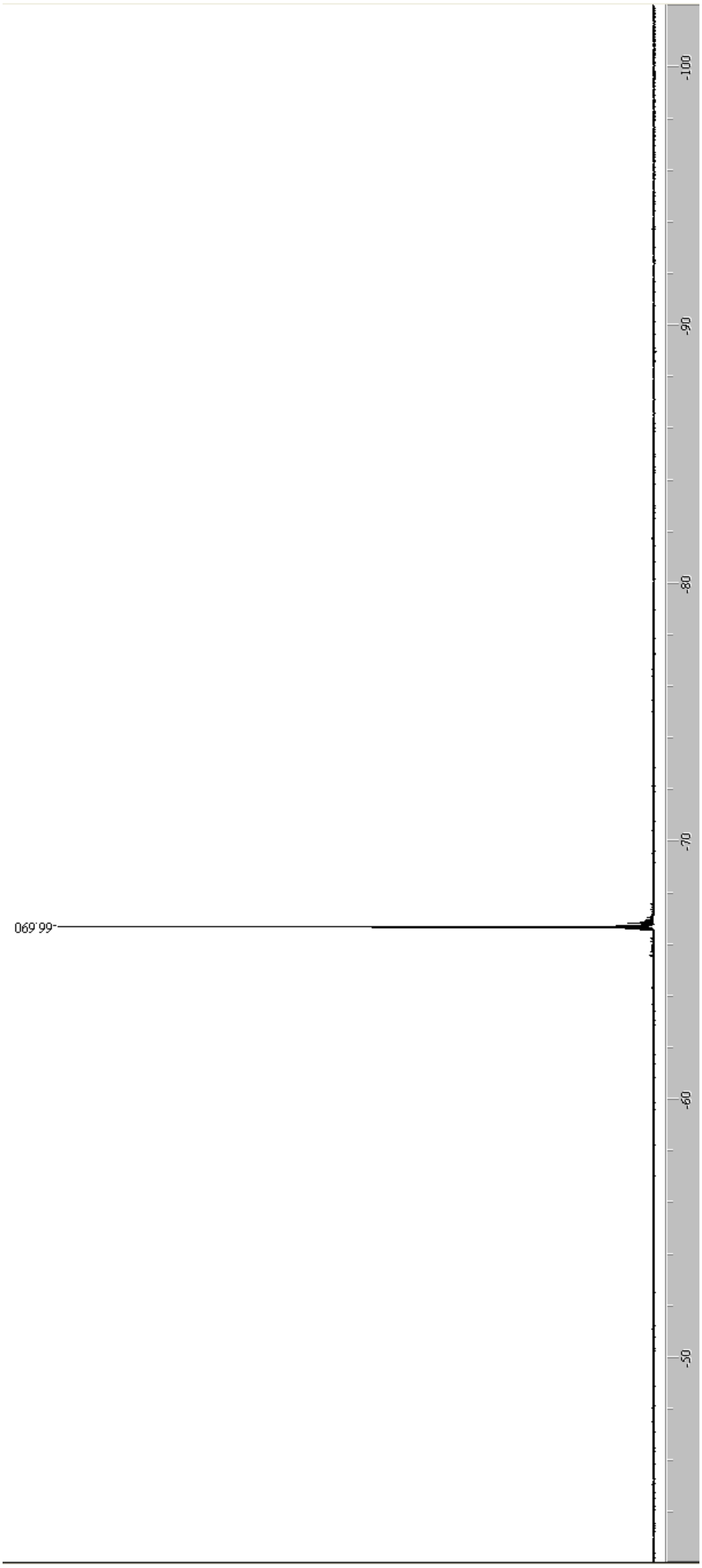
-66.690
-100
-90
-80
-70
-60
-50

3-(Trifluoromethyl)-5-((trimethylsilyl)methyl)-4,5-dihydroisoxazole (**1g**)

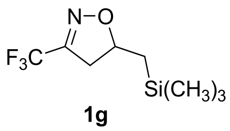

1g

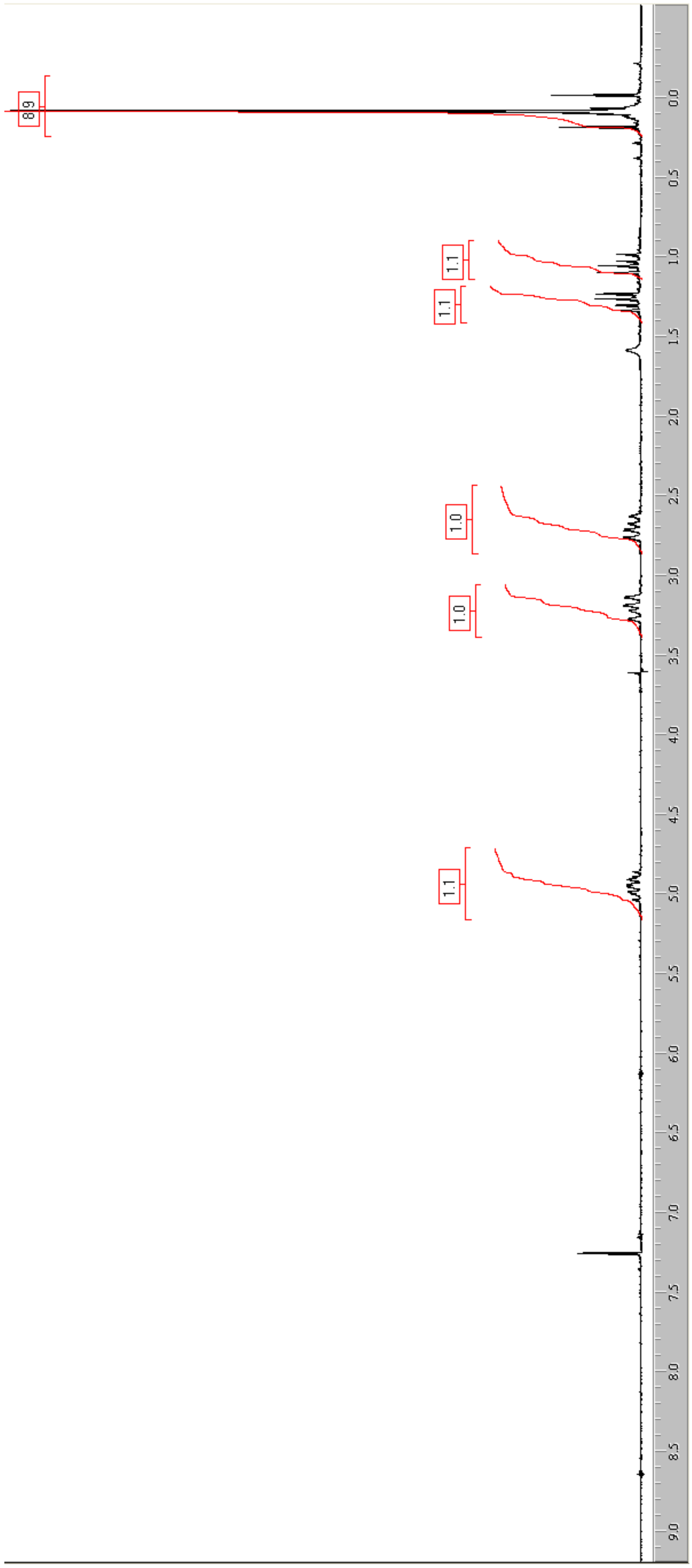

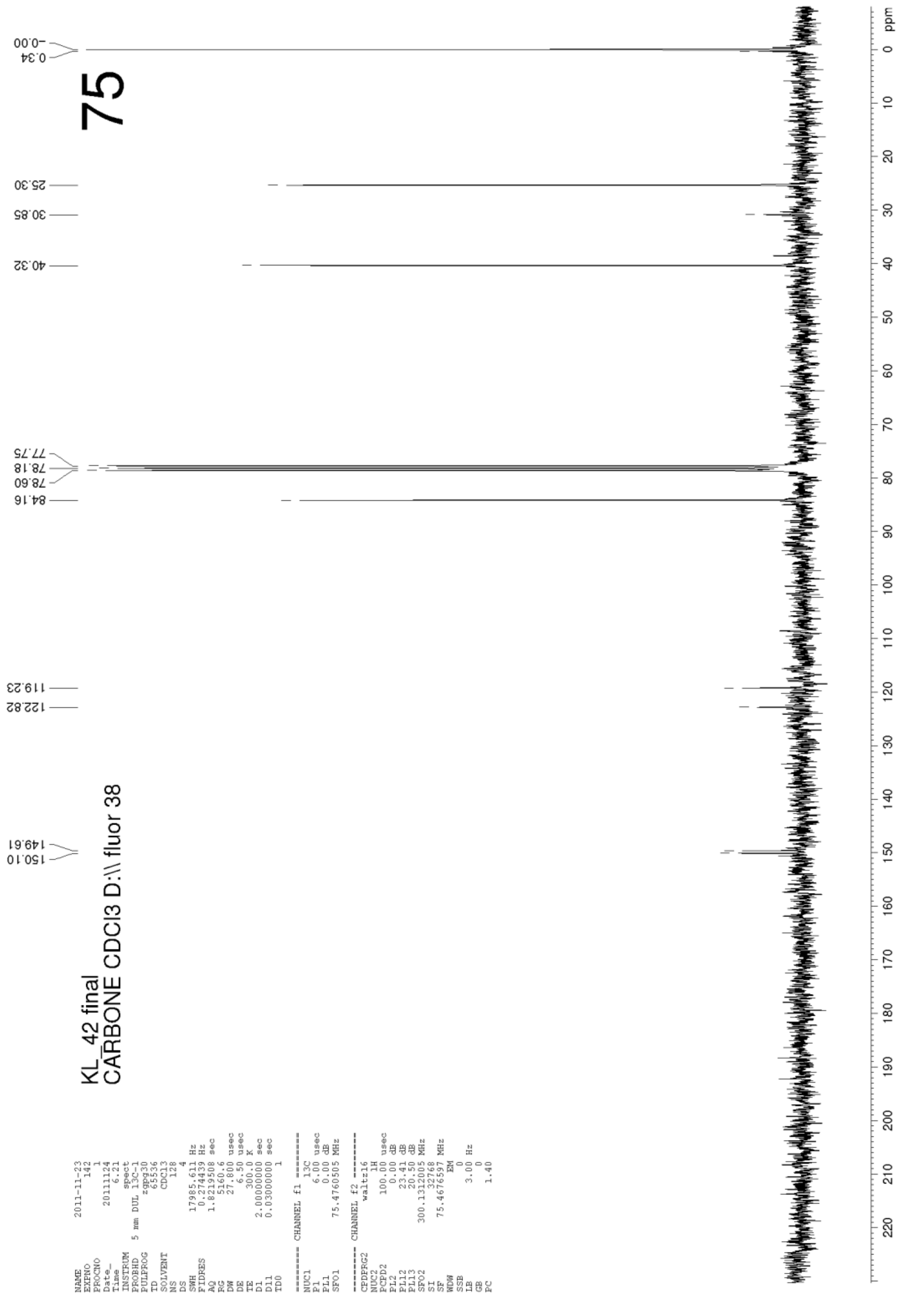

75
KL_42 final
CARBONE CDCl3 D:\\ fluor 38
-0.00
0.34
25.30
30.85
40.32
77.75
78.18
78.60
84.16
119.23
122.82
149.61
150.10
ppm
NAME 2011-11-23
EXPNO 142
PROCNO 1
Date_ 20111124
Time 6.21
INSTRUM spect
PROBHD 5 mm DUL 13C-1
PULPROG zgpg30
TD 65536
SOLVENT CDCl3
NS 128
DS 4
SWH 17985.611 Hz
FIDRES 0.274439 Hz
AQ 1.8219508 sec
RG 5160.6
DW 27.800 usec
DE 6.50 usec
TE 300.0 K
D1 2.00000000 sec
D11 0.03000000 sec
TD0 1
======== CHANNEL f1 ========
NUC1 13C
P1 6.00 usec
PL1 0.00 dB
SFO1 75.4760505 MHz
======== CHANNEL f2 ========
CPDPRG2 waltz16
NUC2 1H
PCPD2 100.00 usec
PL2 0.00 dB
PL12 23.41 dB
PL13 20.50 dB
SFO2 300.1312005 MHz
SI 32768
SF 75.4676597 MHz
WDW EM
SSB 0
LB 3.00 Hz
GB 0
PC 1.40

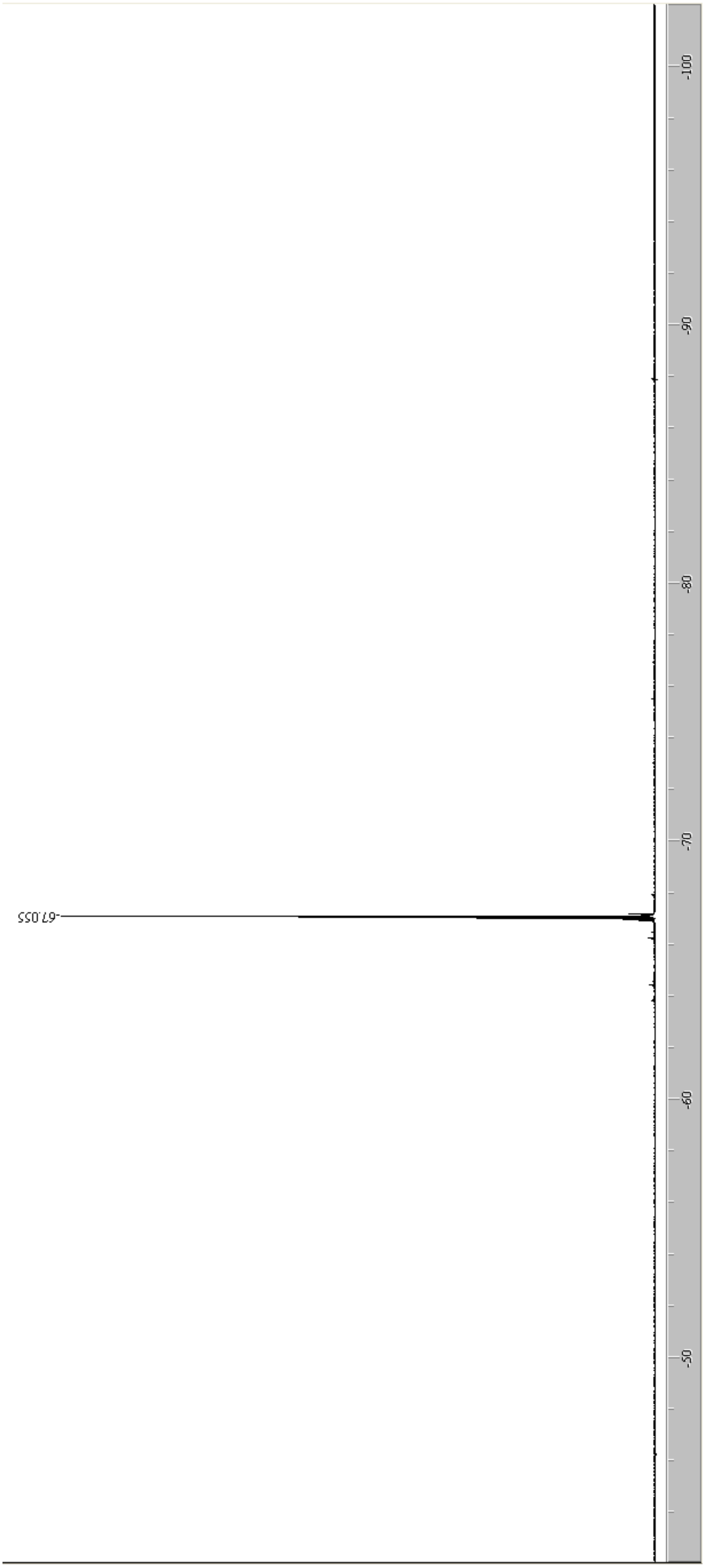
-67.055
-100
-90
-80
-70
-60
-50

3-(Trifluoromethyl)-3a, 4, 5, 6, 7, 8, 9,9a-octahydrocycloocta[*d*]isoxazole (**1h**)

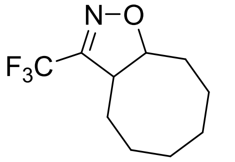

1h

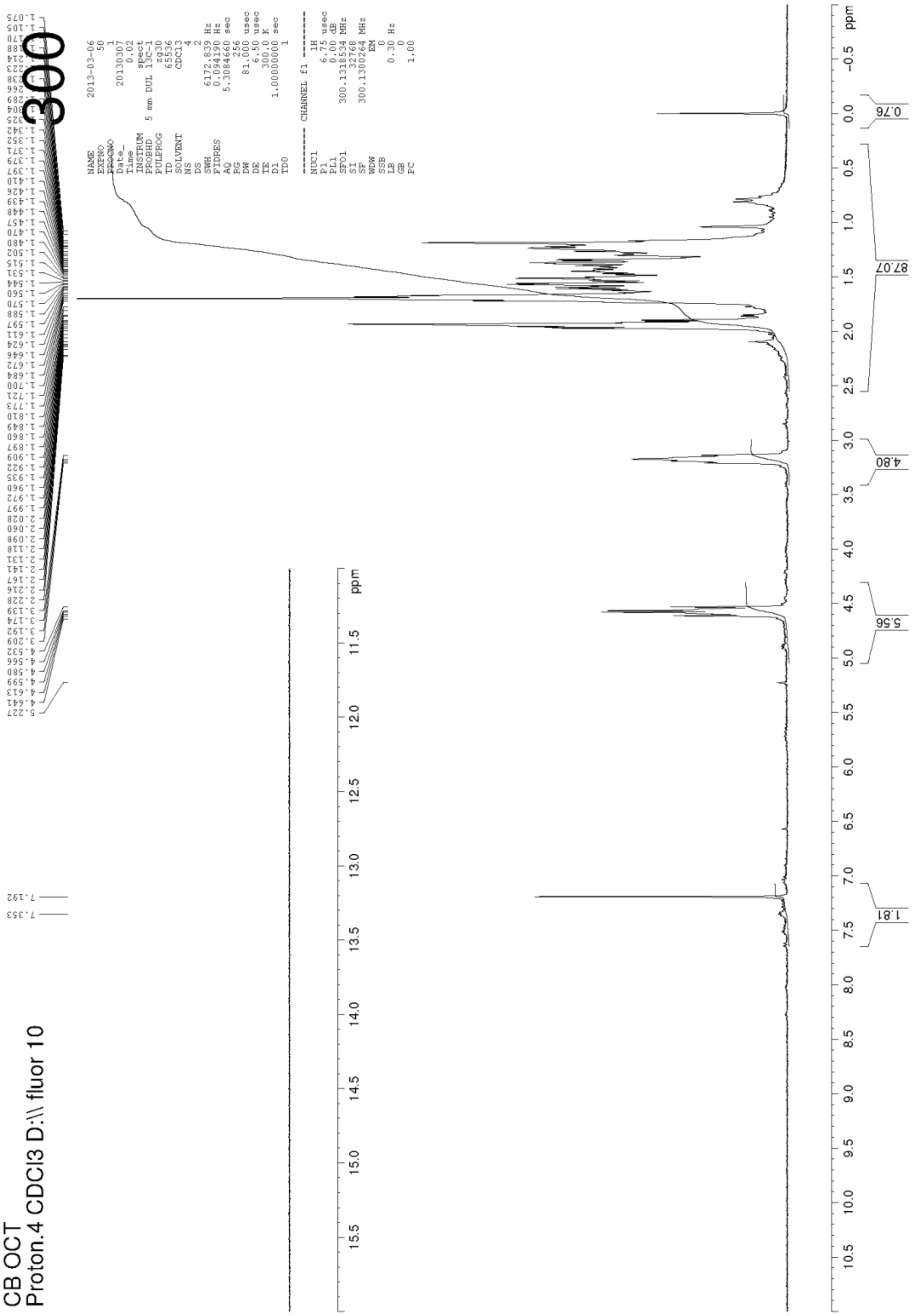

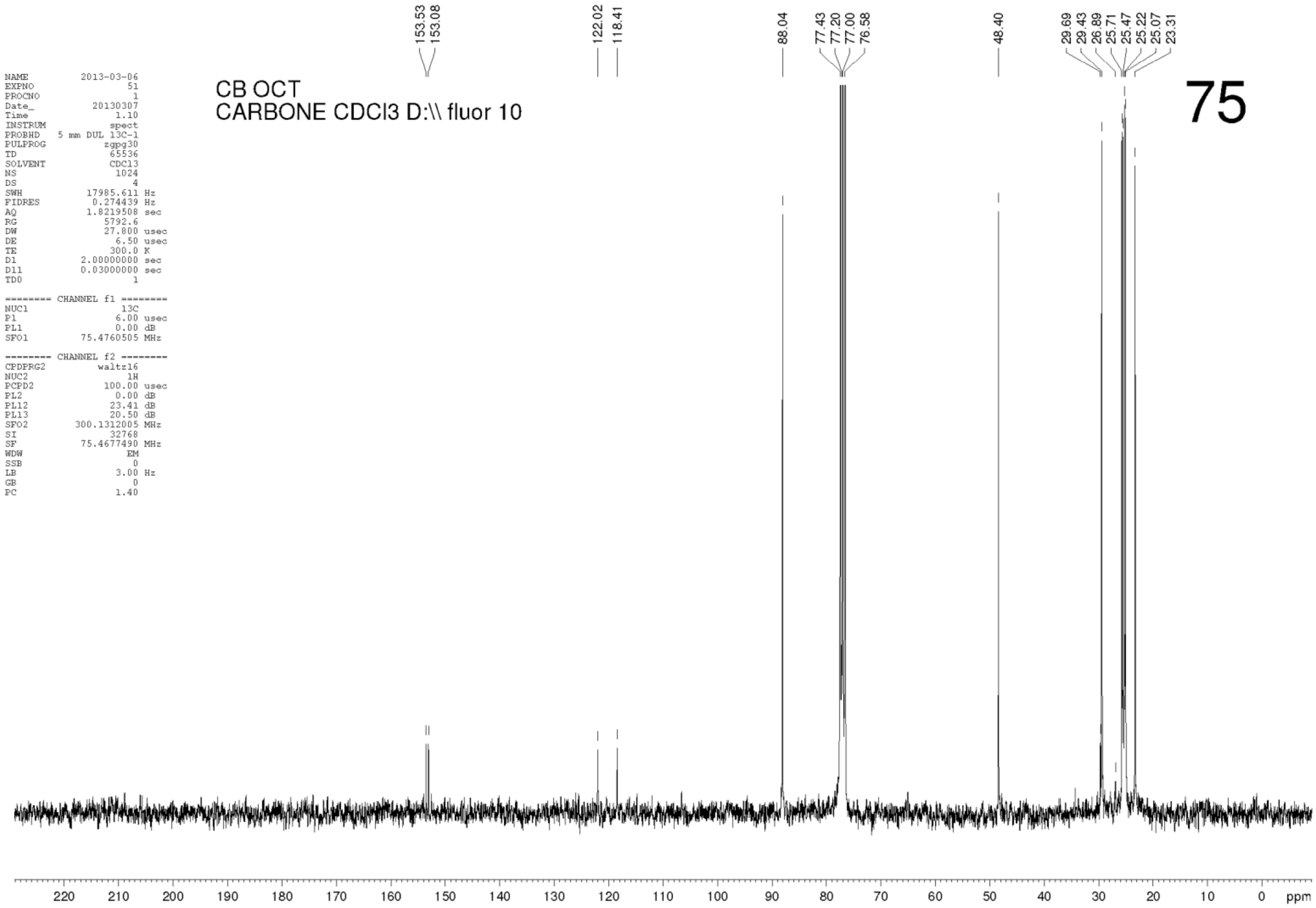
75
CB OCT
CARBONE CDCl3 D:\\ fluor 10
153.53
153.08
122.02
118.41
88.04
77.43
77.20
77.00
76.58
48.40
29.69
29.43
26.89
25.71
25.47
25.22
25.07
23.31
NAME 2013-03-06
EXPNO 51
PROCNO 1
Date_ 20130307
Time 1.10
INSTRUM spect
PROBHD 5 mm DUL 13C-1
PULPROG zgpg30
TD 65536
SOLVENT CDCl3
NS 1024
DS 4
SWH 17985.611 Hz
FIDRES 0.274439 Hz
AQ 1.8219508 sec
RG 5792.6
DW 27.800 usec
DE 6.50 usec
TE 300.0 K
D1 2.00000000 sec
D11 0.03000000 sec
TD0 1
======== CHANNEL f1 ========
NUC1 13C
P1 6.00 usec
PL1 0.00 dB
SFO1 75.4760505 MHz
======== CHANNEL f2 ========
CPDPRG2 waltz16
NUC2 1H
PCPD2 100.00 usec
PL2 0.00 dB
PL12 23.41 dB
PL13 20.50 dB
SFO2 300.1312005 MHz
SI 32768
SF 75.4677490 MHz
WDW EM
SSB 0
LB 3.00 Hz
GB 0
PC 1.40
220 210 200 190 180 170 160 150 140 130 120 110 100 90 80 70 60 50 40 30 20 10 0 ppm

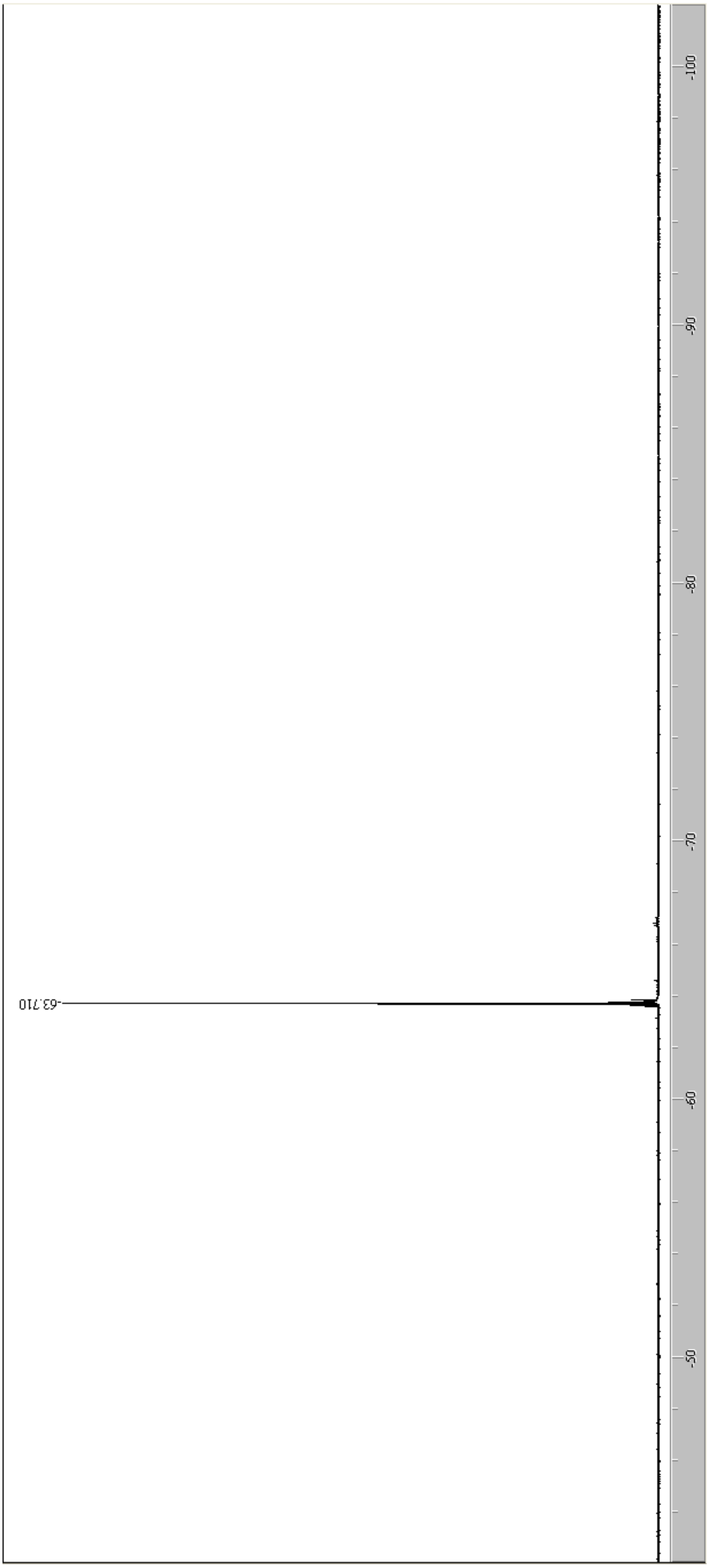
-63.710
-100
-90
-80
-70
-60
-50

Methyl 3-(trifluoromethyl)-4,5-dihydroisoxazole-5-carboxylate (**1i**)

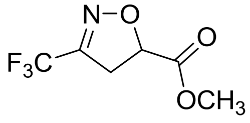

1i

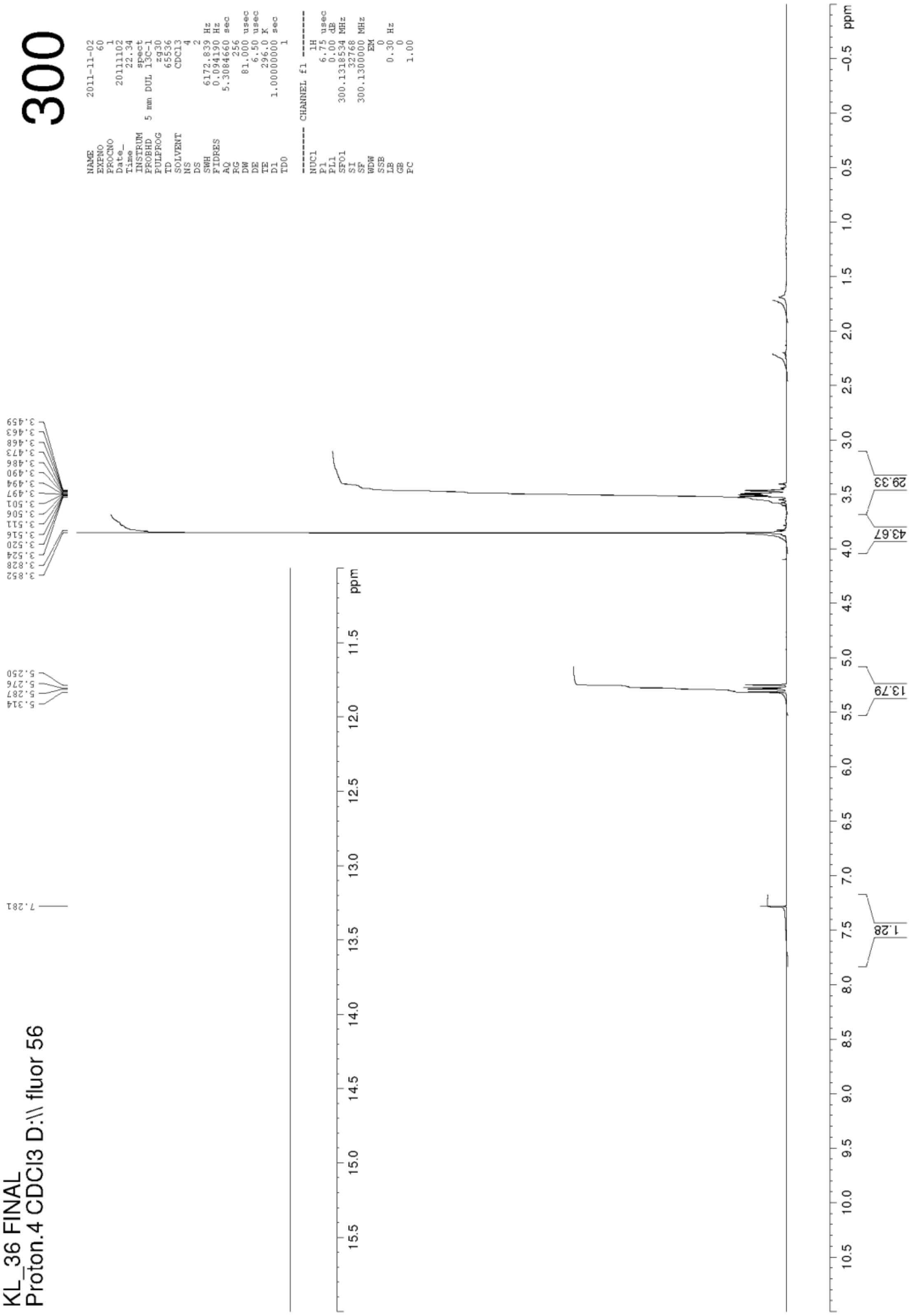

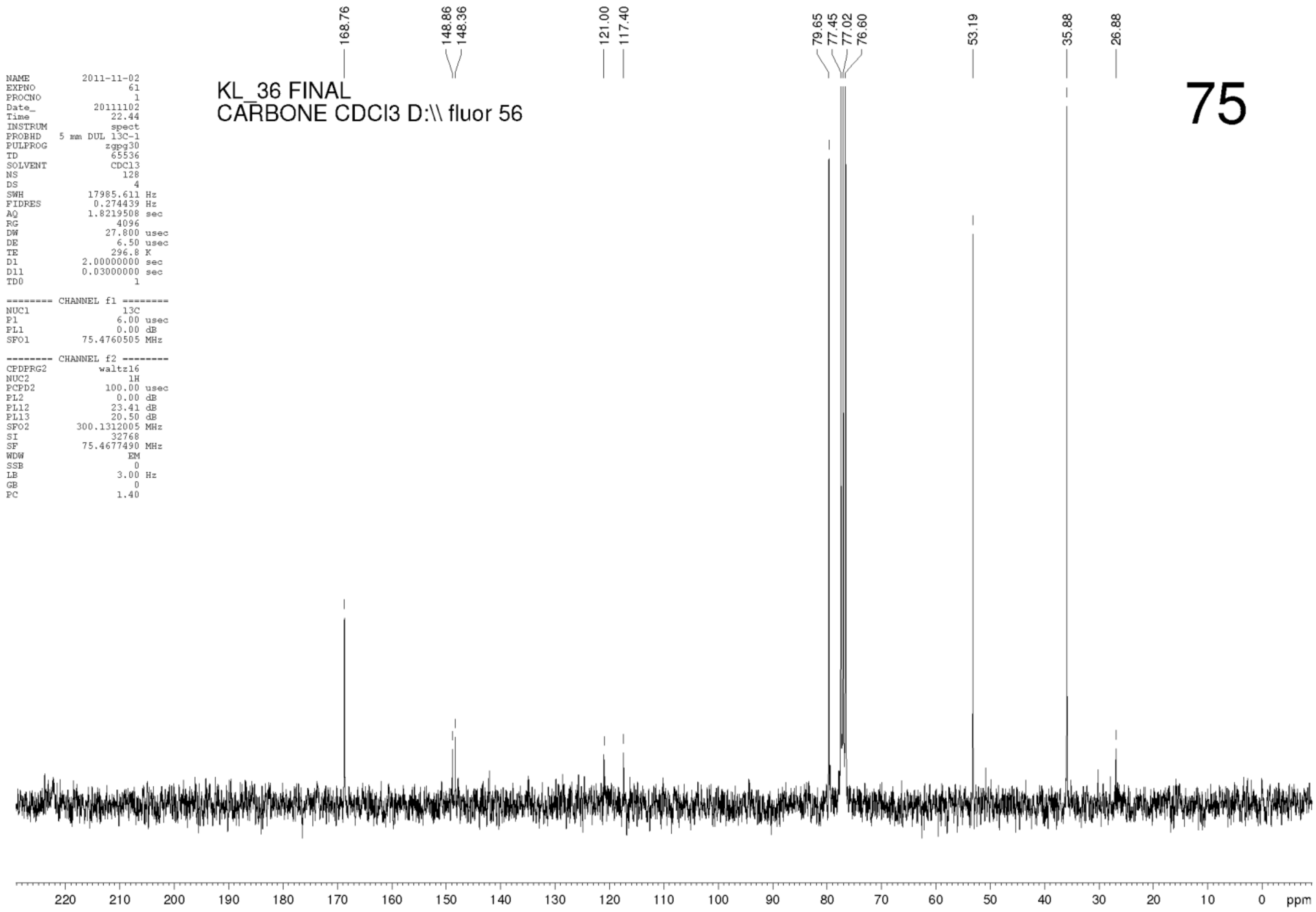
75
KL_36 FINAL
CARBONE CDCl3 D:\\ fluor 56
168.76
148.86
148.36
121.00
117.40
79.65
77.45
77.02
76.60
53.19
35.88
26.88
NAME 2011-11-02
EXPNO 61
PROCNO 1
Date_ 20111102
Time 22.44
INSTRUM spect
PROBHD 5 mm DUL 13C-1
PULPROG zgpg30
TD 65536
SOLVENT CDCl3
NS 128
DS 4
SWH 17985.611 Hz
FIDRES 0.274439 Hz
AQ 1.8219508 sec
RG 4096
DW 27.800 usec
DE 6.50 usec
TE 296.8 K
D1 2.00000000 sec
D11 0.03000000 sec
TD0 1
======== CHANNEL f1 ========
NUC1 13C
P1 6.00 usec
PL1 0.00 dB
SFO1 75.4760505 MHz
======== CHANNEL f2 ========
CPDPRG2 waltz16
NUC2 1H
PCPD2 100.00 usec
PL2 0.00 dB
PL12 23.41 dB
PL13 20.50 dB
SFO2 300.1312005 MHz
SI 32768
SF 75.4677490 MHz
WDW EM
SSB 0
LB 3.00 Hz
GB 0
PC 1.40
220 210 200 190 180 170 160 150 140 130 120 110 100 90 80 70 60 50 40 30 20 10 0 ppm

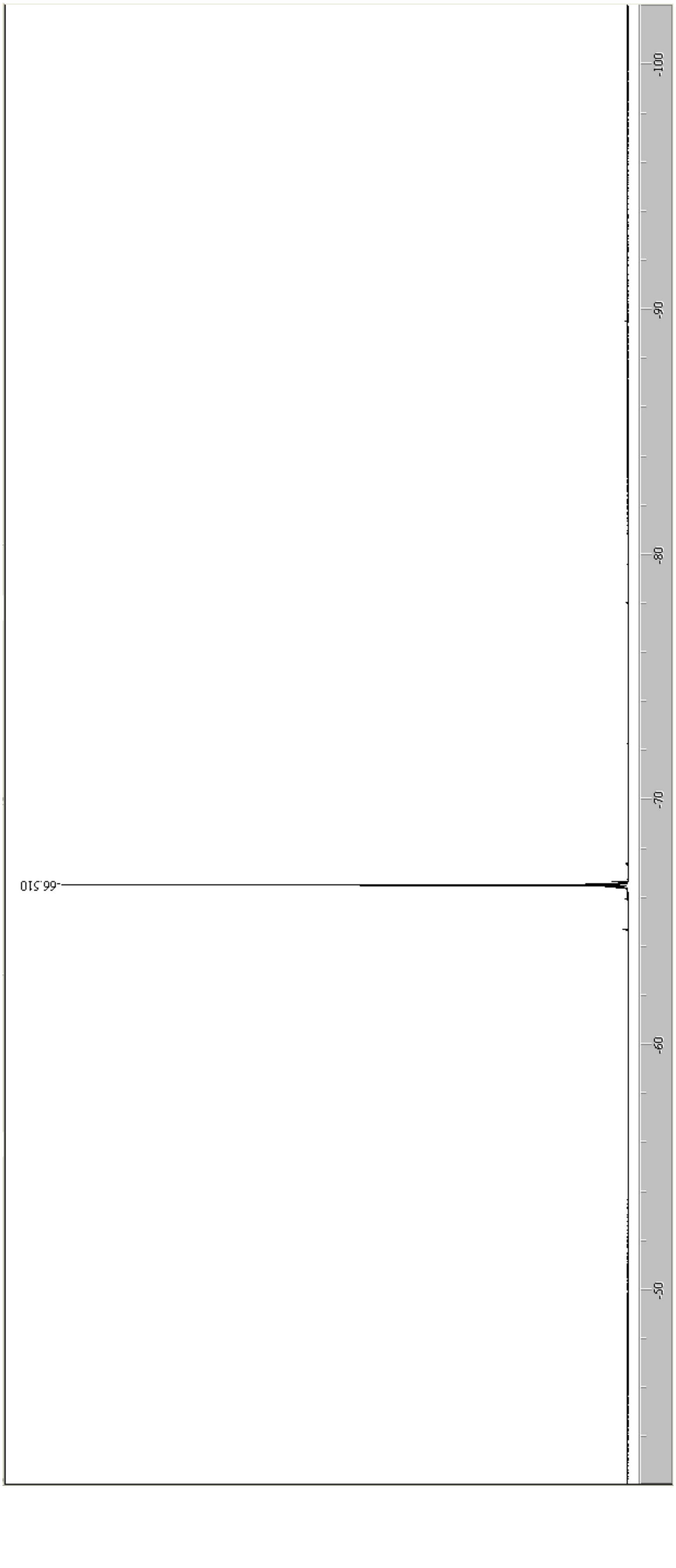
-66.510
-100
-90
-80
-70
-60
-50

5-(Bromomethyl)-3-(trifluoromethyl)-4,5-dihydroisoxazole (**1j**)

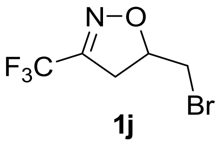

1j

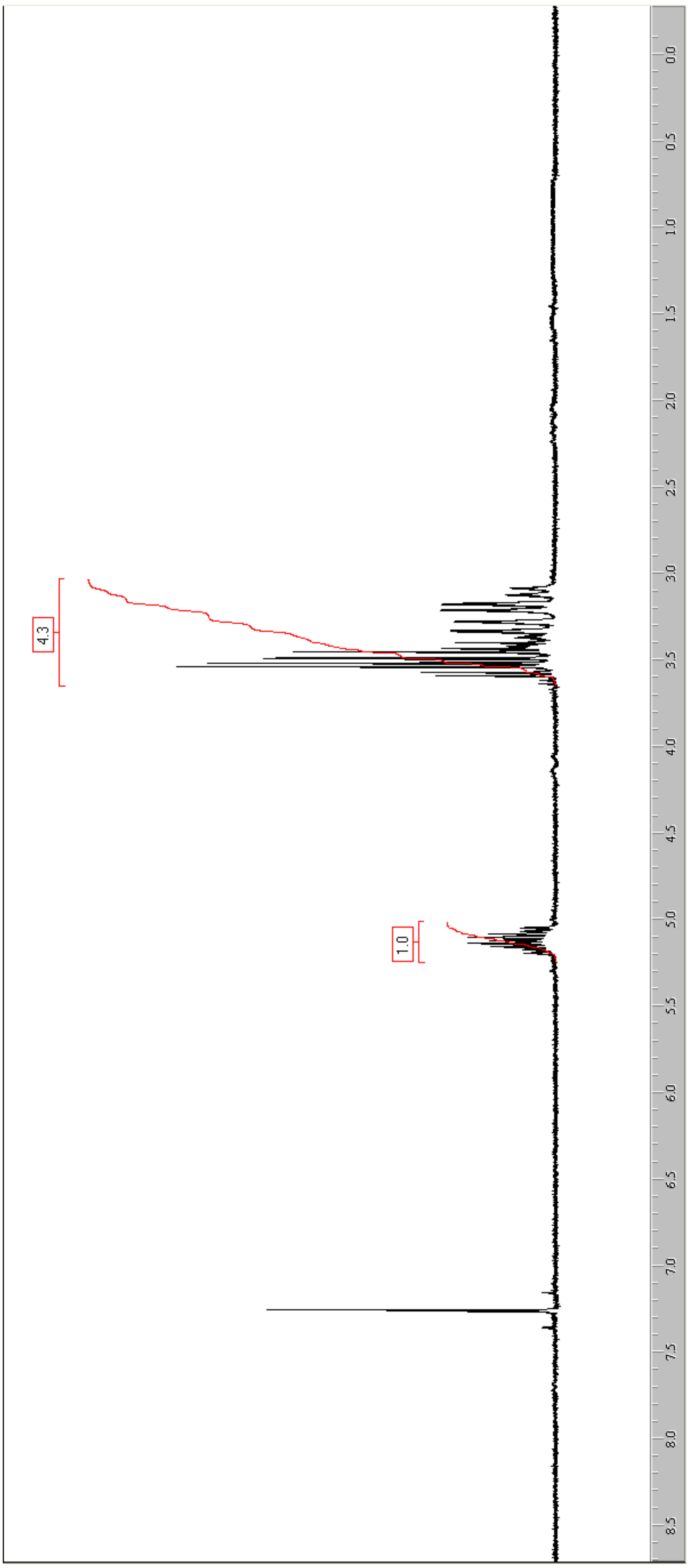

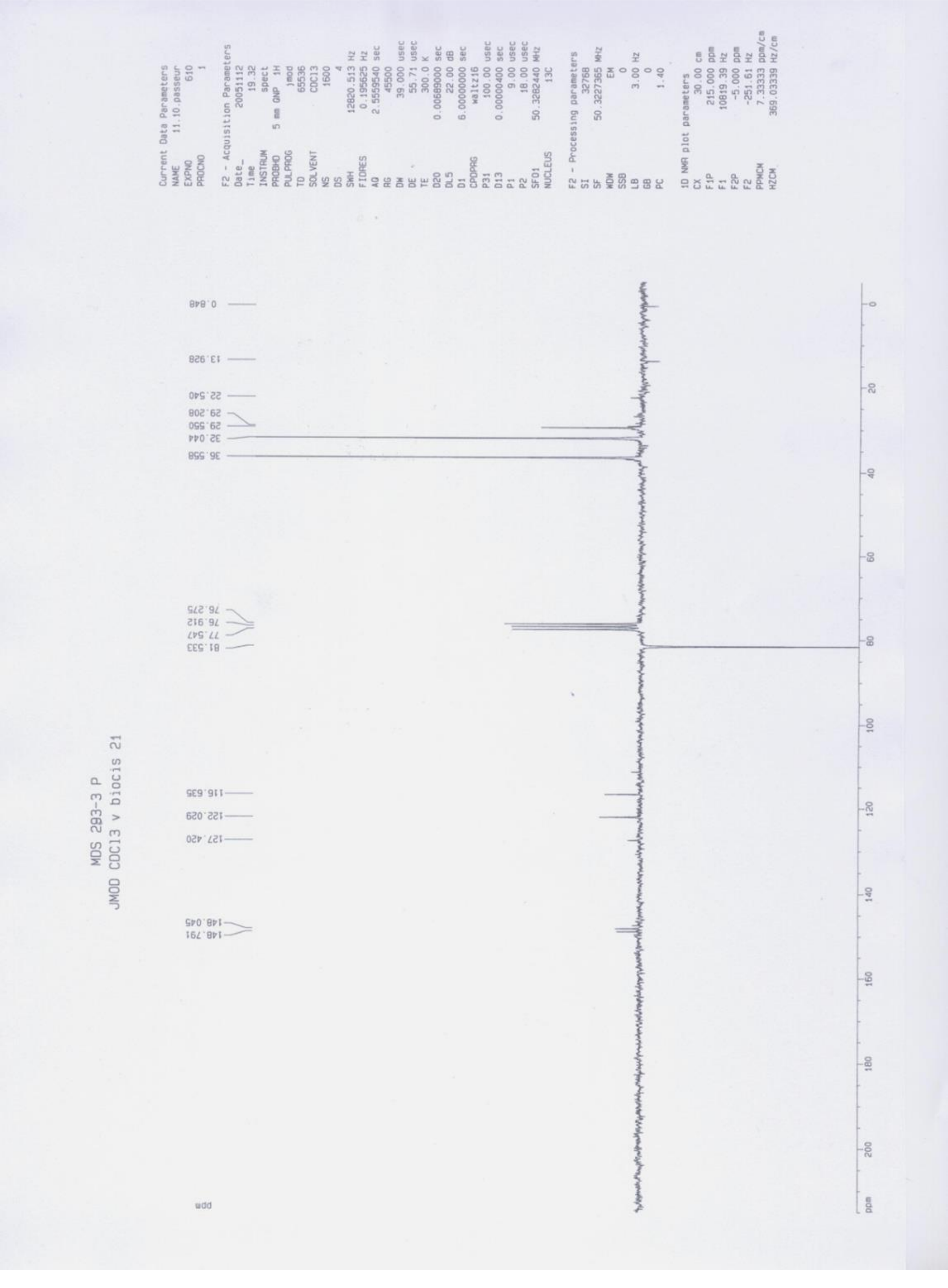
MDS 293-3 P
JMOD CDCl3 v biocis 21
0.848
13.928
22.540
29.208
29.550
32.044
36.558
76.275
76.912
77.547
81.533
116.635
122.029
127.420
148.045
148.791
ppm
Current Data Parameters
NAME 11.10.passeur
EXPNO 610
PROCNO 1
F2 - Acquisition Parameters
Date_ 20051112
Time 19.32
INSTRUM spect
PROBHD 5 mm QNP 1H
PULPROG jmod
TD 65536
SOLVENT CDCl3
NS 1600
DS 4
SWH 12820.513 Hz
FIDRES 0.195625 Hz
AQ 2.5559540 sec
RG 45500
DW 39.000 usec
DE 55.71 usec
TE 300.0 K
D20 0.00689000 sec
DL5 22.00 dB
D1 6.00000000 sec
CPDPRG waltz16
P31 100.00 usec
D13 0.00000400 sec
P1 9.00 usec
P2 18.00 usec
SFO1 50.3282440 MHz
NUCLEUS 13C
F2 - Processing parameters
SI 32768
SF 50.3227365 MHz
WDW EM
SSB 0
LB 3.00 Hz
GB 0
PC 1.40
1D NMR plot parameters
CX 30.00 cm
F1P 215.000 ppm
F1 10819.39 Hz
F2P -5.000 ppm
F2 -251.61 Hz
PPMCM 7.33333 ppm/cm
HZCM 369.03339 Hz/cm
0
20
40
60
80
100
120
140
160
180
200
ppm

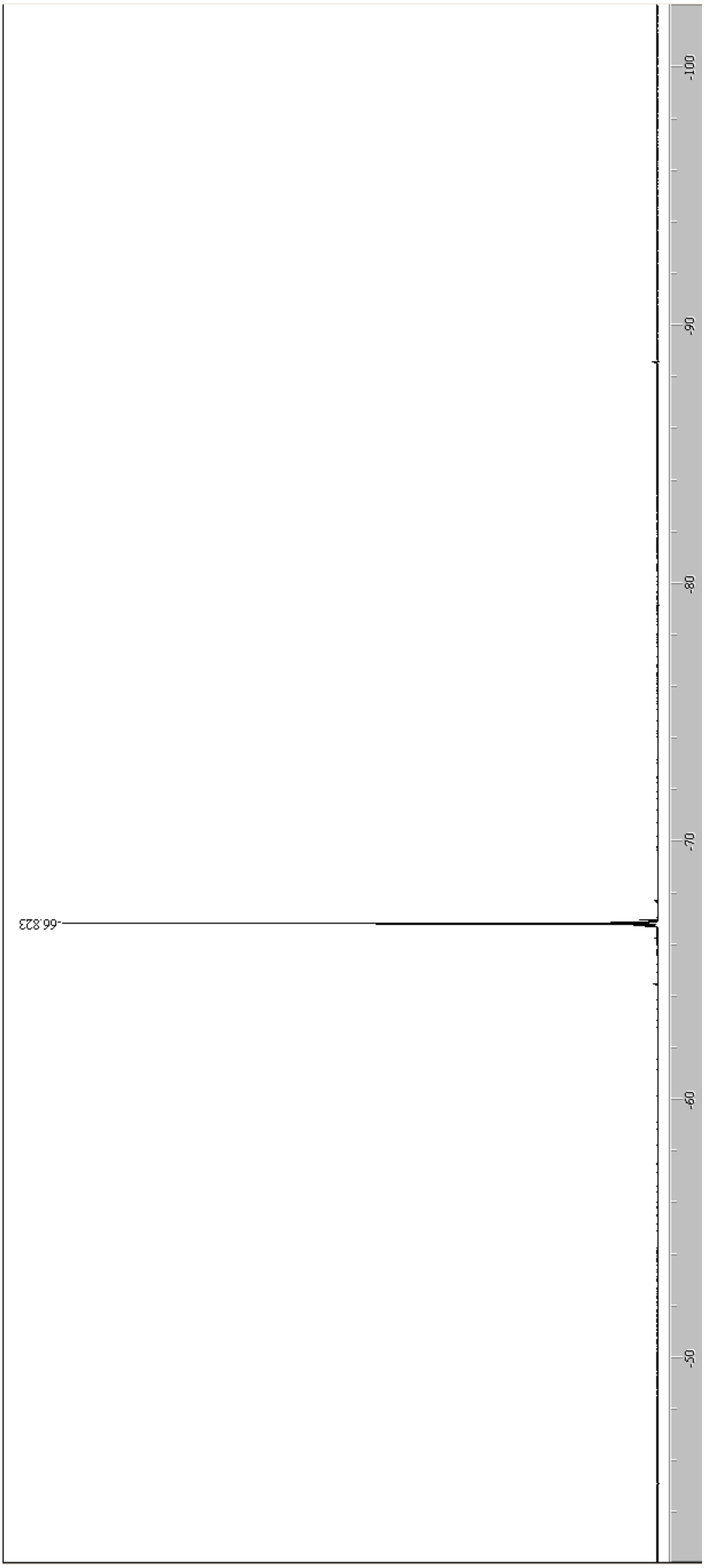
-66.823
-100
-90
-80
-70
-60
-50

5-Phenyl-3-(trifluoromethyl)isoxazole (**1l**)

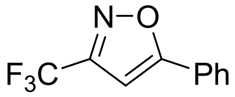

1l

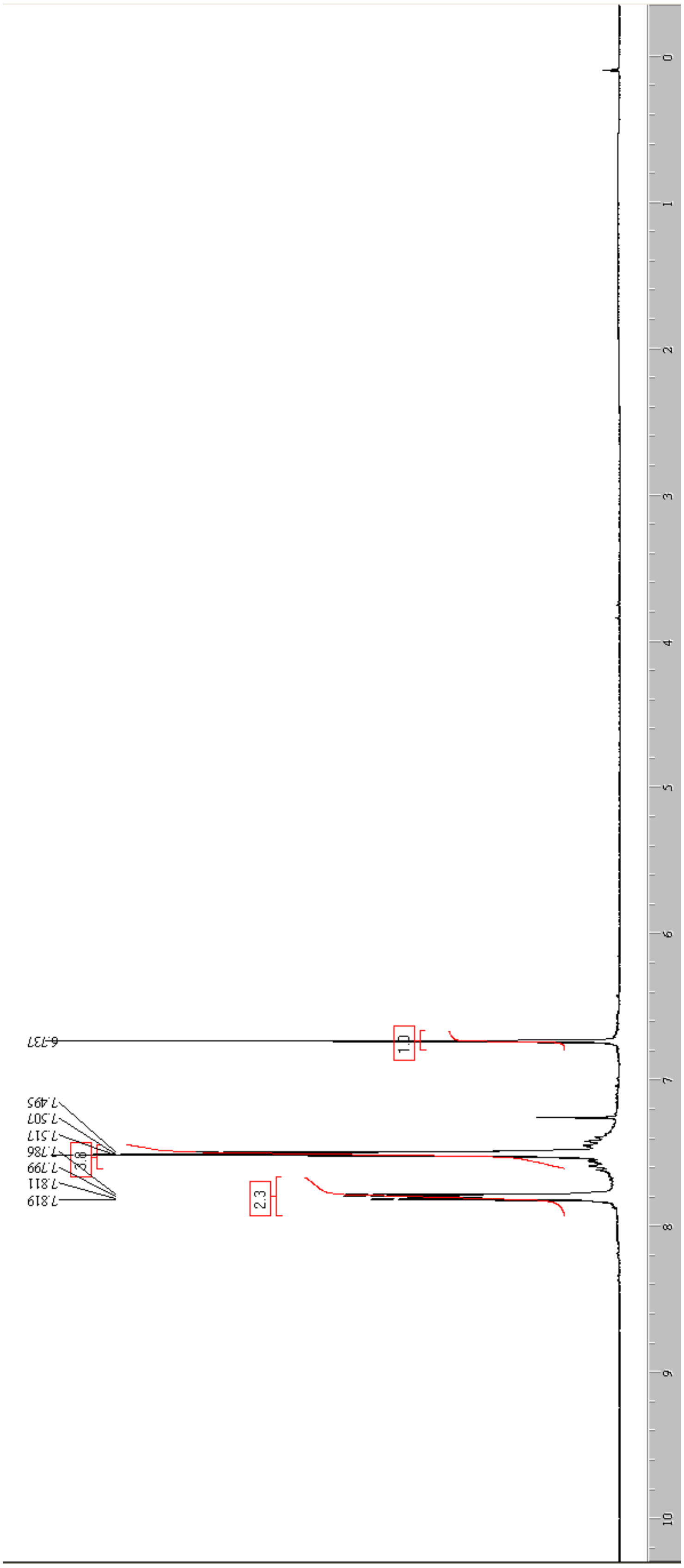

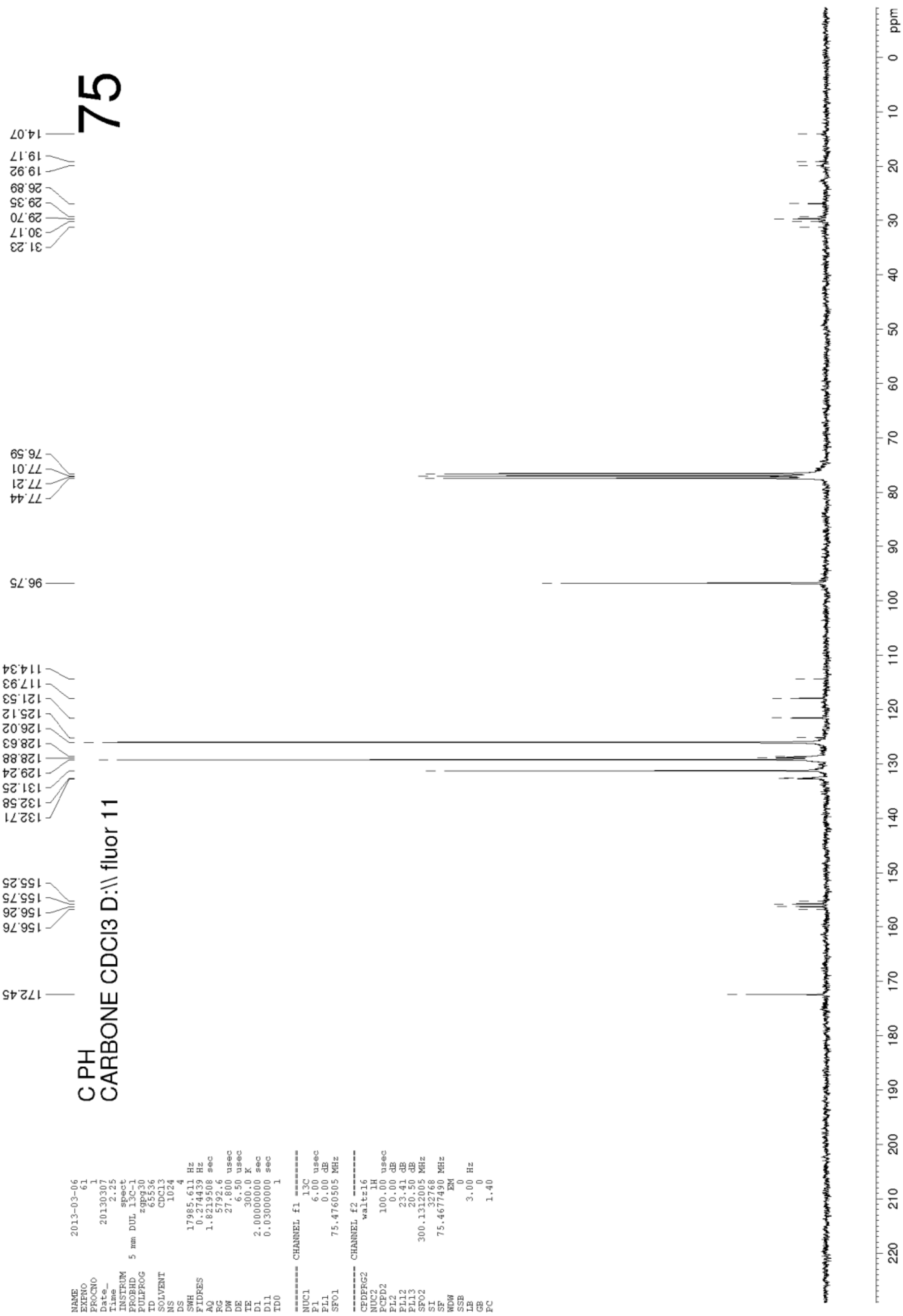
75
C PH
CARBONE CDCl3 D:\\ fluor 11
172.45
156.76
156.26
155.75
155.25
132.71
132.58
131.25
129.24
128.88
128.63
126.02
125.12
121.53
117.93
114.34
96.75
77.44
77.21
77.01
76.59
31.23
30.17
29.70
29.35
26.89
19.92
19.17
14.07
ppm
NAME 2013-03-06
EXPNO 61
PROCNO 1
Date_ 20130307
Time 2.25
INSTRUM spect
PROBHD 5 mm DUL 13C-1
PULPROG zgpg30
TD 65536
SOLVENT CDCl3
NS 1024
DS 4
SWH 17985.611 Hz
FIDRES 0.274439 Hz
AQ 1.8219508 sec
RG 5792.6
DW 27.800 usec
DE 6.50 usec
TE 300.0 K
D1 2.00000000 sec
D11 0.03000000 sec
TD0 1
======== CHANNEL f1 ========
NUC1 13C
P1 6.00 usec
PL1 0.00 dB
SFO1 75.4760505 MHz
======== CHANNEL f2 ========
CPDPRG2 waltz16
NUC2 1H
PCPD2 100.00 usec
PL2 0.00 dB
PL12 23.41 dB
PL13 20.50 dB
SFO2 300.1312005 MHz
SI 32768
SF 75.4677490 MHz
WDW EM
SSB 0
LB 3.00 Hz
GB 0
PC 1.40

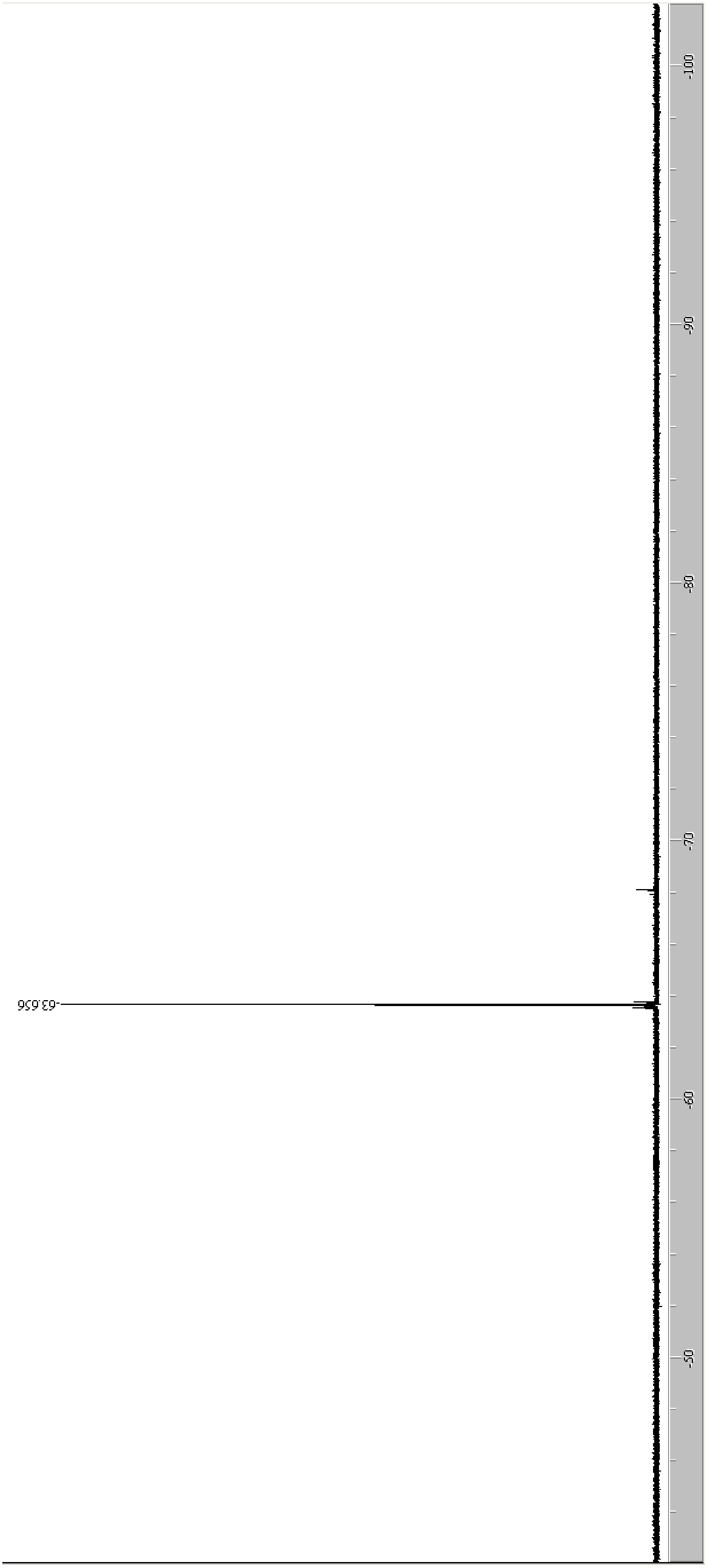

-63.656
-100
-90
-80
-70
-60
-50

3-(Trifluoromethyl)-5-(trimethylsilyl)isoxazole (**1m**)

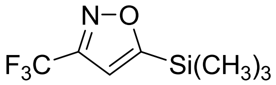

1m

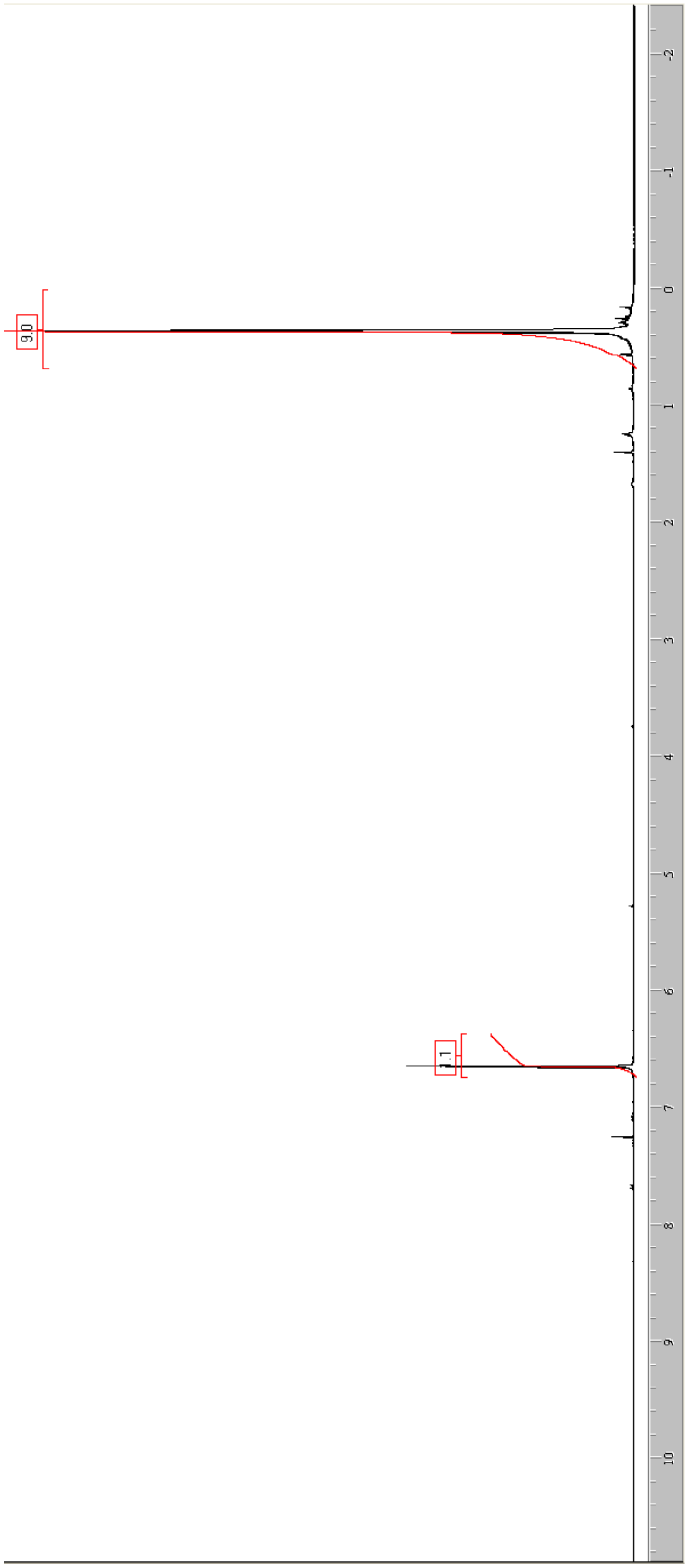

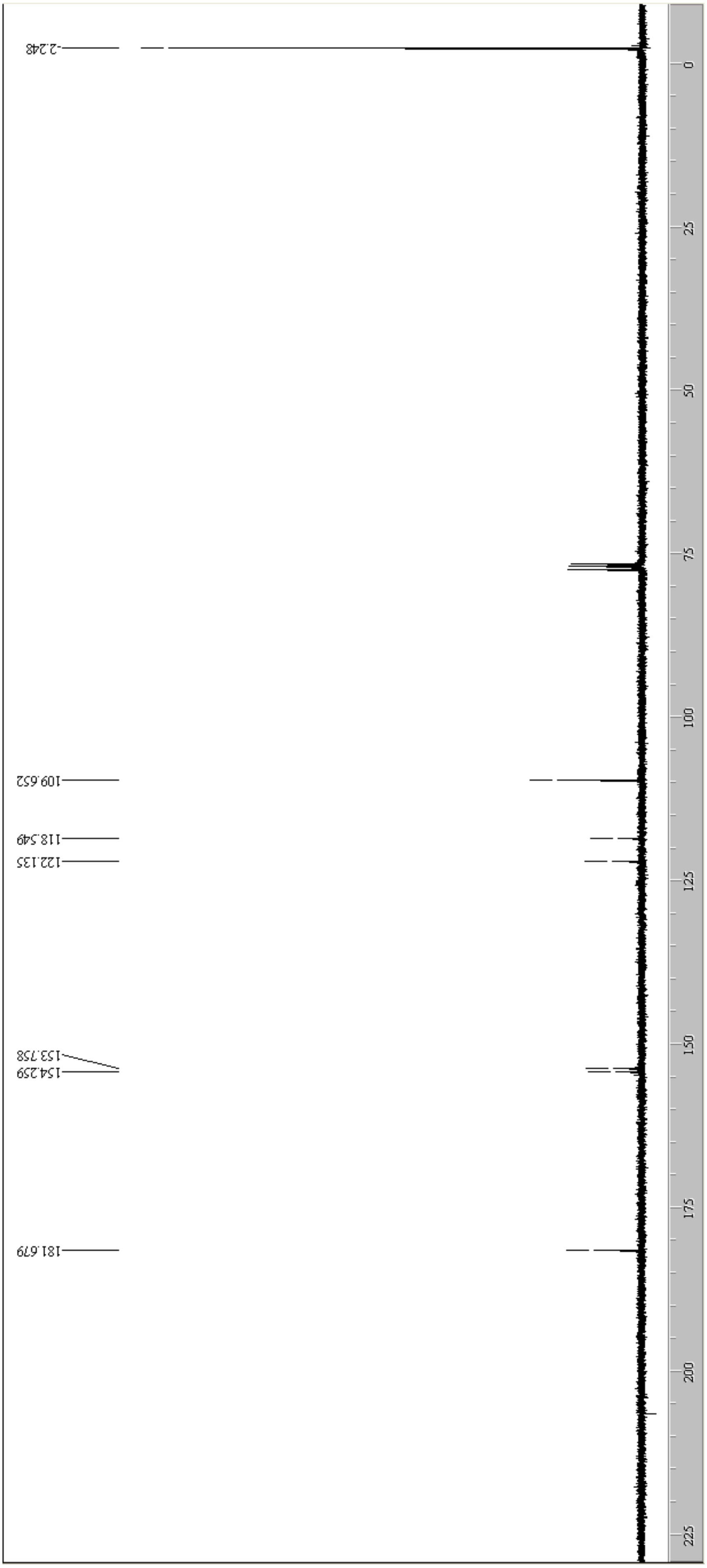
-2.248
109.652
118.549
122.135
153.758
154.259
181.679
0
25
50
75
100
125
150
175
200
225

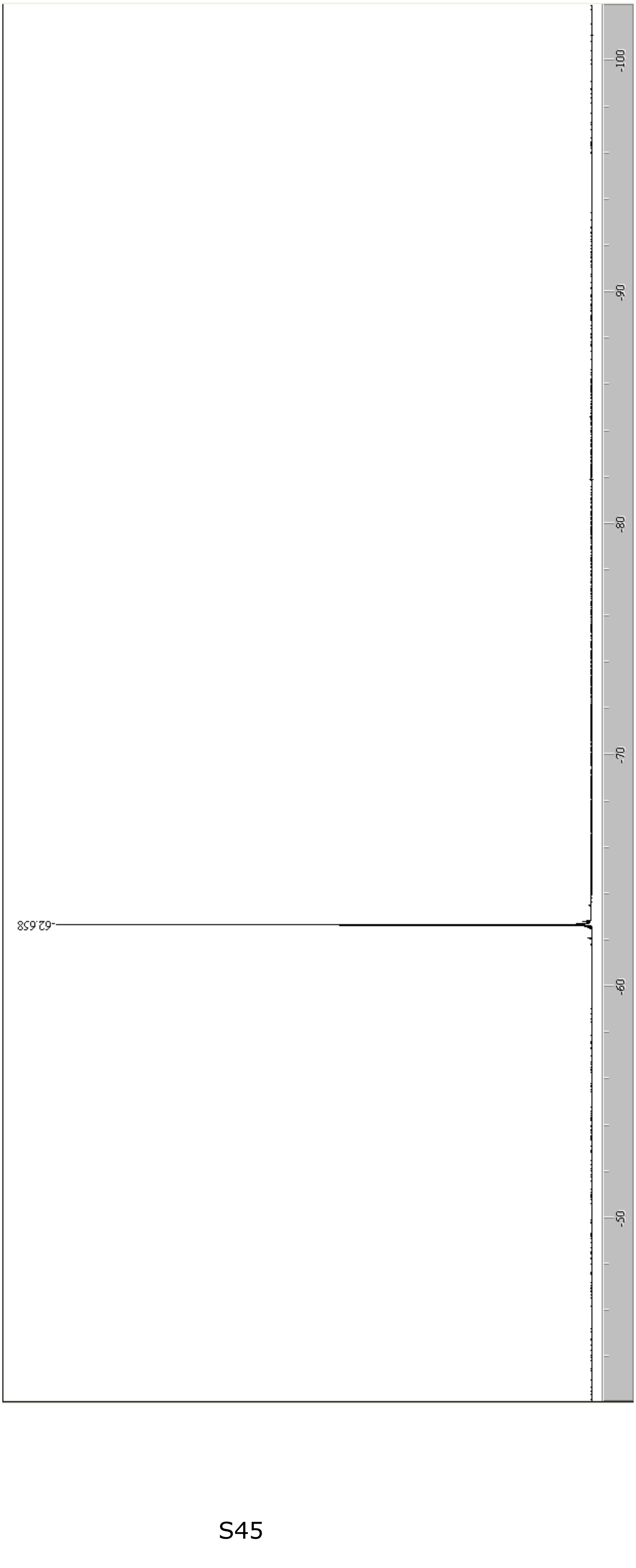
-62.658
-100
-90
-80
-70
-60
-50

4-Amino-5,5,5-trifluoro-1-phenylpentan-2-ol (**7a**)

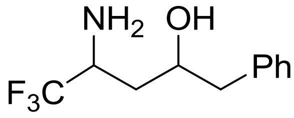

7a

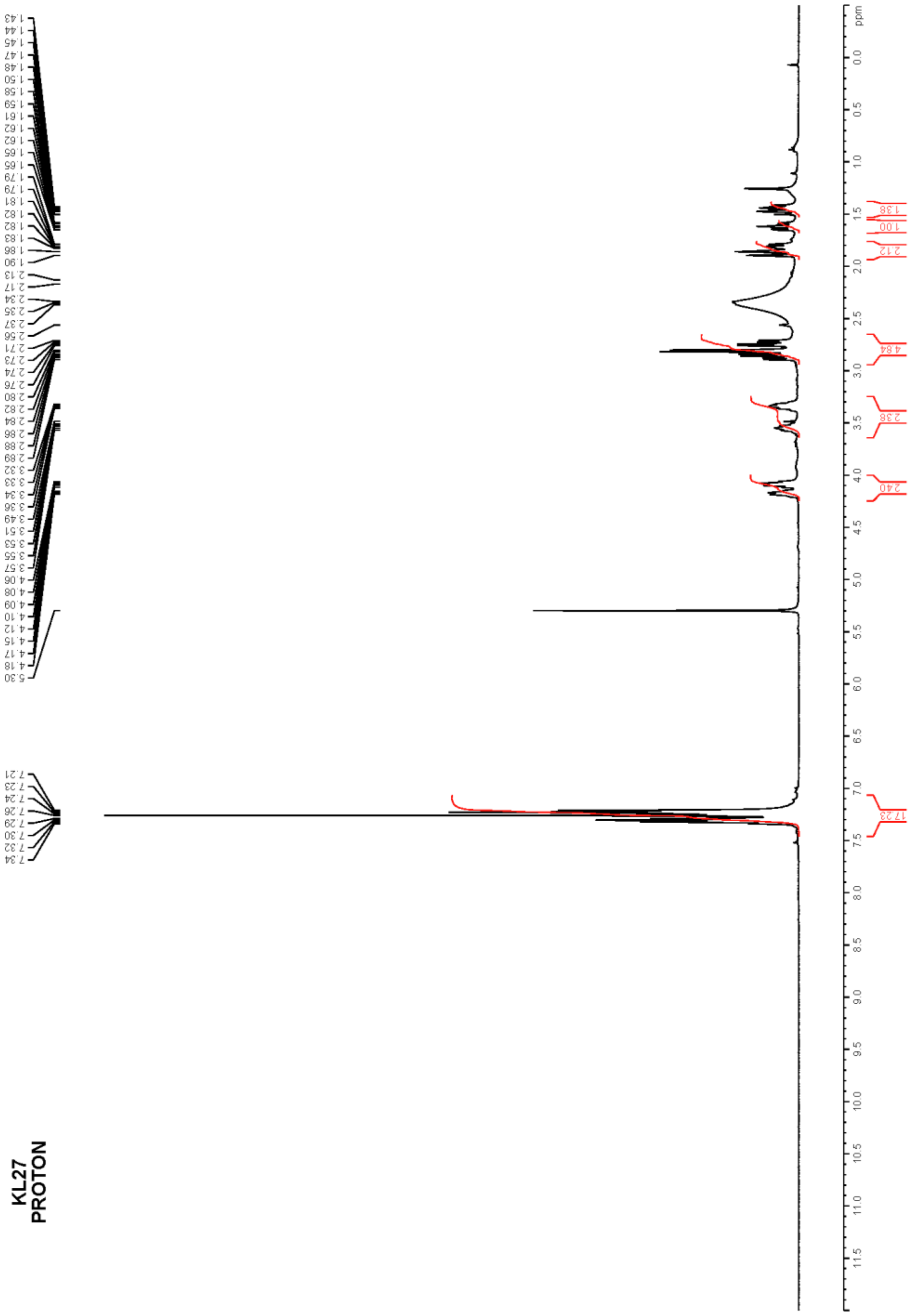

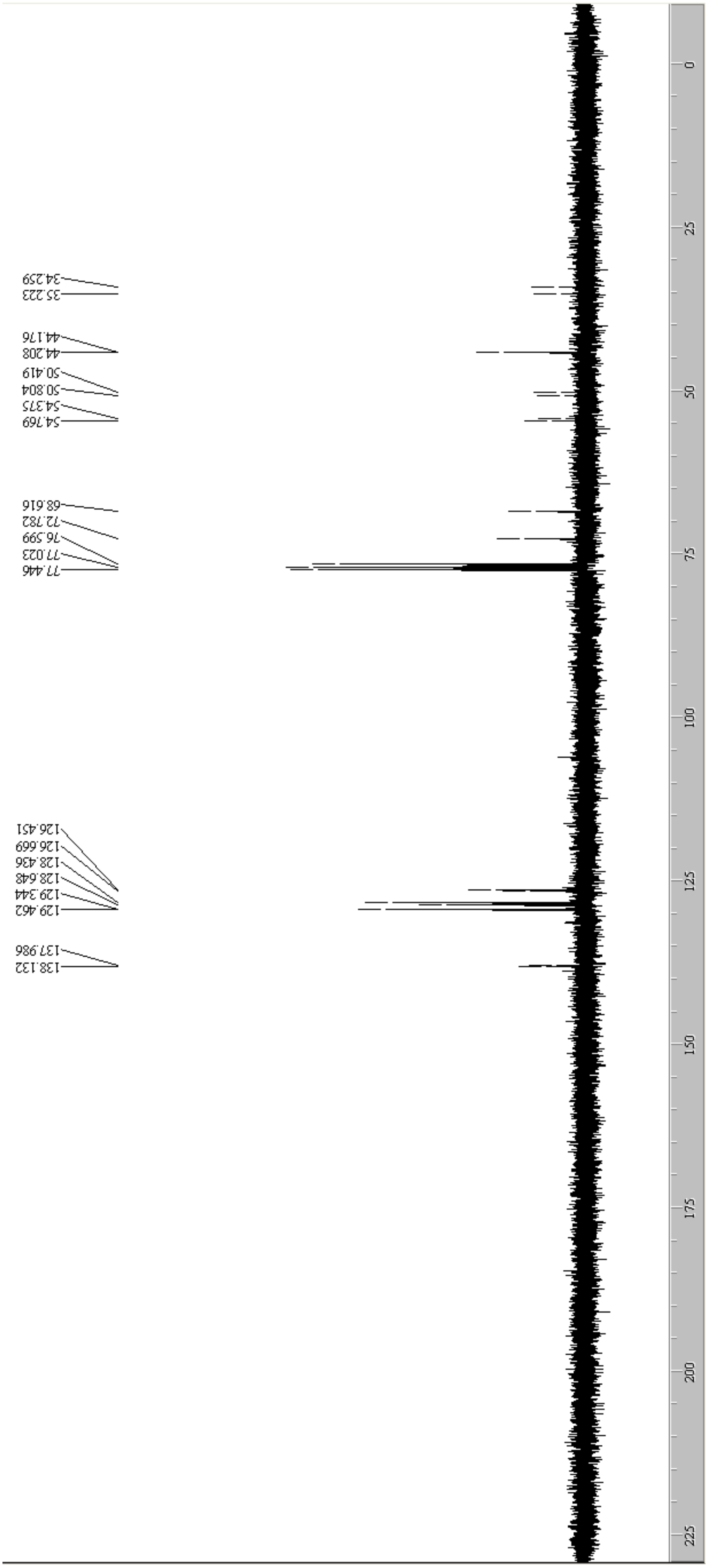

34.259
35.223
44.176
44.208
50.419
50.804
54.375
54.769
68.616
72.782
76.599
77.023
77.446
126.451
126.669
128.436
128.648
129.344
129.462
137.986
138.132
0
25
50
75
100
125
150
175
200
225

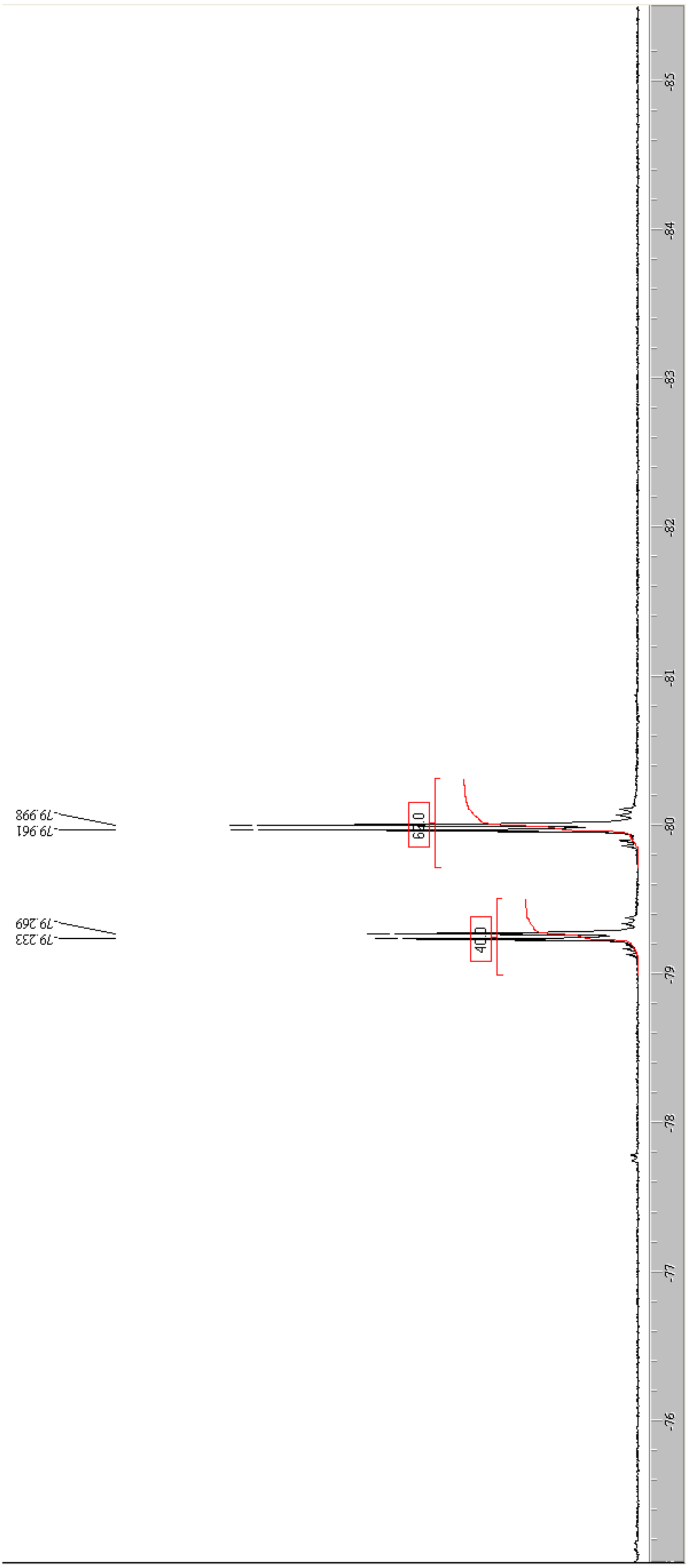

-79.998
-79.961
-79.269
-79.233
-85
-84
-83
-82
-81
-80
-79
-78
-77
-76

Benzyl 4-amino-5,5,5-trifluoro-2-hydroxypentylcarbamate (**7b**)

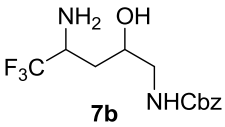

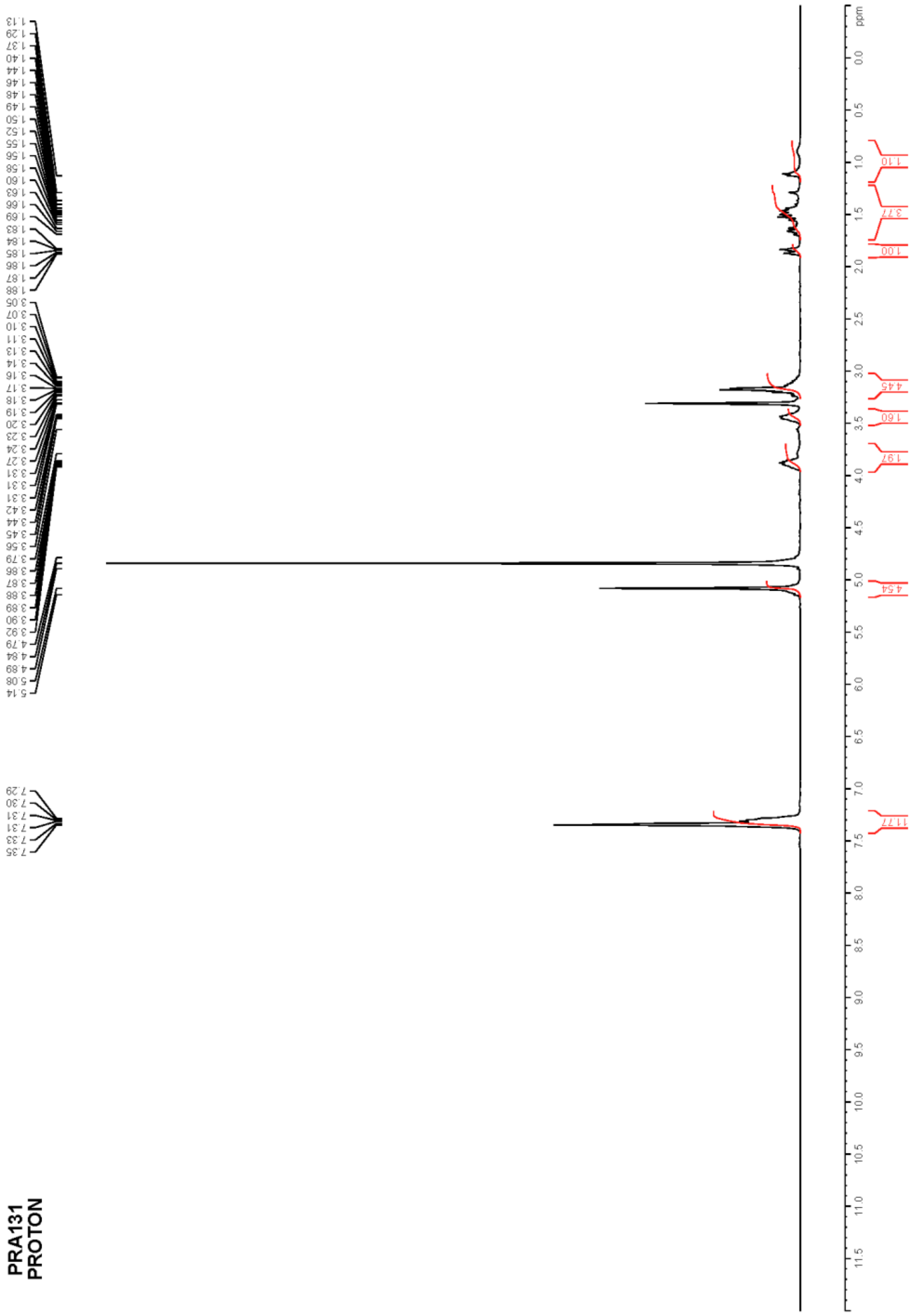

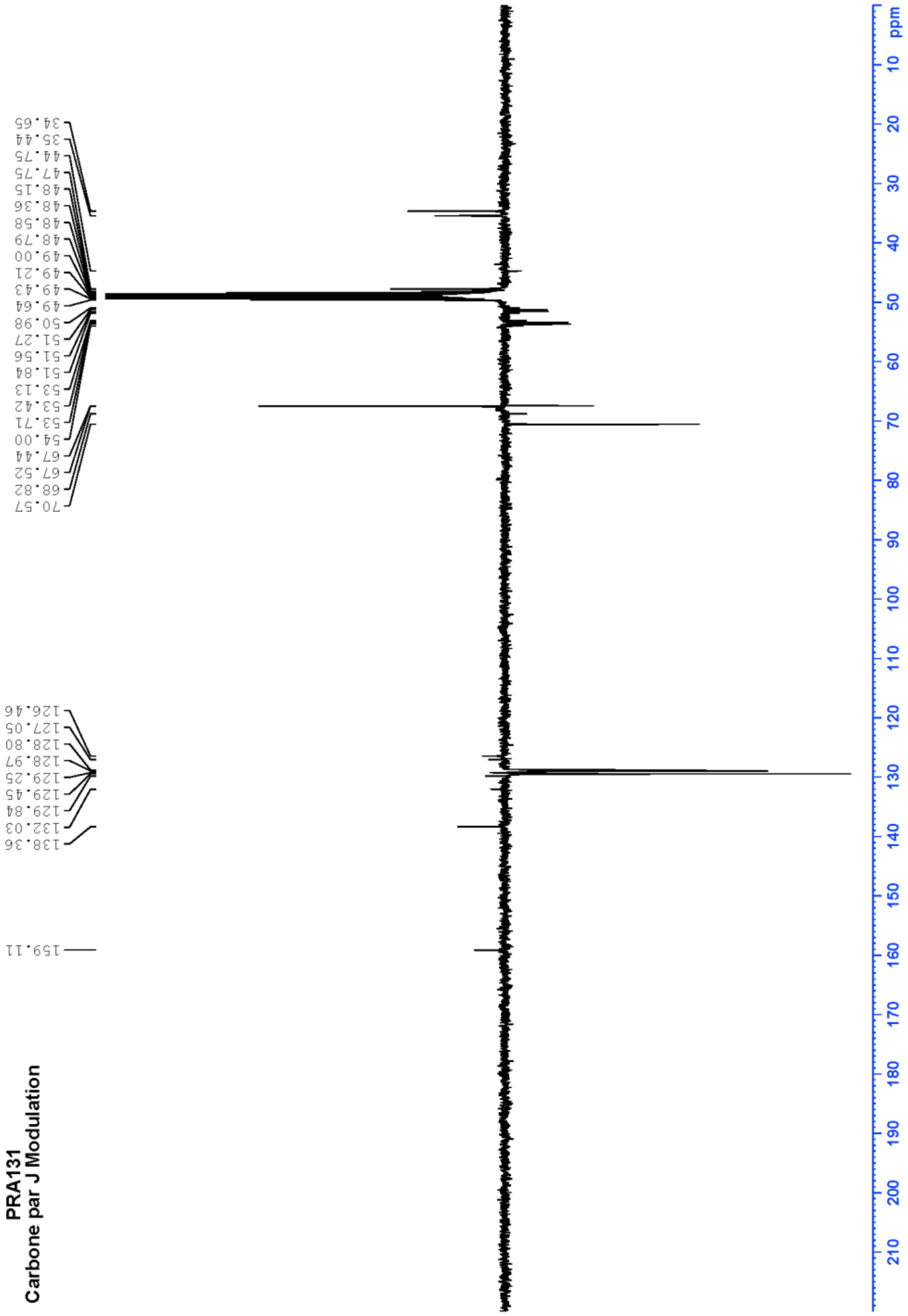
PRA131
Carbone par J Modulation
34.65
35.44
44.75
47.75
48.15
48.36
48.58
48.79
49.00
49.21
49.43
49.64
50.98
51.27
51.56
51.84
53.13
53.42
53.71
54.00
67.44
67.52
68.82
70.57
126.46
127.05
128.80
128.97
129.25
129.45
129.84
132.03
138.36
159.11
ppm
10
20
30
40
50
60
70
80
90
100
110
120
130
140
150
160
170
180
190
200
210

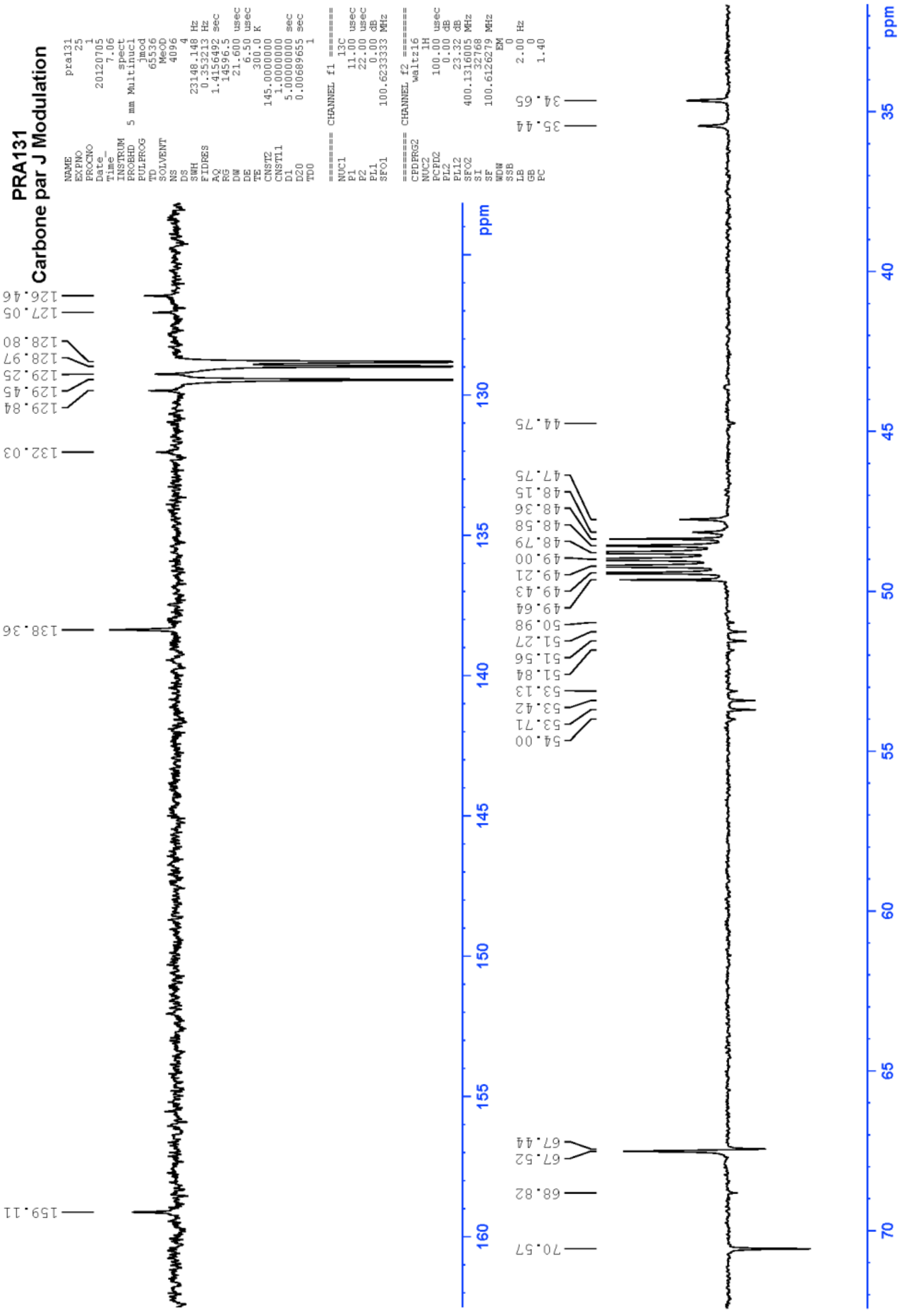
PRA131
Carbone par J Modulation
126.46
127.05
128.80
128.97
129.25
129.45
129.84
132.03
138.36
159.11
34.65
35.44
44.75
47.75
48.15
48.36
48.58
48.79
49.00
49.21
49.43
49.64
50.98
51.27
51.56
51.84
53.13
53.42
53.71
54.00
67.44
67.52
68.82
70.57
ppm

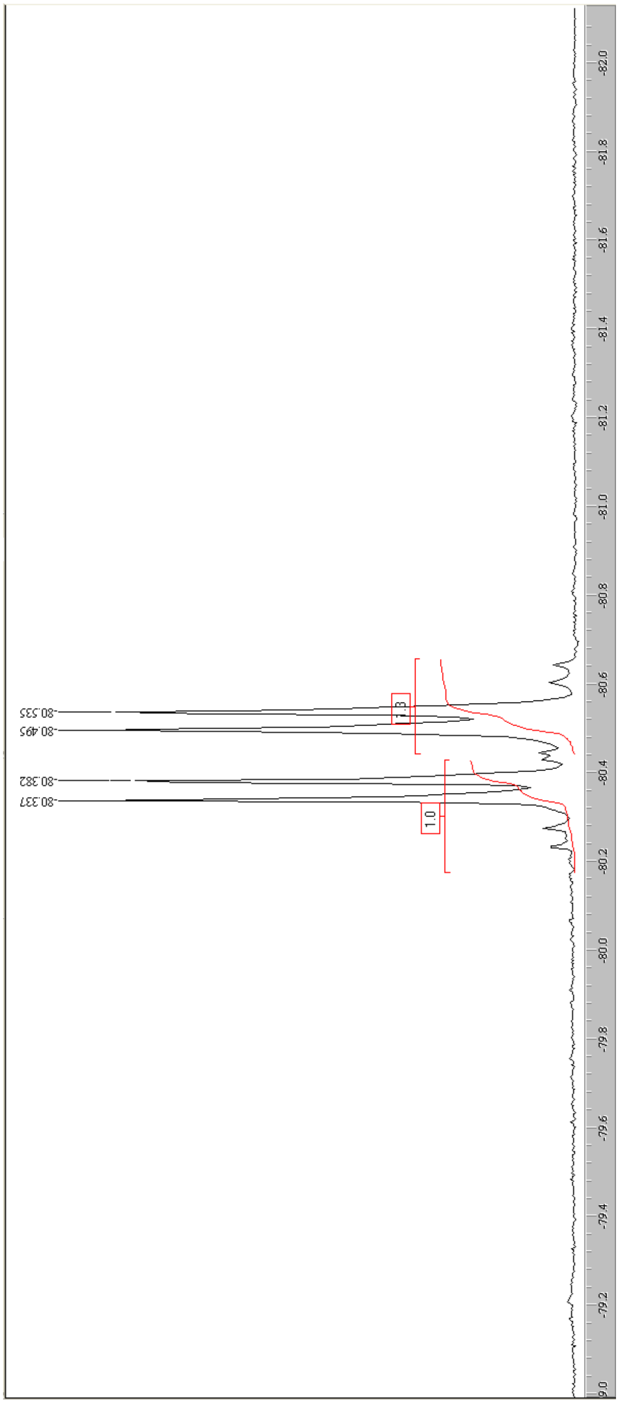

-80.535
-80.495
-80.382
-80.337
1.3
1.0
-82.0
-81.8
-81.6
-81.4
-81.2
-81.0
-80.8
-80.6
-80.4
-80.2
-80.0
-79.8
-79.6
-79.4
-79.2

3. Computational Methods

Conformations of reactants and products were fully optimized without constraint using DFT [2] method with the hybrid Becke3LYP functional [3] and the 6-31G* base [4] as implemented in the Gaussian 09 software package. [5] Vibrational analysis within the harmonic approximation was performed at the same level of theory upon geometrical optimization convergence and local minima were characterized by the absence of imaginary frequency. Figures are rendered with UCSF Chimera. [6]

Energies of Frontier Molecular Orbitals

Compound	HOMO level (eV)	LUMO level (eV)
**4**	−8.163	−1.024
**5a**	−6.430	0.016
**5b**	−6.660	−0.220
**5c**	−6.697	0.282
**5f**	−6.120	−1.149
**5g**	−6.318	0.744
**5h**	−6.331	0.708
**5i**	−7.425	−1.199
**5j**	−7.186	−0.779
**5k**	−7.697	−0.208
**5l**	−6.286	−0.777
**5m**	−7.233	0.706
**5n**	−7.566	−1.143

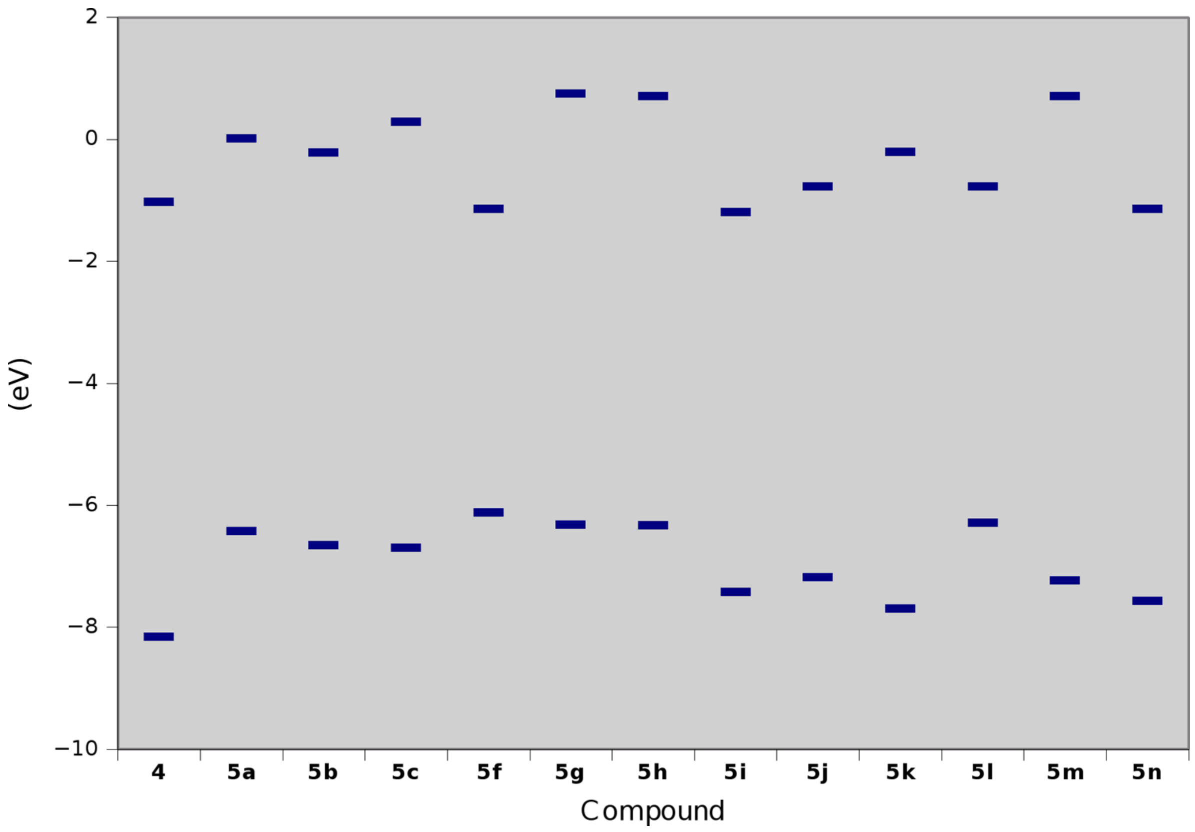

Model Coordinates for DFT Calculations

Compound **4** (E(RB3LYP) = −505.598294916 Ha, Lowest Frequency: 52.9685 cm^{-1}, Sum of electronic and thermal Free Energies = −505.602940 Ha.)

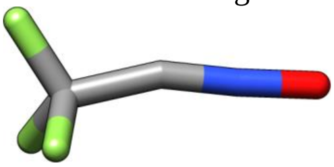

O	2.99240	0.01520	-0.00090
N	1.79300	-0.03630	0.00220
C	0.62960	-0.12600	0.00760
C	-0.83180	-0.00580	0.00030
F	-1.35720	-0.64340	-1.05860
F	-1.35940	-0.53910	1.11440
F	-1.20300	1.28510	-0.06200

Compound **5a** (E(RB3LYP) = −348.956968953 Ha, Lowest Frequency: 26.4344 cm^{-1}, Sum of electronic and thermal Free Energies = −348.828801 Ha.)

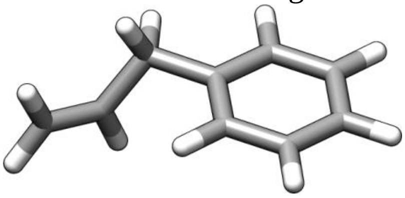

C	-3.47310	0.54740	0.11880
C	-2.41680	-0.17220	0.49950
C	-1.42770	-0.81840	-0.43830
C	0.00620	-0.35790	-0.21700
C	0.32790	1.00670	-0.23870
C	1.64240	1.43370	-0.05490
C	2.66090	0.50150	0.15880
C	2.35270	-0.85860	0.18740
C	1.03460	-1.28160	0.00170
H	-3.69950	0.71870	-0.93200
H	-4.15600	0.98790	0.84040
H	-2.22210	-0.31680	1.56280
H	-1.46780	-1.90980	-0.31470
H	-1.73090	-0.61300	-1.47430
H	-0.46290	1.73640	-0.39460
H	1.87270	2.49590	-0.07650
H	3.68540	0.83410	0.30380
H	3.13650	-1.59270	0.35590
H	0.80190	-2.34420	0.02460

Compound **5b** (E(RB3LYP) = −632.193524709 Ha, Lowest Frequency: 8.0603 cm^{-1}, Sum of electronic and thermal Free Energies = −632.015915 Ha.)

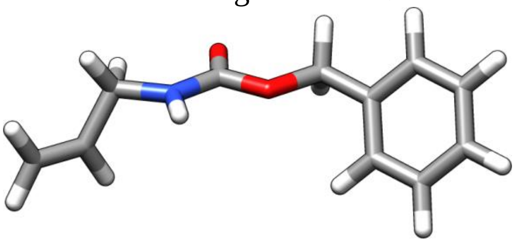

C	-5.50860	1.51150	-0.29010
C	-4.49310	0.85850	0.27480
C	-3.79050	-0.31900	-0.34860
N	-2.36330	-0.08320	-0.51490
C	-1.41400	-0.85080	0.09190
O	-1.62480	-1.82720	0.79020

O	-0.17480	-0.36580	-0.20390
C	0.92300	-1.11100	0.36640
C	2.19310	-0.32890	0.15180
C	3.29190	-0.91910	-0.47980
C	4.48410	-0.21030	-0.64550
C	4.58390	1.10330	-0.18800
C	3.48790	1.70390	0.43820
C	2.30310	0.99120	0.61020
H	-5.89920	1.22480	-1.26480
H	-5.99630	2.34950	0.20020
H	-4.11630	1.16830	1.24960
H	-3.87070	-1.20460	0.29010
H	-4.25060	-0.56350	-1.31550
H	-2.05000	0.71960	-1.04210
H	0.71720	-1.27220	1.42980
H	0.97710	-2.09570	-0.10940
H	3.21500	-1.94050	-0.84530
H	5.33000	-0.68330	-1.13730
H	5.50890	1.65850	-0.31880
H	3.55940	2.72790	0.79560
H	1.45050	1.46030	1.09420

Compound **5c** (E(RB3LYP) = −519.092463571 Ha, Lowest Frequency: 18.2678 cm^{-1}, Sum of electronic and thermal Free Energies = −518.907129 Ha.)

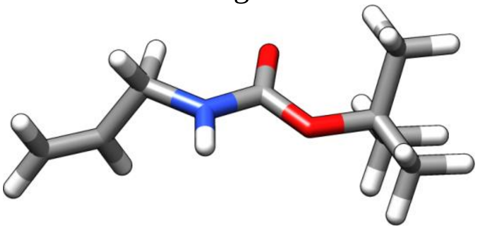

C	-4.73320	0.64420	0.14790
C	-3.52150	0.21390	0.49920
C	-2.57470	-0.50540	-0.42580
N	-1.29810	0.18210	-0.55370
C	-0.11830	-0.36650	-0.13380
O	0.00090	-1.49730	0.31110
O	0.87620	0.54270	-0.30250
C	2.26710	0.21350	0.04100
C	2.76040	-0.95430	-0.82030
C	3.01110	1.50310	-0.31700
C	2.38470	-0.08090	1.54070
H	-5.38990	1.14890	0.85120
H	-5.12030	0.49780	-0.85890
H	-3.15410	0.37720	1.51250
H	-2.33520	-1.50190	-0.04090
H	-3.03750	-0.63200	-1.41370
H	-1.28310	1.14820	-0.84790
H	2.62580	-0.72530	-1.88340
H	3.82890	-1.11930	-0.64080
H	2.21770	-1.87090	-0.58450
H	2.62450	2.34670	0.26440
H	4.07920	1.39410	-0.10110
H	2.89400	1.73480	-1.38070
H	1.83750	-0.98660	1.80670
H	3.43920	-0.21360	1.80850
H	1.98920	0.75760	2.12480

Compound **5f** (E(RB3LYP) = −2880.75323894 Ha, Lowest Frequency: 43.1560 cm^{-1}, Sum of electronic and thermal Free Energies = −2880.664160 Ha.)

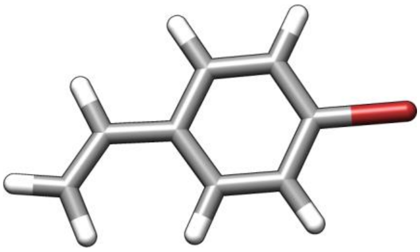

C	-4.50750	-0.59130	-0.00010
C	-3.58430	0.37860	0.00000
C	-2.12130	0.22350	0.00000
C	-1.47660	-1.02720	0.00010
C	-0.08940	-1.12610	0.00000
C	0.68000	0.03840	-0.00000
Br	2.58680	-0.09420	-0.00000
C	0.07700	1.29310	-0.00000
C	-1.31430	1.37380	0.00000
H	-4.25520	-1.64830	-0.00020
H	-5.56640	-0.35240	-0.00010
H	-3.92800	1.41290	0.00010
H	-2.06340	-1.94080	0.00010
H	0.39400	-2.09710	0.00010
H	0.68420	2.19180	-0.00000
H	-1.78540	2.35380	-0.00000

Compound **5g** (E(RB3LYP) = −526.581273708 Ha, Lowest Frequency: 50.5033 cm^{-1}, Sum of electronic and thermal Free Energies = −526.435413 Ha.)

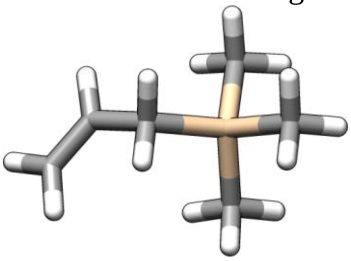

C	3.05710	-0.29610	0.04520
C	2.04280	0.52080	-0.25400
C	0.82160	0.15740	-1.04710
Si	-0.76140	-0.02430	0.01820
C	-1.05090	1.56650	1.00700
C	-2.23000	-0.32540	-1.14240
C	-0.55700	-1.48890	1.19850
H	3.89710	0.03430	0.65020
H	3.08770	-1.32480	-0.30920
H	2.07280	1.54350	0.12630
H	0.62260	0.92410	-1.81070
H	0.98200	-0.78800	-1.58350
H	-1.15380	2.43750	0.34810
H	-1.96770	1.49710	1.60530
H	-0.22280	1.76770	1.69680
H	-2.08900	-1.23340	-1.74120
H	-3.16380	-0.44460	-0.57920
H	-2.36920	0.51120	-1.83790
H	0.34120	-1.37270	1.81570
H	-1.41780	-1.58260	1.87160
H	-0.46130	-2.43420	0.65040

Compound **5h** (E(RB3LYP) = −313.264478145 Ha, Lowest Frequency: 141.1004 cm^{-1}, Sum of electronic and thermal Free Energies = −313.090863 Ha.)

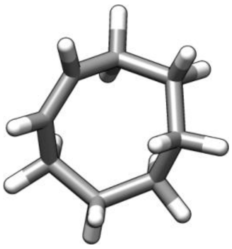

C	-0.49030	1.66000	0.23130
C	0.77580	1.36640	-0.61130
C	1.86610	0.51100	0.05980
C	1.39100	-0.83780	0.65030
C	0.58080	-1.65390	-0.32060
C	-0.74910	-1.57340	-0.44510
C	-1.65630	-0.69840	0.39120
C	-1.72910	0.78310	-0.04780
H	-0.24730	1.61770	1.30360
H	-0.79440	2.69820	0.04360
H	0.47500	0.89540	-1.55520
H	1.24470	2.31960	-0.88930
H	2.65700	0.32420	-0.68000
H	2.33550	1.08810	0.86900
H	2.27130	-1.40370	0.98160
H	0.79850	-0.63750	1.55150
H	1.13320	-2.30260	-1.00100
H	-1.23440	-2.16520	-1.22140
H	-2.67210	-1.11220	0.35630
H	-1.35330	-0.73300	1.44690
H	-2.58460	1.24010	0.46910
H	-1.96260	0.82900	-1.12100

Compound **5i** (E(RB3LYP) = −306.467759663 Ha, Lowest Frequency: 99.0875 cm^{-1}, Sum of electronic and thermal Free Energies = −306.402260 Ha.)

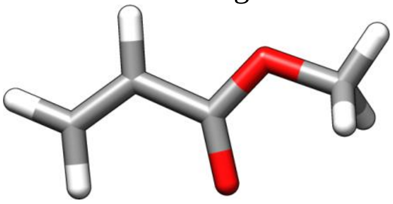

C	2.30330	-0.08880	0.00000
O	1.01720	-0.72540	-0.00000
C	-0.04360	0.11780	-0.00010
O	0.06060	1.32820	-0.00000
C	-1.31730	-0.64640	-0.00000
C	-2.49200	-0.01330	0.00000
H	3.03260	-0.89940	0.00010
H	2.42330	0.53650	-0.88900
H	2.42330	0.53650	0.88910
H	-1.24020	-1.72950	-0.00000
H	-3.43310	-0.55470	0.00010
H	-2.53010	1.07230	0.00000

Compound **5j** (E(RB3LYP) = −2689.01103286 Ha, Lowest Frequency: 108.6426 cm^{-1}, Sum of electronic and thermal Free Energies = −2688.968930 Ha.)

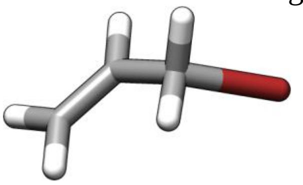

Br	-1.13490	-0.12990	-0.03430
C	0.60660	0.86040	0.02100
C	1.71130	-0.02750	0.47050
C	2.76440	-0.33700	-0.28850

H	0.75380	1.24250	-0.98860
H	0.41070	1.68130	0.71160
H	1.63000	-0.41700	1.48370
H	3.56750	-0.96690	0.08290
H	2.86380	0.02980	-1.30780

Compound **5k** (E(RB3LYP) = −210.142831072 Ha, Lowest Frequency: 88.6494 cm^{-1}, Sum of electronic and thermal Free Energies = −210.091852 Ha.)

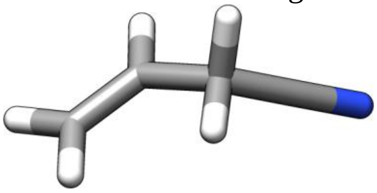

C	2.16070	-0.27360	-0.30540
C	1.05200	-0.17980	0.42520
C	-0.09000	0.76170	0.10440
C	-1.36990	0.05650	-0.05500
N	-2.37460	-0.51300	-0.17010
H	2.96470	-0.94990	-0.03020
H	2.30930	0.32070	-1.20450
H	0.92450	-0.79210	1.31600
H	-0.21420	1.50130	0.90840
H	0.12120	1.32200	-0.81400

Compound **5l** (E(RB3LYP) = −308.393595678 Ha, Lowest Frequency: 145.7241 cm^{-1}, Sum of electronic and thermal Free Energies = −308.314525 Ha.)

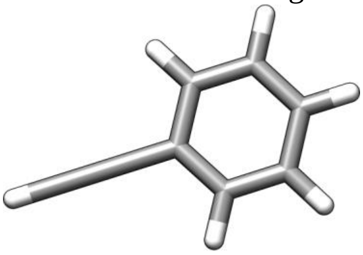

C	-3.23430	0.00000	-0.00000
C	-2.02420	-0.00010	-0.00000
C	-0.59420	-0.00000	-0.00000
C	0.11990	1.21310	-0.00000
C	1.51250	1.20860	0.00000
C	2.21300	0.00000	0.00000
C	1.51260	-1.20860	0.00000
C	0.12000	-1.21310	-0.00000
H	-4.30050	0.00000	0.00020
H	-0.42860	2.15000	-0.00000
H	2.05290	2.15130	0.00000
H	3.29960	0.00010	0.00000
H	2.05300	-2.15130	0.00000
H	-0.42850	-2.15000	-0.00000

Compound **5m** (E(RB3LYP) = −486.025144953 Ha, Lowest Frequency: 125.0675 cm^{-1}, Sum of electronic and thermal Free Energies = −485.927896 Ha.)

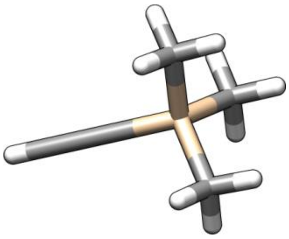

C	2.76150	0.00020	0.00010
C	1.54610	0.00020	0.00000
Si	-0.29960	0.00000	-0.00010

C	-0.90060	-1.52950	-0.93230
C	-0.90100	-0.04280	1.79050
C	-0.90120	1.57210	-0.85830
H	3.82940	0.00020	0.00010
H	-0.54530	-2.45090	-0.45710
H	-1.99690	-1.56500	-0.95400
H	-0.54510	-1.52990	-1.96880
H	-0.54570	0.82900	2.35140
H	-1.99730	-0.04380	1.83190
H	-0.54570	-0.94070	2.30870
H	-0.54590	1.62190	-1.89370
H	-1.99750	1.60830	-0.87800
H	-0.54600	2.46980	-0.33980

Compound **5n** (E(RB3LYP) = −344.520086714 Ha, Lowest Frequency: 53.7728 cm^{-1}, Sum of electronic and thermal Free Energies = −344.452723 Ha.)

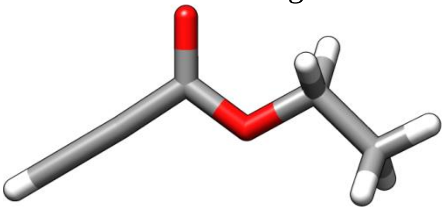

C	2.75490	-0.75740	-0.00020
C	1.67890	0.31100	0.00020
O	0.39680	-0.36620	0.00010
C	-0.68220	0.43880	-0.00000
O	-0.64500	1.65110	-0.00010
C	-1.90860	-0.33640	-0.00000
C	-2.95820	-0.93250	-0.00000
H	2.67600	-1.39300	0.88740
H	2.67590	-1.39250	-0.88810
H	3.74340	-0.28540	-0.00010
H	1.73380	0.95460	0.88390
H	1.73360	0.95510	-0.88320
H	-3.88630	-1.45930	0.00010

References

1. Tanaka, K.; Masuda, H.; Mitsuhashi, K. *Bull. Chem. Soc. Jpn.*, **1984**, *57*, 2184-2191. for the melting point 44-45.5 °C and James W. Pavlik, Jennifer A. Lowell, Vuthichai Ervithayasuporn *J. Heterocyclic Chem.* **2005**, *42*, 1253-1255 for the NMR data.

2.(a) Kohn, W.; Sham, L. J. *Phys. Rev.* **1965**, *140*, A1133. (b) Hohenberg, P.; Khon, W. *Phys Rev.* **1964**, *136*, B864.

3. (a) Becke, A. D. *J. Chem. Phys.* **1993**, *98*, 5648. (b) Lee, C.; Yang, W.; Parr, R. G. *Phys. Rev. B* **1988**, *37*, 785.

4 Hehre, W. J.; Radom, L.; Schleyer, P. v. R.; Pople, J. A. *Ab Initio Molecular Orbital Theory*; Wiley: New York, 1986.

5. Gaussian 09, Revision A.02. Frisch, M. J.; Trucks, G. W.; Schlegel, H. B.; Scuseria, G. E.; Robb, M. A.; Cheeseman, J. R.; Scalmani, G.; Barone, V.; Mennucci, B.; Petersson, G. A.; Nakatsuji, H.; Caricato, M.; Li, X.; Hratchian, H. P.; Izmaylov, A. F.; Bloino, J.; Zheng, G.; Sonnenberg, J. L.; Hada, M.; Ehara, M.; Toyota, K.; Fukuda, R.; Hasegawa, J.; Ishida, M.; Nakajima, T.; Honda, Y.; Kitao, O.; Nakai, H.; Vreven, T.; Montgomery, J. A., Jr.; Peralta, J. E.; Ogliaro, F.; Bearpark, M.; Heyd, J. J.; Brothers, E.; Kudin, K. N.; Staroverov, V. N.; Kobayashi, R.; Normand, J.; Raghavachari, K.; Rendell, A.; Burant, J. C.; Iyengar, S. S.; Tomasi, J.; Cossi, M.; Rega, N.; Millam, J. M.; Klene, M.; Knox, J. E.; Cross, J. B.; Bakken, V.; Adamo, C.;

Jaramillo, J.; Gomperts, R.; Stratmann, R. E.; Yazyev, O.; Austin, A. J.; Cammi, R.; Pomelli, C.; Ochterski, J. W.; Martin, R. L.; Morokuma, K.; Zakrzewski, V. G.; Voth, G. A.; Salvador, P.; Dannenberg, J. J.; Dapprich, S.; Daniels, A. D.; Farkas, Ö.; Foresman, J. B.; Ortiz, J. V.; Cioslowski, J.; Fox, D. J. Gaussian, Inc., Wallingford CT, 2009.

6. (a) Chimera is developed by the Resource for Biocomputing, Visualization, and Informatics at the University of California, San Francisco, with support from the National Institutes of Health (National Center for Research Resources grant 2P41RR001081, National Institute of General Medical Sciences grant 9P41GM103311). (b) Pettersen, E. F.; Goddard, T. D.; Huang, C. C.; Couch, G. S.; Greenblatt, D. M.; Meng, E. C.; Ferrin, T. E. *J. Comput. Chem.* **2004**, *25*, 1605.
